# Identification of a Nuclear Mitochondrial-Related Multi-Genes Signature to Predict the Prognosis of Bladder Cancer

**DOI:** 10.3389/fonc.2021.746029

**Published:** 2021-10-06

**Authors:** Xuewen Jiang, Yangyang Xia, Hui Meng, Yaxiao Liu, Jianfeng Cui, Huangwei Huang, Gang Yin, Benkang Shi

**Affiliations:** Department of Urology, Qilu Hospital, Cheeloo College of Medicine, Shandong University, Ji’nan, China

**Keywords:** bladder cancer, nuclear mitochondria-related genes, TCGA, signature, survival, prognosis, nomogram

## Abstract

**Introduction:**

Bladder cancer (BC) is one of the most prevalent urinary cancers, and its management is still a problem causing recurrence and progression, elevating mortality.

**Materials and Methods:**

We aimed at the nuclear mitochondria-related genes (MTRGs), collected from the MITOMAP: A Human Mitochondrial Genome Database. Meanwhile, the expression profiles and clinical information of BC were downloaded from the Cancer Genome Atlas (TCGA) as a training group. The univariate, multivariate, and the least absolute shrinkage and selection operator (LASSO) Cox regression analyses were used to construct a nuclear mitochondrial-related multi-genes signature and the prognostic nomogram.

**Results:**

A total of 17 nuclear MTRGs were identified to be correlated with the overall survival (OS) of BC patients, and a nuclear MTRGs signature based on 16 genes expression was further determined by the LASSO Cox regression analysis. Based on a nuclear MTRGs scoring system, BC patients from the TCGA cohort were divided into high- and low- nuclear MTRGs score groups. Patients with a high nuclear MTRGs score exhibited a significantly poorer outcome (median OS: 92.90 *vs* 20.20 months, p<0.0001). The nuclear MTRGs signature was further verified in three independent datasets, namely, GSE13507, GSE31684, and GSE32548, from the Gene Expression Omnibus (GEO). The BC patients with a high nuclear MTRGs score had significantly worse survival (median OS in GSE13507: 31.52 *vs* 98.00 months, p<0.05; GSE31684: 32.85 months *vs* unreached, p<0.05; GSE32548: unreached *vs* unreached, p<0.05). Furthermore, muscle-invasive bladder cancer (MIBC) patients had a significantly higher nuclear MTRGs score (p<0.05) than non-muscle-invasive bladder cancer (NMIBC) patients. The integrated signature outperformed each involved MTRG. In addition, a nuclear MTRGs-based nomogram was constructed as a novel prediction prognosis model, whose AUC values for OS at 1, 3, 5 years were 0.76, 0.75, and 0.75, respectively, showing the prognostic nomogram had good and stable predicting ability. Enrichment analyses of the hallmark gene set and KEGG pathway revealed that the E2F targets, G2M checkpoint pathways, and cell cycle had influences on the survival of BC patients. Furthermore, the analysis of tumor microenvironment indicated more CD8+ T cells and higher immune score in patients with high nuclear MTRGs score, which might confer sensitivity to immune checkpoint inhibitors.

**Conclusions:**

Not only could the signature and prognostic nomogram predict the prognosis of BC, but it also had potential therapeutic guidance.

## Background

Bladder cancer (BC) is one of the most frequent genitourinary carcinoma worldwide, with an estimated number of annually newly diagnosed cases beyond 550,000 and deaths beyond 200,000 (1). BC contains a spectra of diseases, including non-muscle invasive (NMIBC), muscle invasive (MIBC), and metastatic diseases, whose 5-year survival ranged from 96 to 6% (2). For patients with MIBC, radical cystectomy remains the standard management, but the outcomes differed among patients. The genomic heterogeneity may lead to the distinct outcomes of MIBC patients, and different predictions of the progression risk. Many studies based on its transcriptome profiling have been implemented to classify MIBC into different subtypes, such as luminal, basal, or neuroendocrine (3). However, past results on molecular classification derived from different methods and datasets and the diversity of the classification posed limits on the further clinical application (4).

Recently, several studies explored the more advanced clinical and molecular biomarkers to improve the management and treatment of BC. Comprehensive genetic profiling has successfully identified prevalent genetic alterations, such as *FGFR3* (5), DNA Damage repair gene (6), and *PIK3CA* (7) in predicting the prognosis of BC. And transcriptome profiling suggested the abnormal expression of single markers, such as *KIF20A* (8), non-coding RNA (9), Matrix Metalloproteinase 11 (10), was related to bad survival of BC patients. In addition, signatures based on multiple gene expressions, such as immune-related genes (11), hypoxia-related genes (12), and an EMT-related gene signature (13), were highly associated with the prognosis of BC patients. By contrast to the single molecular biomarker, multi-genes biomarker had stronger predicting capabilities with high accuracy and sensitivity (11–13). Nevertheless, different BC patients might have different clinical features and molecular characteristics in the same clinical stage (14), owing to the individual heterogeneity. Therefore, there is an urgent need for more rigorous prediction prognosis models to help promote BC management and the development of precision medicine.

Mitochondria, known as the “powerhouse” in the cell, play essential roles in many cell activities, including energy metabolism, signaling transduction, cell growth and death (15). Next-generation sequencing data revealed some molecular characteristics (containing nuclear mitochondrial-related DNA/RNA and mitochondrial DNA/RNA) of mitochondrial diseases. Non-structural nuclear MTRGs, such as *NDUFAF1*, *COA5*, and *COA6*, were correlated with cardioencephalomyopathy; besides, structural nuclear MTRGs, such as *NDUFS2*, *NDUFB10*, and *DUFV2*, were correlated with cardiomyopathy (16). In addition, other studies have comprehensively demonstrated that mitochondrial dysfunctions are intrinsically associated with carcinogenesis (17, 18). Pan-cancer genome analysis showed that nuclear mitochondrial and mitochondrial genomic alterations were related to mitochondrial functions were correlated with 38 tumor types (19). Moreover, other studies had explored the roles of mitochondrial genes and uncovered the associations between genomic alterations and the prognosis of cancer patients. However, mitochondrial molecular alterations (including mitochondrial DNA copy number, structural variations, microsatellite instability) exhibited the unstable efficacy in predicting the prognosis of colorectal cancer, probably because of a lack of in-depth researches (20). To date, the role of nuclear MTRGs in prognosis prediction for various cancers is scarcely known. Therefore, our study aims to investigate whether genetic and transcriptomic profiling of MTRGs is correlated with the survival of BC patients.

## Materials and Methods

### Data Acquisition and Processing

The TCGA bladder cancer data (blca_tcga_pub_2017), containing genomic alternations, mRNA expression profiles, and clinicopathological features, were downloaded from the cBioPortal (http://www.cbioportal.org/) as a training group of 413 muscle-invasive BC patients. For validation, three independent datasets, including GSE13507 (165 patients: 62 MIBC and 103 NMIBC), GSE31684 (93 patients: 79 MIBC and 14 NMIBC), and GSE32548 (131 patients, the numbers of MIBC and NMIBC were undescribed), were derived from the Gene Expression Omnibus (GEO, https://www.ncbi.nlm.nih.gov/geo/). The clinicopathological data of patients are presented in **Table 1**.

**Table 1:** Characteristics of 413 patients with bladder cancer from the TCGA.

Variable		Number of Patient (%)
Total		413
Gender	Male	304 (73.61%)
	female	108 (26.15%)
Age	Median (range)	69 (34–90)
Pathological staging	Muscle-invasive	413 (100%)
Histological grading	High grade	388 (93.95%)
	Low grade	21 (5.08%)
Clinical Stage	I	2 (10.9%)
	II	131 (31.72%)
	III	141 (34.14%)
	IV	136 (32.93%)
T stage	T0	1 (0.01%)
	T1	3 (0.01%)
	T2	121 (29.30%)
	T3	195 (47.22%)
	T4	59 (14.29%)
N stage	N0	239 (57.87%)
	N1	47 (11.38%)
	N2	76 (18.40%)
	N3	8 (0.02%)
M stage	M0	196 (47.46%)
	M1	11 (0.03%)

Nuclear genes involved in mitochondrial disease were obtained from the MITOMAP: A Human Mitochondrial Genome Database (http://www.mitomap.org, last update: January 15^th^, 2021), and all of these nuclear MTRGs (including 33 structural nuclear MTRGs and 114 non-structural nuclear MTRGs) were integrated as a nuclear mitochondria-related gene set.

### Differentially Expressed Nuclear MTRGs Related to BC, and Analysis of Genomic Alterations in BC

The RNA-sequencing data of BC (404 samples) and normal people (28 samples) were collected from the GEPIA (http://gepia2.cancer-pku.cn/) to screen out differentially expressed MTRGs [|log2-fold change (FC)| >1, Q-value<0.01].

Nuclear mitochondria-related gene data were derived using cBioPortal to explore genomic alterations (in-frame indels, missense mutation, splice mutation, truncating mutation, amplification, deep deletion, and copy number alteration) among BC patients from the TCGA cohort.

### Survival Analysis and Construction of a Prognostic Nuclear MTRGs Signature

The TCGA bladder cancer dataset was analyzed to determine whether the nuclear MTRGs alterations correlated with survival of BC patients *via* univariate Cox proportional hazards regression analysis, which was conducted in R studio (v. 3.4.3, https://rstudio.com/). Then, least absolute shrinkage and selection operator (LASSO) Cox regression with 10-fold cross-validation was conducted by using the “glmnet” package in R studio (21). Meanwhile, multivariate Cox regression analysis was further utilized to identify prognostic nuclear MTRGs and to construct a nuclear MTRGs signature.

The nuclear MTRGs score was calculated by the following formula: nuclear MTRGs score = gene A expression × γ_A_ + gene B expression × γ_B_ + gene C expression × γ_C_ +… + gene Z expression × γ_Z_, where γ_Z_ represents the coefficient for each nuclear MTRG in the multivariate Cox regression model. The median nuclear MTRGs score served as a cutoff value to divide the patients into two groups, high and low nuclear MTRGs score groups, respectively.

The Kaplan-Meier (KM) curves were drawn by using the “survival” package in R studio. And the area under ROC curve (AUC) was calculated to evaluate the prognostic ability of the defined nuclear MTRGs signature *via* using the “timeROC” package in R studio. In addition, univariate and multivariate Cox regression analyses were implemented to identify the prognostic values for the signature and clinicopathological features. The nomogram and calibration plots were built by using the “rms” package in R studio.

### Enrichment Analyses of Hallmark Gene Set and KEGG Pathway

Enrichment analysis was conducted by using the “clusterProfiler” package (22) in R studio to identify significant key genes and/or common pathways involved in tumor progression and metastasis of BC. We mainly focused on Hallmark gene set enrichment analysis (http://www.gsea-msigdb.org/gsea/) and Kyoto Encyclopedia of Genes and Genomes (KEGG, https://www.kegg.jp/) pathway enrichment analysis, which were visualized by using the “ggplot2” package in R studio. The threshold was defined at a p-value <0.05.

### Evaluation of the Immune Cell Infiltration in BC

A deconvolution algorithm [TIMER, http://timer.cistrome.org/, (23)] was employed to survey the infiltrating immune cells in the tumor microenvironment (TME) between high and low nuclear MTRGs score groups, by using the transcriptomic data from the TCGA cohort. Meanwhile, the stromal score, immune score, and ESTIMATE score were calculated to further clarify the infiltrated lymphocytes (24).

### Statistical Analyses

**Figure 1:**
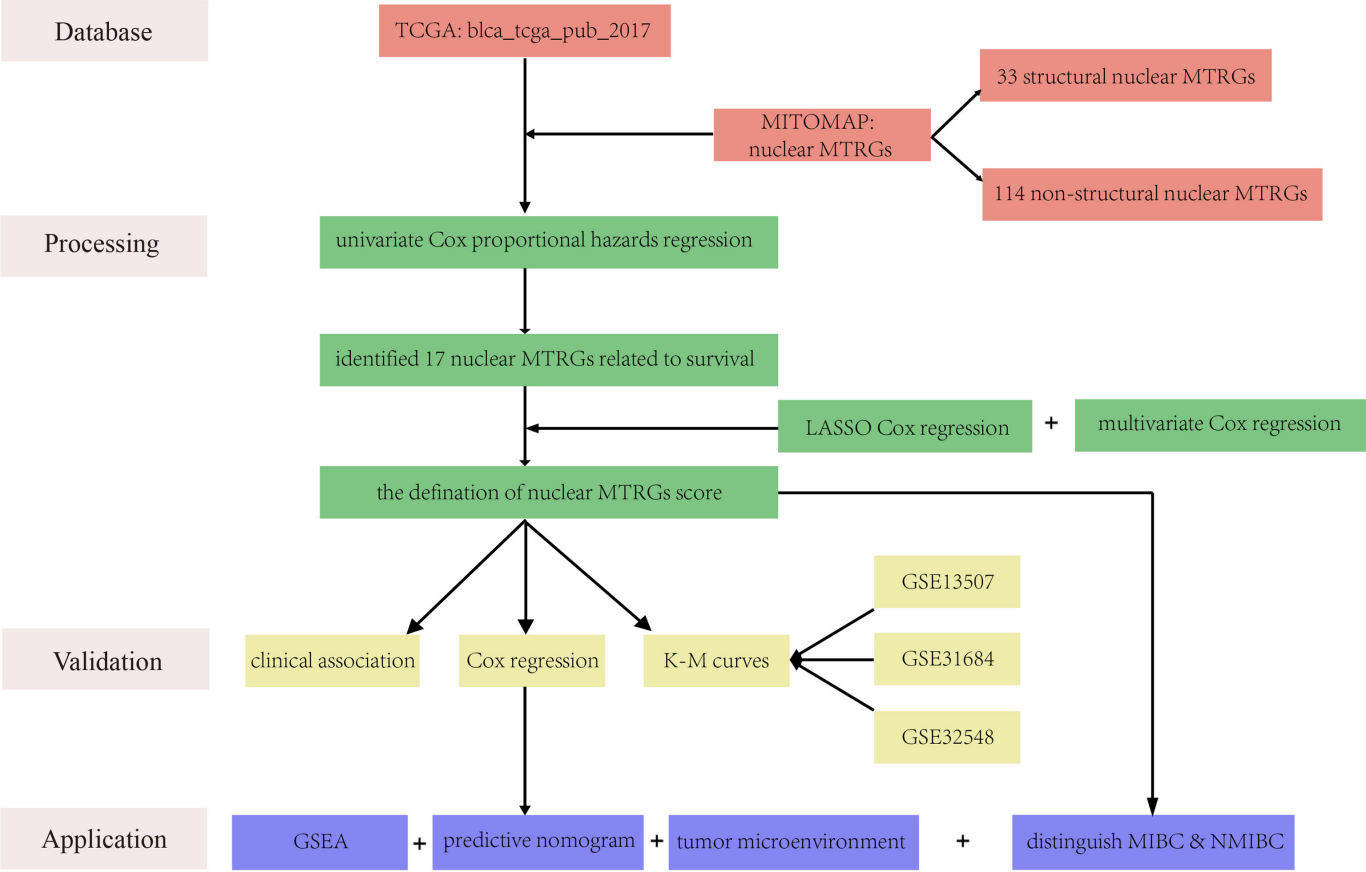
A work flowchart of the establishment of a nuclear MTRGs-based signature in BC.

A work flowchart is exhibited in **Figure 1**. Besides, we performed statistical analyses of Chi-square, Fisher test, and Wilcoxon rank test in R studio. A log-rank test was used to estimate the Kaplan-Meier curves of survival analysis between the high and low nuclear MTRGs score groups. A (adjust) p-value <0.05 was considered statistically significant.

## Results

### Analysis of the Differentially Expressed Nuclear MTRGs (Normal *vs* BC), and MTRGs Genomic Alterations in BC

Comparison of the mRNA expression data between BC patients and normal control samples from the TCGA cohort was done, displaying that 4% (6/147) of MTRGs was differentially expressed [**Figure S1**, |log_2_(FC)| >1 and Q-value<0.01]. Then we further explored the genomic alterations in these BC patients. Nearly 97% (395/408) of BC patients had altered nuclear MTRGs expression levels (**Figure S2A**). Meanwhile, the MTRGs mutation in BC patients from the TCGA cohort was analyzed, showing that 53% (218/412) of BC patients were identified with at least one MTRGs mutation (**Figure S2B**). Furthermore, copy number alterations occurred in nearly 70% (287/408) of BC patients from the TCGA cohort (**Figure S2C**).

### Identification of the Nuclear MTRGs Correlating With Survival of BC Patients

**Figure 2:**
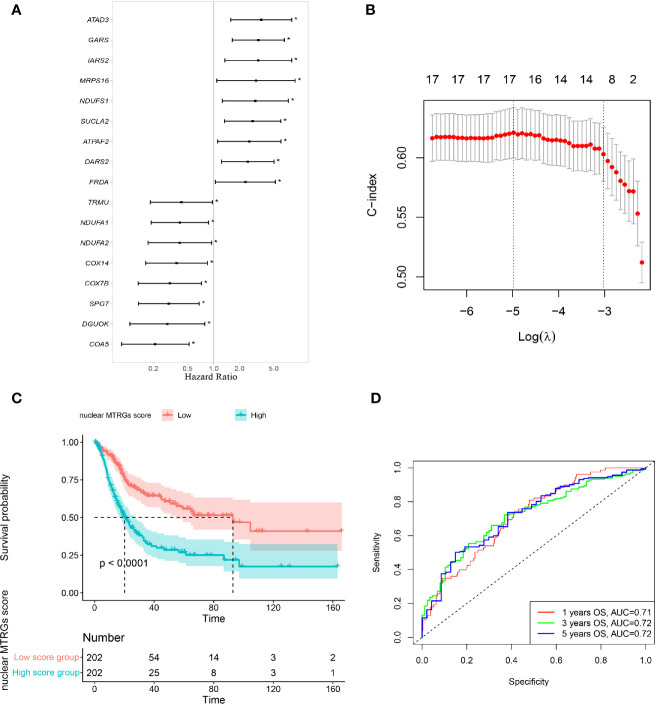
Construction of a nuclear MTRGs signature. **(A)** The identification of the nuclear mitochondria-related genes associated with survival of BC patients *via* univariate Cox regression analysis. **(B)** The LASSO analysis determined a nuclear 16-MTRGs signature. **(C)** Comparison of OS between the high and low nuclear MTRGs score groups *via* Kaplan-Meier curves. **(D)** The ROC curves for OS at 1, 3, and 5 years. *p < 0.05.

We conducted univariate COX proportional hazards regression analysis with nuclear mitochondria-related genes, and 17 of 146 genes were found to be significantly correlated with the survival of BC patients (**Figure 2A**). The overexpression of nine nuclear MTRGs, including *ATAD3*, *GARS*, *IARS2*, *MRPS16*, *NDUFS1*, *SUCLA2*, *ATPAF2*, *DARS2*, and *FRDA*, significantly related to a worse prognosis of BC patients (p<0.05, **Figure 2A**). On the other hand, the overexpression of eight nuclear MTRGs, including *TRMU*, *NDUFA1*, *NDUFA2*, *COX14*, *COX7B*, *SPG7*, *DGUOK*, and *COA5*, were significantly correlated with improved prognosis of BC patients (p<0.05, **Figure 2A**).

### Construction and Evaluation of a Nuclear MTRGs Prognostic Signature

The LASSO regression analysis *via* 10-fold cross-validation demonstrated that 16 out of 17 identified nuclear MTRGs related to patients’ survival (**Figure 2B**), and these identified genes were further analyzed with the multivariate Cox regression analysis, and their coefficients are exhibited in **Table 2**. Moreover, BC patients from the TCGA cohort with a high nuclear MTRGs score (N=202) exhibited the poorer outcomes (median OS: 92.90 *vs* 20.20 months, p<0.0001, **Figure 2C**), and the AUC for OS at 1-, 3-, 5-year was 0.71, 0.72, and 0.72, respectively (**Figure 2D**). Moreover, by comparing the AUC of our nuclear MTRGs signature with single identified MTRG for OS at 1-, 3-, 5-year, the results indicated that the integrated signature had better prediction efficacy (**Figure S3**).

**Table 2:** Hazard ratio and coefficient of identified nuclear MTRGs in signature.

Gene	Hazard ratio	95% CI	Coefficient	p-value
*NDUFS1*	3.0537	1.26–7.40	0.1987	0.013
*NDUFA1*	0.4054	0.19–0.87	0.7709	0.021
*COX7B*	0.3099	0.13–0.72	−1.6085	0.007
*COX14*	0.3710	0.16–0.85	−0.0778	0.019
*COA5*	0.2098	0.08–0.52	−0.6124	0.001
*ATPAF2*	2.5996	1.11–6.08	1.1018	0.027
*DGUOK*	0.2902	0.11–0.79	−0.2726	0.015
*SUCLA2*	2.8469	1.34–6.03	0.7184	0.006
*DARS2*	2.4925	1.24–5.03	0.0788	0.011
*GARS*	3.3122	1.65–6.66	0.5303	0.001
*IARS2*	3.3020	1.35–8.06	0.5714	0.008
*TRMU*	0.4224	0.18–0.97	−0.5094	0.042
*MRPS16*	3.1102	1.09–8.86	0.7043	0.034
*FRDA*	2.3424	1.05–5.23	0.4908	0.038
*SPG7*	0.3016	0.13–0.68	−0.1571	0.004
*ATAD3*	3.5811	1.59–8.09	1.1506	0.002

CI, confidence interval.

### Validation of the Nuclear MTRGs Prognostic Signature

**Figure 3:**
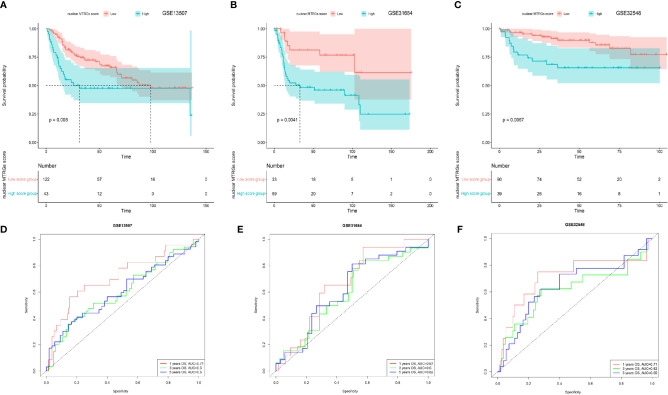
The comparison of the OS and AUC values between the high and low nuclear MTRGs score groups. The analysis of Kaplan-Meier curves for BC patients assigned to high and low nuclear MTRGs score groups in GSE13507 **(A)**, GSE31684 **(B)**, and GSE32548 **(C)**. The corresponding ROC curves for OS at 1, 3, and 5 years were indicated in GSE13507 **(D)**, GSE31684 **(E)**, and GSE32548 **(F)**.

Furthermore, the signature scoring system was verified in the three independent datasets from GEO database, including GSE13507, GSE31684, and GSE32548. It was shown that BC patients with a high nuclear MTRGs score had significantly worse survival in all these independent validation cohorts (a median OS in GSE13507: 31.52 *vs* 98.00 months, p<0.05; GSE31684: 32.85 months *vs* unreached, p<0.05; GSE32548: unreached *vs* unreached, p<0.05; **Figures 3A–C**). In addition, the AUC for OS at 1-, 3-, 5-year are as follows: GSE13507: 0.71, 0.60, 0.60 (**Figure 3D**); GSE31684: 0.67, 0.60, 0.62 (**Figure 3E**); and GSE32548: 0.71, 0.62, 0.65 (**Figure 3F**), respectively.

**Figure 4:**
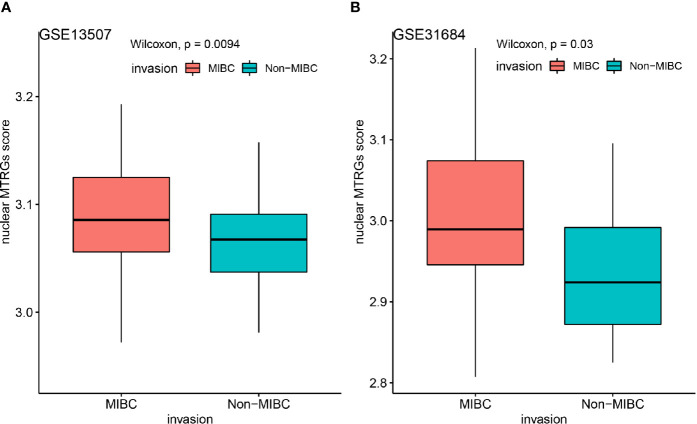
Comparing the nuclear MTRGs score of the NMIBC and MIBC groups in two independent datasets of GSE13507 **(A)**, GSE31684 **(B)**.

Meanwhile, as GSE13507 and GSE31684 datasets having comparable data of both NIMBC and MIBC, we further applied the nuclear MTRGs scoring system to figure out whether there existed some significant differences between these two subtypes of the disease. Notably, MIBC samples had a significantly higher nuclear MTRGs score than NMIBC (a median nuclear MTRGs score in GSE13507: 3.09 *vs* 3.06, GSE31684: 2.99 *vs* 2.92, p<0.05, **Figure 4**).

### Survival Analysis of the BC Patients With High or Low Nuclear MTRGs Score in the Different Histopathological Groups

**Figure 5:**
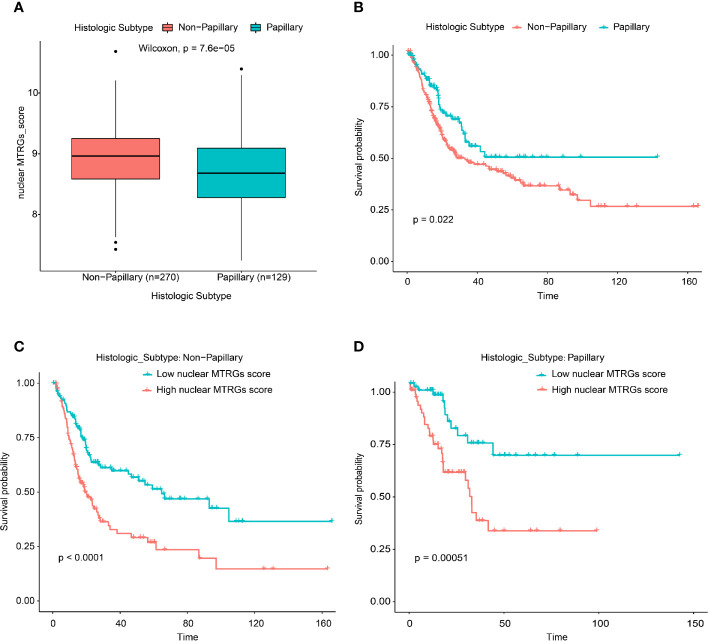
Comparison of the nuclear MTRGs score between BC patients with or without the papillary features, and the survival analysis in the different histologic subtype groups.

In the TCGA bladder cancer dataset, there were two defined histologic subtype groups (the group with papillary features or not). We observed that the BC patients without papillary histological features have significantly higher nuclear MTRGs score than those one with the papillary histological features (p<0.0001, **Figure 5A**). While the BC patients without papillary-related features had the worse survival (median overall survival: 28.22 *vs* 44.28 months, p<0.05, **Figure 5B**). In addition, the BC patients with high nuclear MTRGs score had the poorer survival (non-papillary: 19.02 *vs* 64.75 months, p<0.0001; papillary: 32.00 months *vs* unreached, p<0.001, **Figures 5C, D**) no matter in the non-papillary or papillary group.

In the present study, there were 298 male BC patients, and 81/298 of these patients presented concomitant prostate cancer (PCa). By the statistical analysis, there was no significant difference of the nuclear MTRGs score between the BC patients who presented with concomitant prostate cancer and those who did not (p=0.087, **Figure S4A**). Moreover, we observed no significant difference of the overall survival between the BC patients who presented with concomitant prostate cancer and those who did not (p=0.57, **Figure S4B**). However, the BC patients with high nuclear MTRGs score had the poorer survival (not concomitant PCa: 17.97 months *vs* unreached, p<0.0001; concomitant PCa: 25.56 months *vs* unreached, p<0.01, **Figures S4C, D**) no matter the BC patients presented with concomitant prostate cancer or not.

### The Clinical Features of BC Patients in the High and Low Nuclear MTRGs Score Groups

**Figure 6:**
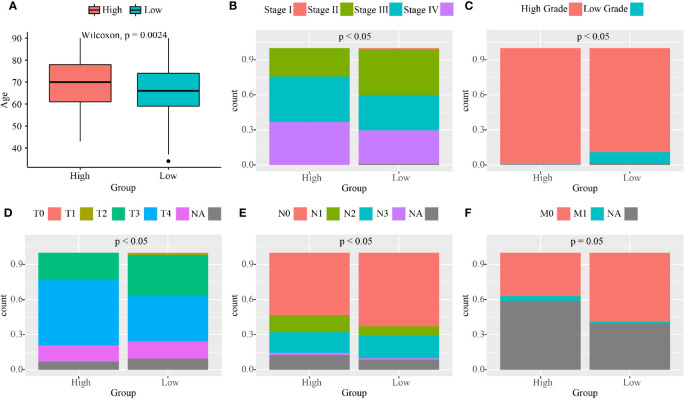
The analyses of clinical features between the high and low nuclear MTRGs score groups from the TCGA cohort. **(A)** Boxplots exhibit the age at diagnosis for the high and low nuclear MTRGs score groups (p=0.0024). Percentage-staked bar plots show the distribution of BC patients with the different clinical stage **(B)**, histological grade **(C)**, tumor stage **(D)**, lymph node status **(E)**, and tumor metastasis **(F)** between the high and low nuclear MTRGs score groups (p<0.05 was considered as significant; NA, not applicable).

We then investigated the clinical features of BC patients in the high and low nuclear MTRGs score groups. Patients in the high nuclear MTRGs score group have older age at diagnosis (median age at diagnosis: 70 *vs* 66 years old, p<0.05, **Figure 6A**). Remarkably, more BC patients with a high nuclear MTRGs score had advanced clinical stages (0 *vs* 1.01% in stage I, 24.26 *vs* 39.51% in stage II, 39.11 *vs* 30.50% in stage III, 36.63 *vs* 29.01% in stage IV, p<0.05, **Figure 6B**), compared to those with low nuclear MTRGs score. According to the analysis of histological grading, more high histological grading diseases were presented in the high MTRGs score group (99.51 *vs* 90.45% in high histological grading, 0.49 *vs* 9.55% in low histological grading, p<0.05, **Figure 6C**). Moreover, TNM stages were further analyzed, indicating that patients with a high MTRGs score were significantly related to aggressive tumor and metastasis (**Figures 6D–F**). We found a significantly decreased number of patients at T0–T2 but significantly more patients with disease at T3–T4 stage, which were presented in the high nuclear MTRGs score group (25.01 *vs* 40.44% at T0–T2, 74.99 *vs* 50.56% at T3–T4, p<0.05, **Figure 6D**). However, there was no statistically significant difference between the two groups in lymph node metastasis (77.41 *vs* 77.29% at N0–N1, 22.59 *vs* 22.71% at N2–N3, p>0.05, **Figure 6E**). By statistical analysis, only eight and three BC patients with a high or low nuclear MTRGs score were at M1 stage, respectively. A weakly significant difference was observed in the number of patients with long-distant metastasis in the high nuclear MTRGs score group (9.64 *vs* 2.46% at M1, p=0.05, **Figure 6F**).

**Figure 7:**
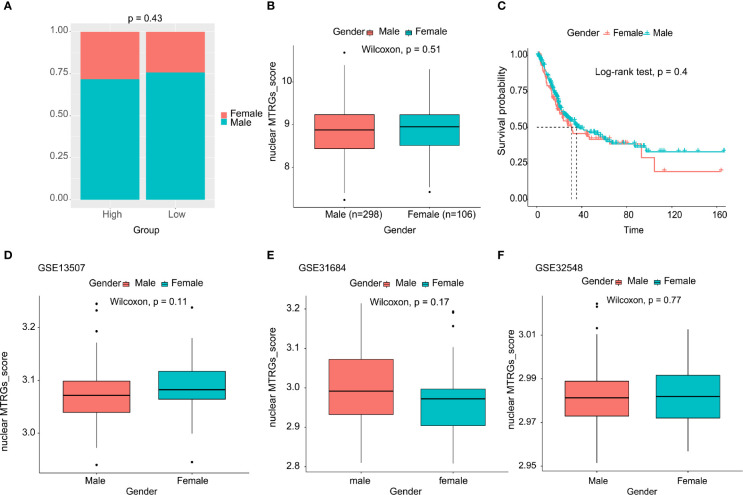
Exploration of the clinical feature of gender between the high and low nuclear MTRGs score groups, and the investigation for the overall survival between the male and female BC group.

As is known, gender plays an important role in mitochondrial dysfunction (25, 26). However, we did not obtain evidence for significant differences in the clinical feature of gender in BC (p=0.43, **Figure 7A**). Subsequently, we explored whether there existed any statistically significant differences of the identified nuclear MTRGs score between the male and female group, whereas it was found that there was no statistically significant difference in the nuclear MTRGs scores between the male and female group, either (p=0.51, **Figure 7B**). Moreover, no any statistically significant differences were observed in the overall survival between the male and female group (p=0.40, **Figure 7C**). In addition, this result was further validated in another three independent cohort, GSE13507, GSE31684, and GSE32548, from the GEO (p>0.05, **Figures 7D–F**). But it was noteworthy that the male BC patients with high nuclear MTRGs score had the worse overall survival (median overall survival: 19.38 months *vs* unreached, p<0.0001, **Figure S5A**). It was also verified in another three independent cohorts (GSE13507: 98.00 *vs* 134.97 months, p<0.05; GSE31684: 51.52 months *vs* unreached, p<0.05; GSE31684: 51.52 months *vs* unreached, p<0.01, **Figures S5B–D**). For the female BC group, in the TCGA bladder cancer cohort, the female BC patients with high nuclear MTRGs score had the worse overall survival as well (median overall survival: 16.16 *vs* 64.75 months, p<0.0001, **Figure S5E**). However, it was observed that there were no any statistically significant differences of overall survival in the cohorts of GSE13507, GSE31684, and GSE31684 (p>0.05, **Figures S5F–H**).

### Gene Mutation Profiles in the High and Low Nuclear MTRGs Score Groups

**Figure 8:**
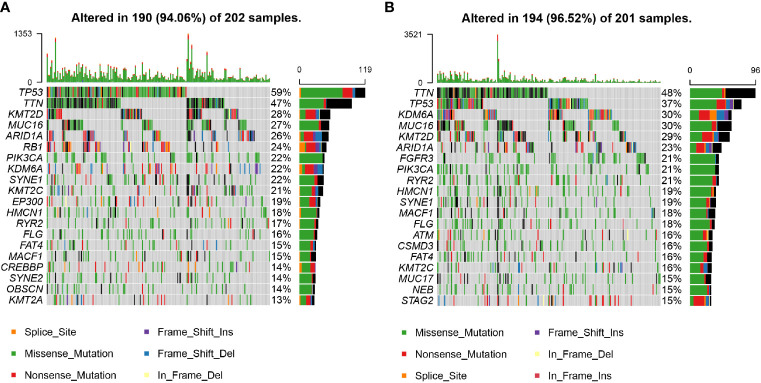
The profiles of mutated genes between the high and low nuclear MTRGs score groups from the TCGA cohort. **(A)** The mutation profile of the top 20 most frequently mutated genes in the high MTRGs score group. **(B)** The mutation profile of the top 20 most frequently mutated genes in the low MTRGs score group.

The profiles of the top 20 most frequently mutated genes in the high and low nuclear MTRGs score group are manifested in **Figure 8**. It could be seen that the prevalence of genes was distinct between the high and low nuclear MTRGs score groups. The high nuclear MTRGs group demonstrated a relatively higher prevalence of *TP53* (59%, **Figure 8A**), compared to that (37%) in the low nuclear MTRGs group (**Figure 8B**). However, by subsequent statistical analysis, there is no significant difference in the prevalence of *TP53* as well as any other genes between the high and low nuclear MTRGs score groups (adjusted p-value>0.05, **Table S1**).

### Construction of a Nomogram

**Figure 9:**
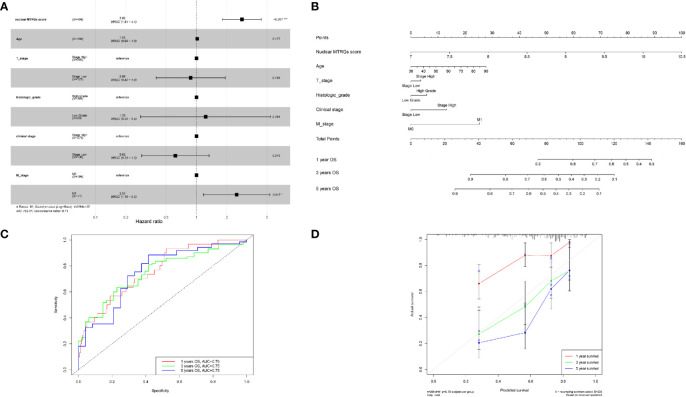
Construction and evaluation of a nomogram. **(A)** The multivariate Cox regression analysis for the nuclear MTRGs signature and clinical indexes. **(B)** The constructed nomogram to predict the OS possibilities of BC patients. **(C)** The ROC curves of the nomogram for OS at 1, 3, and 5 years. **(D)** Calibration blots indicated the agreement between the predicted OS and actual OS at 1, 3, and 5 years. *p < 0.05, ***p < 0.001.

Overall, the multivariate Cox regression analysis further showed that the established nuclear MTRG-based signature was more effective to predict the prognosis of BC (p<0.001, **Figure 9A**). Then a nomogram was constructed by combining the nuclear MTRGs signature with clinicopathological parameters, including age at diagnosis, clinical stage, T stage, M stage, and histological grading (**Figure 9B**). The concordance index (C-index) of the nomogram was 0.706 (95% CI=0.648–0.764). According to the nomogram, every evaluated patient would have a nomogram score that was associated with the prognosis of BC patients. Additionally, the AUC values of 1-, 3-, 5-year OS for the nomogram were 0.76, 0.75, and 0.75, respectively, showing our model had good and stable predicting ability (**Figure 9C**). And the calibration plots displayed the agreement between the predicted OS and actual OS (**Figure 9D**).

### Enrichment Analyses of Hallmark Gene Set and KEGG Pathway

**Figure 10:**
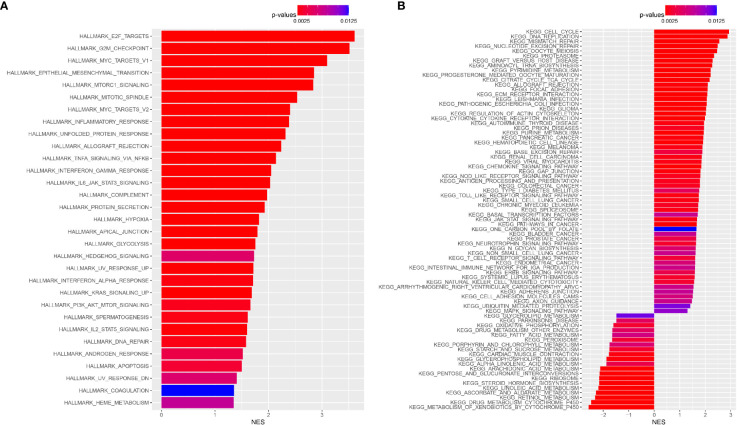
The gene set enrichment analysis. **(A)** The enrichment analysis of Hallmark gene set, and the enrichment analysis of KEGG pathway **(B)** between the high and low nuclear MTRGs score groups.

To elucidate functional differences among patients from the TCGA cohort, we further investigated the high and low nuclear MTRGs score groups. By performing the hallmark gene set enrichment analysis, it was found that high nuclear MTRGs score group were highly enriched in E2F targets, G2M checkpoint, Myc targets V1, epithelial-mesenchymal transition, mTORC1 signaling, mitotic spindle, Myc targets V2, etc (p<0.05, **Figure 10A**). Besides, KEGG pathway enrichment analysis revealed that the high nuclear MTRGs score group had a significant abundance of cell cycle, DNA replication, mismatch repair, etc. (p<0.05, **Figure 10B**).

### Evaluation of TME in the High and Low Nuclear MTRGs Score Group

**Figure 11:**
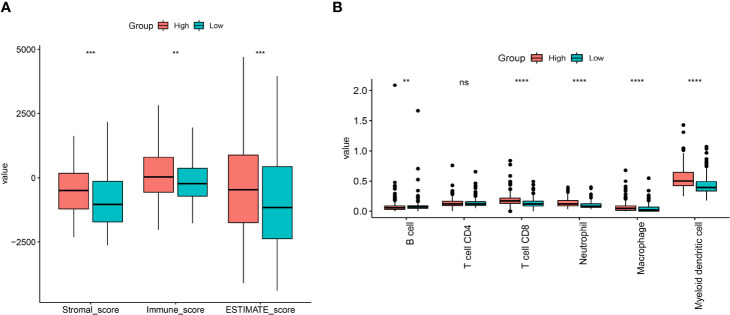
The immune cell infiltration in BC. **(A)** The analysis of the stromal score, immune score, and ESTIMATE score between the high and low nuclear MTRGs score groups. **(B)** The estimate of immune cell infiltration *via* a deconvolution algorithm of TIMER. **p < 0.01, ***p < 0.001, ****p < 0.0001, ns, not significant.

The stromal score, immune score, and ESTIMATE score represented the infiltration of stromal/immune cells. The stromal score, immune score, and ESTIMATE score were all significantly higher in the high nuclear MTRGs score group (p<0.01, **Figure 11A**). In addition, an estimated abundance of infiltrated immune cells *via* TIMER analysis revealed that CD8+ T cells, neutrophils, macrophages, and myeloid dendritic cells were significantly more enriched in the high nuclear MTRGs score group (p<0.0001, **Figure 11B**), whereas a significantly higher abundance of B cells was shown in the low nuclear MTRGs score group (p<0.01, **Figure 11B**).

## Discussions

BC is one of the most common cancers in the urinary tract, with high morbidity and mortality worldwide. Moreover, BC patients had distinct outcomes owing to the tumor heterogeneity. In recent decades, the management of BC has been unceasingly promoted, but BC patients with advanced stages still had a short survival time (27). Thus, there is much room for improvement in BC management, and effective prediction prognosis models are important and urgently needed. In this study, we explored the associations between the gene expression of nuclear MTRGs and patients’ survival. We identified a nuclear 16-MTRG signature with better specificity and sensitivity for the prognosis prediction in BC, compared to the traditional clinical biomarkers and molecular biomarkers before (8). Moreover, this signature was also significantly associated with the specific clinical features of BC, which would help the clinician in patients’ management and treatment selection. Ultimately, a more stable and reliable nomogram was established to predict the survival of BC patients.

As an intracellular organelle of eukaryotes, mitochondrion plays critical roles in cell metabolism (28). As is known, the structure and function of mitochondria are determined by mitochondrial-related genes in the cell nucleus and mitochondria. Current evidence has already demonstrated that germline mitochondrial DNA could predict the risk of bladder carcinogenesis, and their alterations were suggested as promising indicators for BC (29–31). Besides, the changes in the expression of mitochondrial genes involved in the mitochondrial electron respiratory chain were also implicated in solid tumors (32). Herein, we further explored the roles of the nuclear MTRGs in BC.

Some of the identified nuclear MTRGs have been revealed to be involved in tumor formation, progression, metastasis, and recurrence. NADH:ubiquinone oxidoreductase core subunit Fe-S protein 1 (NDUFS1) is very important to electron transfer, while altered *NDUFS1* expression would lead to the decrease of mitochondrial membrane potential, the elevated production of reactive oxygen species, and corresponding tumor progression, migration, and epithelial-mesenchymal transition (33). NDUFA1, as another component in mitochondrial NADH:ubiquinone oxidoreductase (complex I), is essential for respiratory activity (34). And the downregulation of *NDUFA1* was correlated with basal cell carcinoma (35). *COX7B*, provided cytochrome oxidase activity in cells, was demonstrated to serve as a platinum resistance biomarker in BC (36). In addition to the structural nuclear MTRGs, *ATAD3*, as a non-structural nuclear MTRG, has been used as the prognostic biomarker for hepatocellular carcinoma (37). The knockdown of glycyl-tRNA synthetase (*GARS*) could decrease the protein neddylation and cause the abnormal cell cycle (38), which were closely correlated with tumor initiation and invasiveness in the BC (39). The oncogene *IARS2* could prompt the tumorigenesis of non-small-cell lung cancer (40). And the aberrant expression of mitochondrial ribosomal protein S16 (*MRPS16*) could facilitate tumor cell growth, migration, and invasion *via* activating the PI3K/AKT signaling pathway (41). Moreover, some other non-structural nuclear MTRGs, such as *SUCLA2*, *DARS2*, and *TRMU*, can regulate tumor cells and influence the prognosis of cancer patients (42–44). Remarkably, most of the studies focused on one single MTRG or one related signaling pathway in tumor initiation, progression, metastasis, and its correlation with the prognosis of various cancers. In our work, the complex biological processes of mitochondria were spotlighted, and the utilization of a nuclear mitochondria-related gene set will be more credible and effective to identify the prognosis of BC.

Nowadays, the clinical staging, TNM staging, and histological grading are still the most commonly used tool for the prognosis prediction and treatment strategies of BC patients (45). However, the BC heterogeneity often made it hard for clinicians to improve the management of BC and make decisions for the treatments of BC patients (14). In the present study, the prognostic nomogram was developed with the advantage of overcoming the BC heterogeneity, which could cause the inaccuracies in the prognosis prediction of BC patients. Meanwhile, the high AUC value for OS at 1, 3, and 5 years indicated confirmatory evidence that the novel constructed nomogram was trustworthy. In clinical practices, it is challenging for clinicians to make decisions for clinical staging of high-grade prostate carcinoma and/or infiltrating urothelial carcinoma in tumor tissue specimens (46). The histopathological and clinical feature analyses in the present study demonstrated that no matter if patients were male or female, had any histological variants, presented with concomitant prostate cancer or not, the higher nuclear MTRGs score nearly always predicted the worse overall survival of BC. But we found that there were no any statistically significant differences in overall survival of the BC patients with high or low nuclear MTRGs score in three cohorts, probably because the number of female samples was a little small in these cohorts, which needed further verification. For the early-stage BC patients, they have great possibilities to develop to have the relapse of disease (47), because it is lack of the reliable and precise guidelines for the early-stage BC patients when the clinicians make the treatment strategies (48, 49). The nuclear MTRGs score was the most significant risk factor responsible for the prognosis prediction of BC patients, compared to the clinicopathological indexes. Thus, it was recommended that the nuclear MTRGs score could be employed to help the management of BC, especially for the early-stage BC patients. In addition, in the present study, the nuclear MTRGs score also exhibited its potential clinical application in distinguishing the NMIBC patients and MIBC patients, which could be helpful for the clinicians as well.

Enrichment Analyses of Hallmark gene set and KEGG pathway demonstrated significant differences in many biological processes, such as E2F targets, G2M checkpoint, cell cycle, etc., which are important to tumor progression and metastasis (50). The enrichment of infiltrated immune lymphocytes, especially CD8+ T cells, indicated that patients in the high nuclear MTRGs score group were more sensitive to immune checkpoint inhibitors (51). Moreover, some therapies for BC targeted mitochondrial dysfunction are developing. For instance, a previous study designed a hybrid peptide of Bld-1-KLA as a BC-targeted therapeutic agent, which could bind to BC tumor cells and disrupt mitochondrial membrane and induce the death of BC tumor cells (52). Simultaneously, more and more targeted therapies for mitochondrial DNA, metabolic enzymes, and related proteins have been proposed to improve the outcomes of BC patients (53). Therefore, according to the findings in this work, the identified 16 nuclear MTRGs could be applied to the development of targeted therapeutics in BC patients as well.

In total, we successfully developed a nuclear MTRGs signature with 16 nuclear MTRGs, including three structural and 13 non-structural nuclear MTRGs, which had significant influences on the prognosis of BC patients. The defined scoring system based on the nuclear MTRGs signature had the capacity of not only determining the clinic risk of BC patients but also differentiating the NMIBC and MIBC patients. However, there existed some limitations in our study, as all the findings need to be further verified in more independent cohorts and prospective samples. After all, the prognostic signature, the scoring system, and the predictive nomogram were constructed based on the public databases. Besides, there are actually a lot of histopathological subtypes of BC, such as adenocarcinoma, papillary (micropapillary) carcinoma, squamous cell carcinoma, sarcomatoid, etc. (54–57). Nevertheless, the classification of BC in the TCGA cohort was limited. We have been collecting BLCA patient samples in our hospital, which is necessary to further test the prognostic signature and nomogram. In addition, the analyses of ROC curves showed the better sensitivity and specificity of the nuclear MTRGs signature, compared with those of every single nuclear MTRG. But we did not take mtDNA into investigations together in this work, though an integrated nuclear MTRGs gene set was established to identify the survival of BC patients. The mitochondrial DNA was revealed to be correlated with the carcinogenesis of the bladder, so we would explore the potential ability of prediction prognosis model with both nuclear MTRGs and mtDNA included in the future.

## Conclusions

This is the first study identifying a nuclear MTRGs multigene signature and evaluating the integrated roles of nuclear MTRGs in the progression of BC patients. Moreover, a robust tool based on the expression profile of MTRGs involved in the signature was constructed for predicting the prognosis of BC patients. In addition, the analyses of clinical features and the histopathological characteristics further demonstrated the clinical applicability of the nuclear MTRGs signature and prognostic nomogram, which would help improve the BC management and contribute to the precision treatment of BC.

## Data Availability Statement

The original contributions presented in the study are included in the article/**Supplementary Material**. Further inquiries can be directed to the corresponding author.

## Author Contributions

XJ and BS proposed the conception and designed the study. XJ, YX, and HM majored significantly in the data analyses. XJ, YL, and JC wrote the main text of manuscript. XJ and HH processed the figures and tables in this work. HM, GY, and BS revised the manuscript. All authors contributed to the article and approved the submitted version.

## Funding

This study was funded by Taishan Scholar Fund (No. ts201511092) & National Natural Science Foundation of Chian (No. 81970661).

## Conflict of Interest

The authors declare that the research was conducted in the absence of any commercial or financial relationships that could be construed as a potential conflict of interest.

## Publisher’s Note

All claims expressed in this article are solely those of the authors and do not necessarily represent those of their affiliated organizations, or those of the publisher, the editors and the reviewers. Any product that may be evaluated in this article, or claim that may be made by its manufacturer, is not guaranteed or endorsed by the publisher.

## Supplementary Material

Supplementary Figure S1 Comparison of the mRNA expression data (only significantly differential expressed genes were shown, *p < 0.05) between BLCA patients and normal people samples from the TCGA cohort.

Supplementary Figure 2 cBioPortal oncoprints of the nuclear MTRGs expression profile **(A)**, the mutation profile **(B)**, and the copy number alteration profile **(C)** in each BLCA patient from the TCGA.

Supplementary Figure S3 Comparison of ROC curves for OS at 1-, 3-, 5-year between the nuclear MTRGs score and each single MTRG from the signature.

Supplementary Figure S4
**(A, B)** Comparison of the nuclear MTRGs score, and the overall survival between the BC patients presented with concomitant prostate cancer or not. **(C, D)** Survival analysis of the BC patients with high- or low- nuclear MTRGs score among the BC patients with concomitant prostate cancer or not.

Supplementary Figure S5 Survival analysis of the BC patients with high- or low- nuclear MTRGs score in the male and female groups.
