# Supplementary figures and images for: Identification of a Nuclear Mitochondrial-Related Multi-Genes Signature to Predict the Prognosis of Bladder Cancer
Source: Front Oncol. 2021 Oct 6;11:746029. doi: 10.3389/fonc.2021.746029 (PMC8528313; doi:10.3389/fonc.2021.746029)

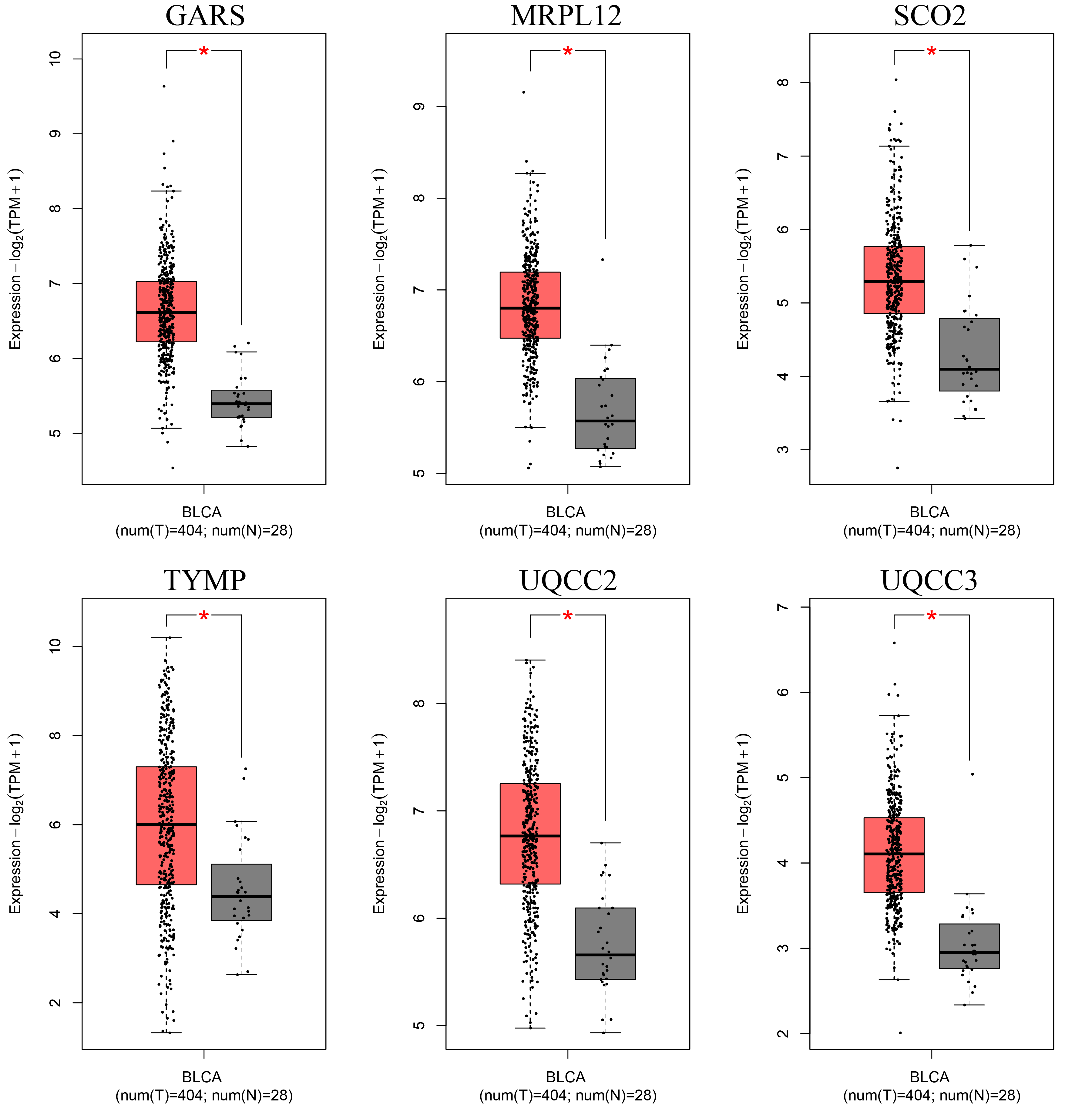

Supplement: Supplementary Figure S1 — Comparison of the mRNA expression data (only significantly differential expressed genes were shown, *p < 0.05) between BLCA patients and normal people samples from the TCGA cohort. [file Image_1.png]

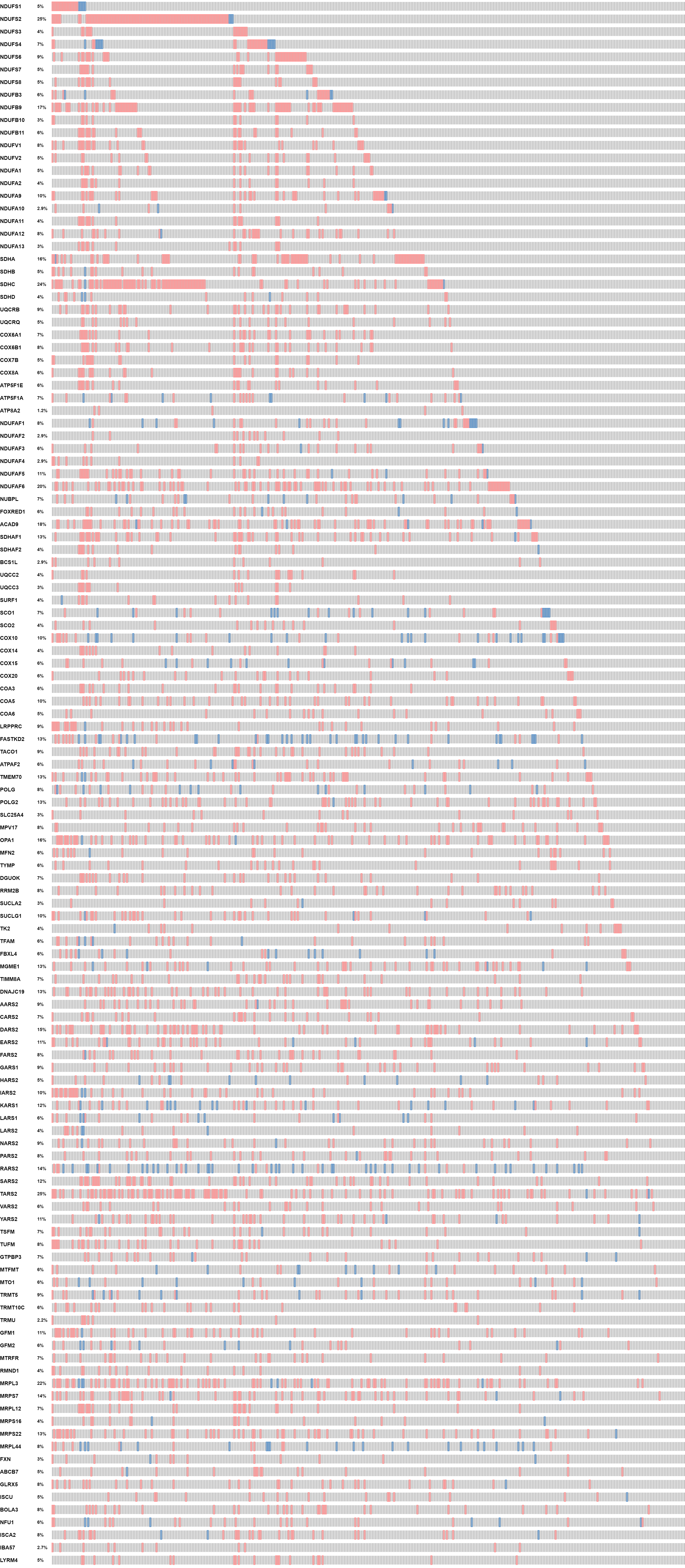

Supplement: Supplementary Figure 2 — cBioPortal oncoprints of the nuclear MTRGs expression profile (A), the mutation profile (B), and the copy number alteration profile (C) in each BLCA patient from the TCGA. [file Image_2.png]

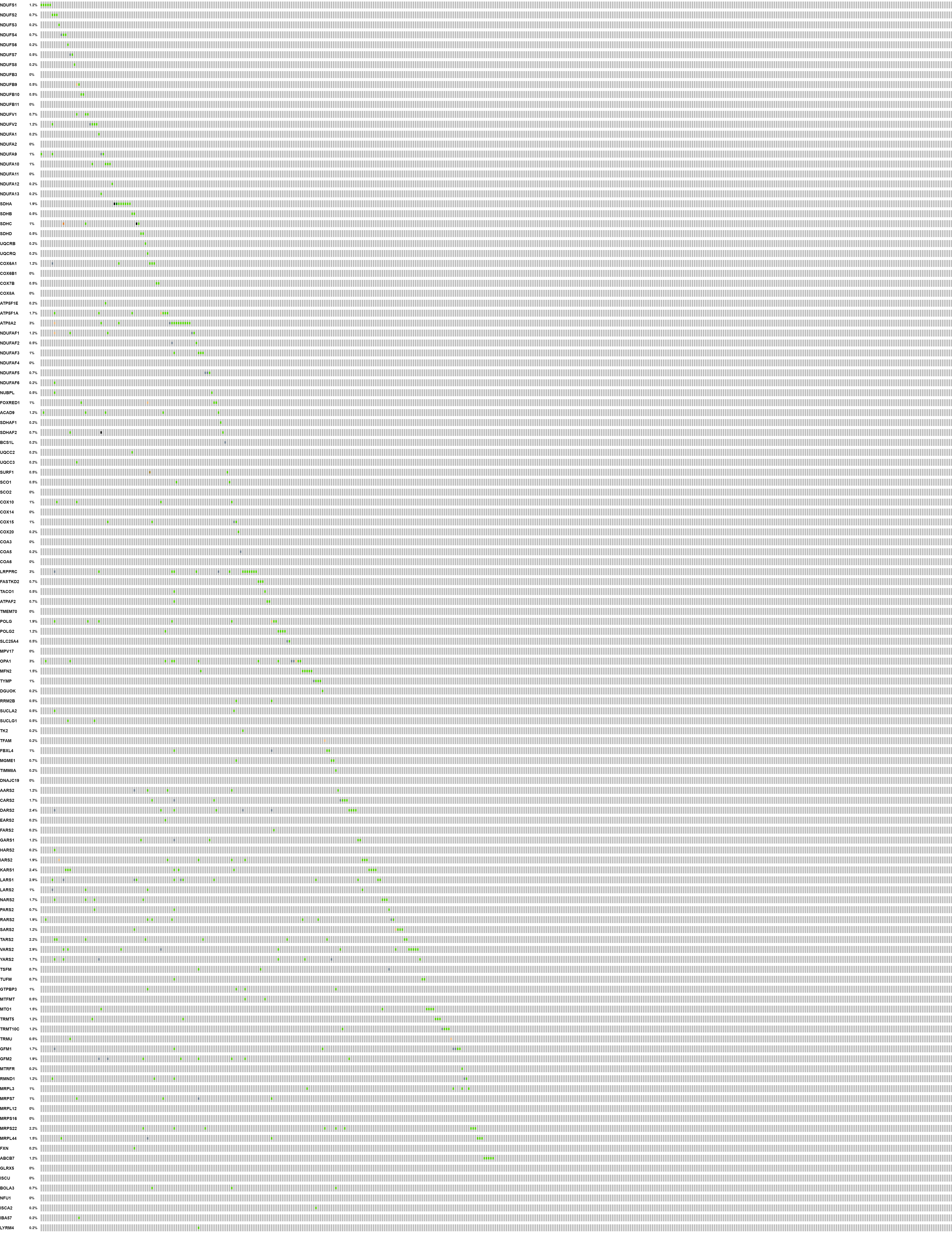

Supplement: Supplementary Figure S3 — Comparison of ROC curves for OS at 1-, 3-, 5-year between the nuclear MTRGs score and each single MTRG from the signature. [file Image_3.png]

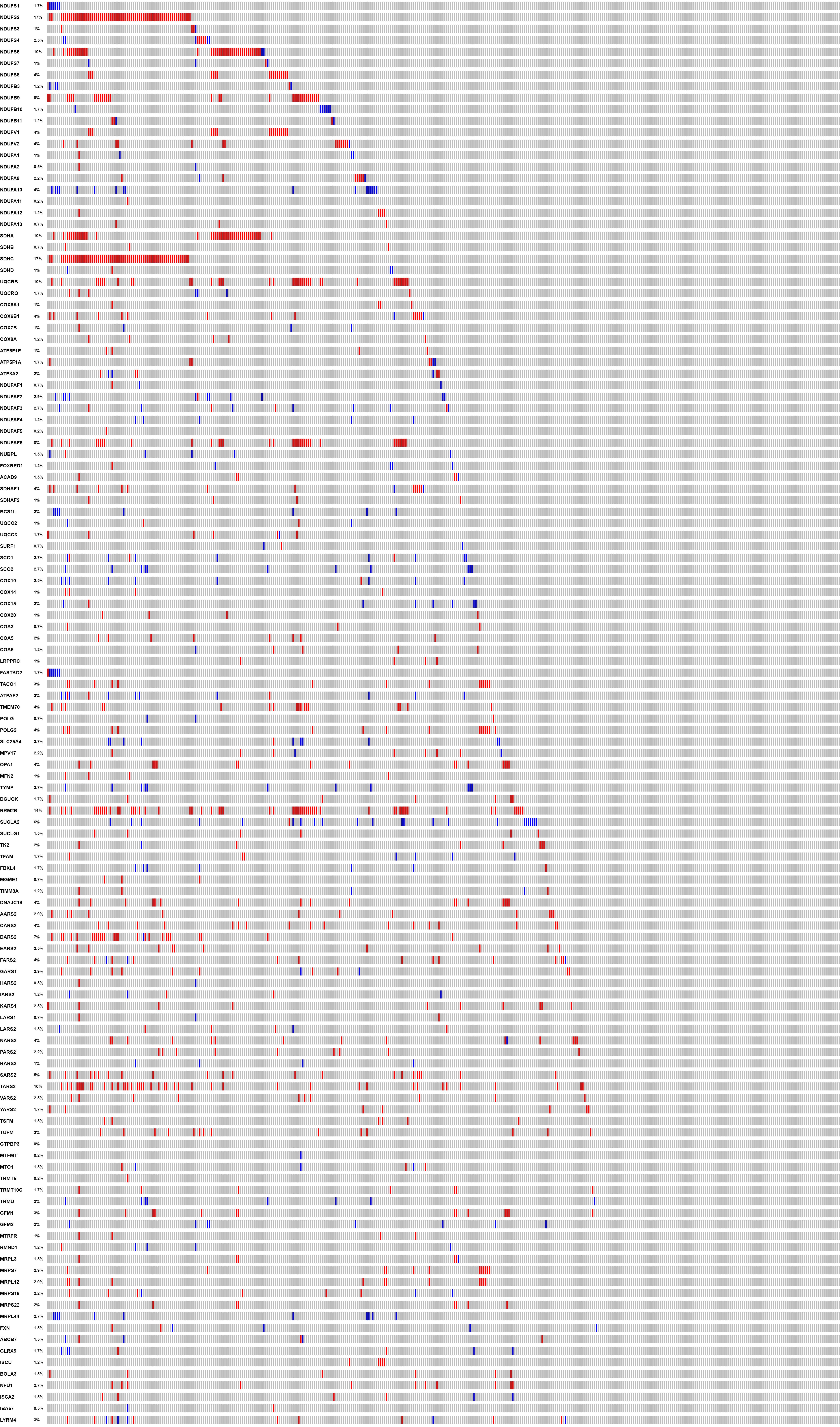

Supplement: Supplementary Figure S4 — (A, B) Comparison of the nuclear MTRGs score, and the overall survival between the BC patients presented with concomitant prostate cancer or not. (C, D) Survival analysis of the BC patients with high- or low- nuclear MTRGs score among the BC patients with concomitant prostate cancer or not. [file Image_4.png]

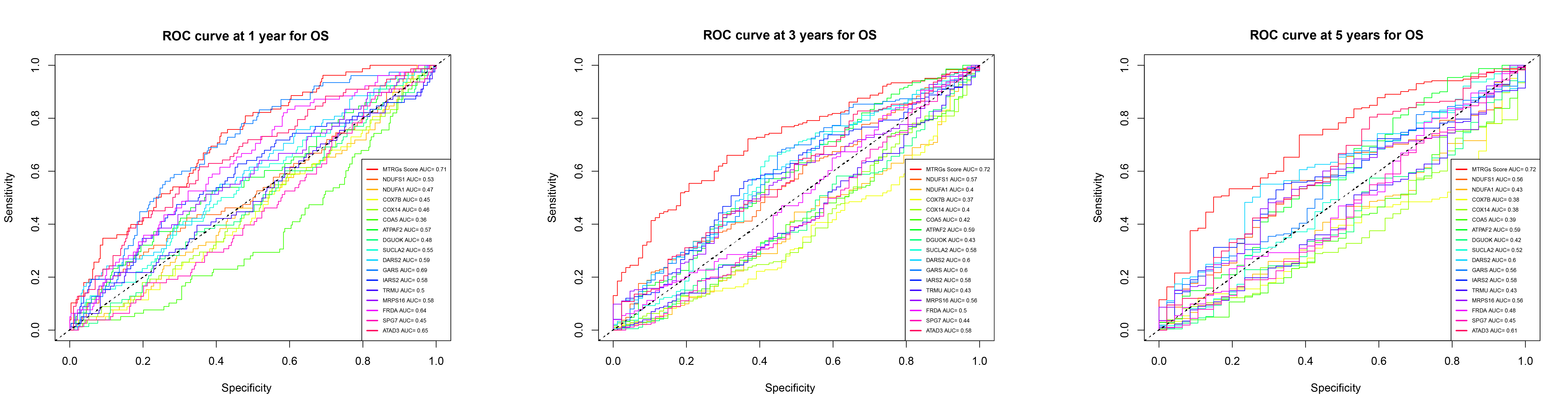

Supplement: Supplementary Figure S5 — Survival analysis of the BC patients with high- or low- nuclear MTRGs score in the male and female groups. [file Image_5.png]

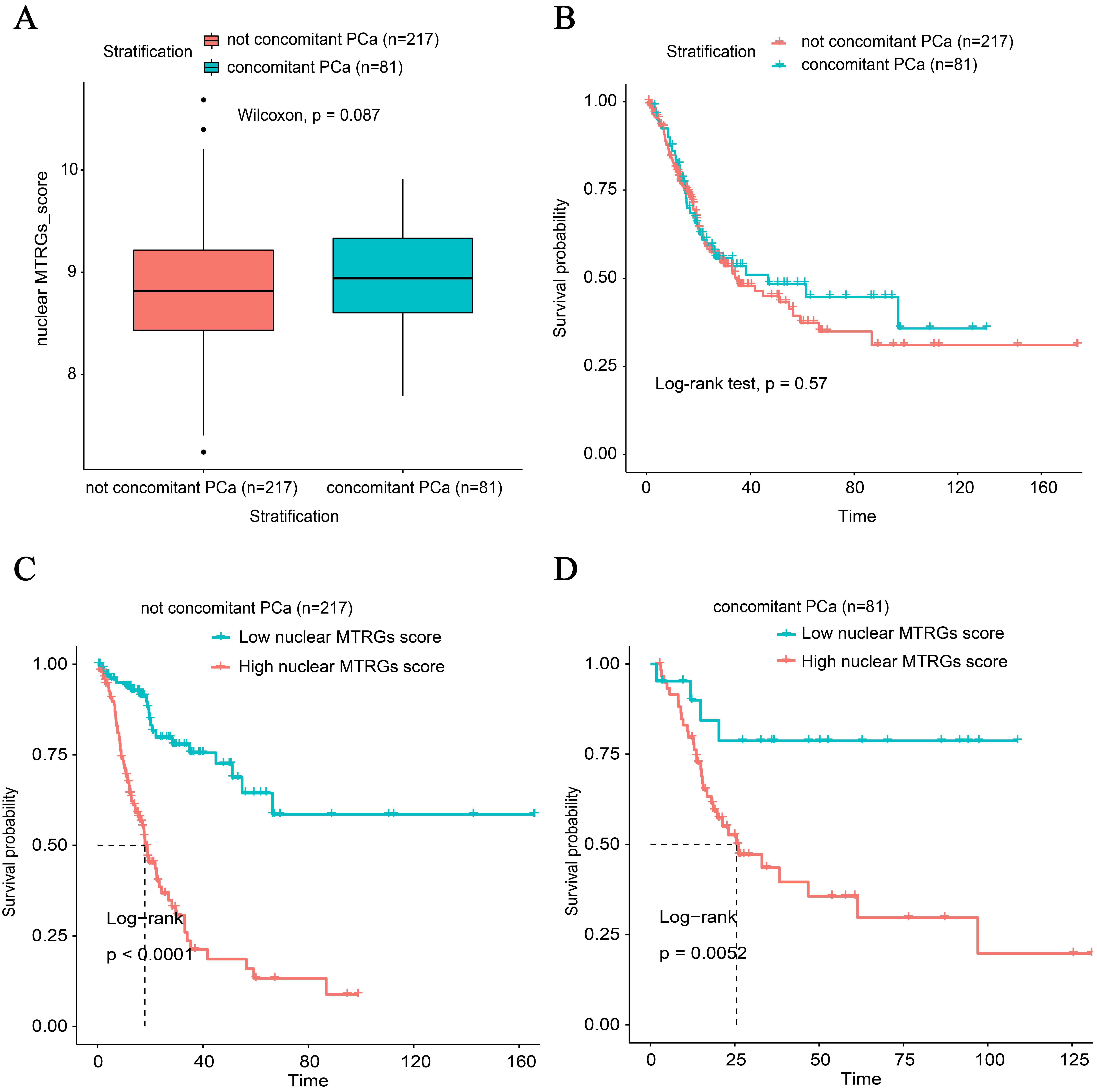

Supplement: Supplementary file 6 [file Image_6.png]
