# Supplementary material for: Identification of a Nuclear Mitochondrial-Related Multi-Genes Signature to Predict the Prognosis of Bladder Cancer
Source: Front Oncol. 2021 Oct 6;11:746029. doi: 10.3389/fonc.2021.746029 (PMC8528313; doi:10.3389/fonc.2021.746029)
Supplement: Supplementary file 8 [file Table_1.docx]

**Table S1.** The patient number of related mutated genes in the high- and low- nuclear MTRGs score group.

| **Gene** | **Patient Counts in high MTRGs score group (N)** | | **Patient Counts in low MTRGs score group (N)** | **Adjust p-value** |
| --- | --- | --- | --- | --- |
| *TP53* | | 119 | 75 | 0.068297 |
| *FGFR3* | | 14 | 42 | 0.091712 |
| *RB1* | | 49 | 21 | 0.4447 |
| *THBS2* | | 13 | 1 | 0.610267 |
| *CTNND1* | | 0 | 9 | 0.610267 |
| *KIF1B* | | 2 | 14 | 0.610267 |
| *POLE* | | 6 | 21 | 0.610267 |
| *CDH12* | | 12 | 1 | 0.610267 |
| *AP1B1* | | 1 | 11 | 0.610267 |
| *MCTP1* | | 1 | 11 | 0.610267 |
| *DENND4B* | | 2 | 13 | 0.610267 |
| *CNGB3* | | 0 | 8 | 0.610267 |
| *DAB2* | | 0 | 8 | 0.610267 |
| *OR6F1* | | 0 | 8 | 0.610267 |
| *SLCO4A1* | | 0 | 8 | 0.610267 |
| *DNAI2* | | 9 | 0 | 0.610267 |
| *MYH2* | | 3 | 15 | 0.610267 |
| *SETX* | | 6 | 20 | 0.610267 |
| *IGSF1* | | 1 | 10 | 0.610267 |
| *PCDHGA3* | | 1 | 10 | 0.610267 |
| *FAM83H* | | 3 | 14 | 0.610267 |
| *MIA3* | | 2 | 12 | 0.610267 |
| *NCOA3* | | 2 | 12 | 0.610267 |
| *AKAP11* | | 13 | 2 | 0.610267 |
| *DOCK8* | | 13 | 2 | 0.610267 |
| *AVL9* | | 0 | 7 | 0.610267 |
| *COL18A1* | | 0 | 7 | 0.610267 |
| *GNL2* | | 0 | 7 | 0.610267 |
| *MAN2B2* | | 0 | 7 | 0.610267 |
| *SETD4* | | 0 | 7 | 0.610267 |
| *ZNF716* | | 0 | 7 | 0.610267 |
| *ZNF283* | | 8 | 0 | 0.610267 |
| *MYCBP2* | | 10 | 25 | 0.610267 |
| *CASC1* | | 1 | 9 | 0.610267 |
| *IQGAP3* | | 1 | 9 | 0.610267 |
| *OBSL1* | | 1 | 9 | 0.610267 |
| *PRKCQ* | | 1 | 9 | 0.610267 |
| *DNAH6* | | 3 | 13 | 0.610267 |
| *KIF14* | | 3 | 13 | 0.610267 |
| *ADCY5* | | 2 | 11 | 0.610267 |
| *ANKS1A* | | 2 | 11 | 0.610267 |
| *GYS2* | | 2 | 11 | 0.610267 |
| *MLLT4* | | 2 | 11 | 0.610267 |
| *OVCH1* | | 2 | 11 | 0.610267 |
| *TRPA1* | | 2 | 11 | 0.610267 |
| *KANSL1* | | 6 | 18 | 0.610267 |
| *ALMS1* | | 10 | 24 | 0.610267 |
| *AGR3* | | 0 | 6 | 0.610267 |
| *AMPD2* | | 0 | 6 | 0.610267 |
| *ARHGEF3* | | 0 | 6 | 0.610267 |
| *CEP55* | | 0 | 6 | 0.610267 |
| *CPNE3* | | 0 | 6 | 0.610267 |
| *DENND5A* | | 0 | 6 | 0.610267 |
| *FSD2* | | 0 | 6 | 0.610267 |
| *IGF2BP3* | | 0 | 6 | 0.610267 |
| *IKBIP* | | 0 | 6 | 0.610267 |
| *KAT8* | | 0 | 6 | 0.610267 |
| *KRT20* | | 0 | 6 | 0.610267 |
| *LEPRE1* | | 0 | 6 | 0.610267 |
| *MDH1B* | | 0 | 6 | 0.610267 |
| *MKLN1* | | 0 | 6 | 0.610267 |
| *MRPS31* | | 0 | 6 | 0.610267 |
| *MRVI1* | | 0 | 6 | 0.610267 |
| *MYBL1* | | 0 | 6 | 0.610267 |
| *NMT2* | | 0 | 6 | 0.610267 |
| *PRPF19* | | 0 | 6 | 0.610267 |
| *RARS* | | 0 | 6 | 0.610267 |
| *SLC39A6* | | 0 | 6 | 0.610267 |
| *SLC6A12* | | 0 | 6 | 0.610267 |
| *UBC* | | 0 | 6 | 0.610267 |
| *FUT8* | | 7 | 0 | 0.610267 |
| *KDM4A* | | 7 | 0 | 0.610267 |
| *L3MBTL3* | | 7 | 0 | 0.610267 |
| *METTL12* | | 7 | 0 | 0.610267 |
| *PCNXL3* | | 7 | 0 | 0.610267 |
| *SAMD11* | | 7 | 0 | 0.610267 |
| *ATAD5* | | 9 | 22 | 0.610267 |
| *HEG1* | | 3 | 12 | 0.610267 |
| *MIOS* | | 3 | 12 | 0.610267 |
| *AP3D1* | | 1 | 8 | 0.610267 |
| *C2orf44* | | 1 | 8 | 0.610267 |
| *CDC42BPB* | | 1 | 8 | 0.610267 |
| *DPP4* | | 1 | 8 | 0.610267 |
| *ETV1* | | 1 | 8 | 0.610267 |
| *KCNS2* | | 1 | 8 | 0.610267 |
| *KIAA1199* | | 1 | 8 | 0.610267 |
| *MGRN1* | | 1 | 8 | 0.610267 |
| *NOL6* | | 1 | 8 | 0.610267 |
| *PCDHGA8* | | 1 | 8 | 0.610267 |
| *PPIG* | | 1 | 8 | 0.610267 |
| *SP1* | | 1 | 8 | 0.610267 |
| *TYRO3* | | 1 | 8 | 0.610267 |
| *ZBTB11* | | 1 | 8 | 0.610267 |
| *KIF23* | | 9 | 1 | 0.610267 |
| *MUC17* | | 16 | 31 | 0.610267 |
| *FLNA* | | 11 | 2 | 0.610267 |
| *BNC2* | | 2 | 10 | 0.610267 |
| *RALGAPA1* | | 2 | 10 | 0.610267 |
| *SEMA3E* | | 2 | 10 | 0.610267 |
| *SLC8A2* | | 2 | 10 | 0.610267 |
| *SPAG5* | | 2 | 10 | 0.610267 |
| *MYO1F* | | 5 | 15 | 0.610267 |
| *OTOF* | | 5 | 15 | 0.610267 |
| *FBN2* | | 9 | 21 | 0.610267 |
| *ITGA4* | | 4 | 13 | 0.610267 |
| *SHANK1* | | 4 | 13 | 0.610267 |
| *TANC1* | | 4 | 13 | 0.610267 |
| *NAV2* | | 14 | 4 | 0.610267 |
| *AHR* | | 6 | 16 | 0.610267 |
| *UNC13C* | | 6 | 16 | 0.610267 |
| *ABCG1* | | 0 | 5 | 0.610267 |
| *ACAD9* | | 0 | 5 | 0.610267 |
| *ADC* | | 0 | 5 | 0.610267 |
| *ARSG* | | 0 | 5 | 0.610267 |
| *ASNA1* | | 0 | 5 | 0.610267 |
| *BBS12* | | 0 | 5 | 0.610267 |
| *C17orf47* | | 0 | 5 | 0.610267 |
| *CCDC155* | | 0 | 5 | 0.610267 |
| *CCDC37* | | 0 | 5 | 0.610267 |
| *CDCA7* | | 0 | 5 | 0.610267 |
| *CDYL* | | 0 | 5 | 0.610267 |
| *CNGA3* | | 0 | 5 | 0.610267 |
| *CPT2* | | 0 | 5 | 0.610267 |
| *DDX50* | | 0 | 5 | 0.610267 |
| *DUOXA2* | | 0 | 5 | 0.610267 |
| *EDEM2* | | 0 | 5 | 0.610267 |
| *ELMO3* | | 0 | 5 | 0.610267 |
| *EXOC5* | | 0 | 5 | 0.610267 |
| *FCRL5* | | 0 | 5 | 0.610267 |
| *FTH1* | | 0 | 5 | 0.610267 |
| *FXR2* | | 0 | 5 | 0.610267 |
| *GFRAL* | | 0 | 5 | 0.610267 |
| *HIPK2* | | 0 | 5 | 0.610267 |
| *IRF3* | | 0 | 5 | 0.610267 |
| *JPH3* | | 0 | 5 | 0.610267 |
| *KCNJ14* | | 0 | 5 | 0.610267 |
| *KDM5D* | | 0 | 5 | 0.610267 |
| *KRT10* | | 0 | 5 | 0.610267 |
| *MAP2K2* | | 0 | 5 | 0.610267 |
| *MAZ* | | 0 | 5 | 0.610267 |
| *MROH7-TTC4* | | 0 | 5 | 0.610267 |
| *MYF6* | | 0 | 5 | 0.610267 |
| *NDUFS1* | | 0 | 5 | 0.610267 |
| *NKAIN2* | | 0 | 5 | 0.610267 |
| *OSBPL6* | | 0 | 5 | 0.610267 |
| *PAQR8* | | 0 | 5 | 0.610267 |
| *PHF11* | | 0 | 5 | 0.610267 |
| *PJA1* | | 0 | 5 | 0.610267 |
| *PPP2R5B* | | 0 | 5 | 0.610267 |
| *PRKAR2B* | | 0 | 5 | 0.610267 |
| *RNF145* | | 0 | 5 | 0.610267 |
| *SCAMP3* | | 0 | 5 | 0.610267 |
| *SH3GL3* | | 0 | 5 | 0.610267 |
| *SH3RF2* | | 0 | 5 | 0.610267 |
| *SHROOM1* | | 0 | 5 | 0.610267 |
| *SLC13A4* | | 0 | 5 | 0.610267 |
| *SLC15A2* | | 0 | 5 | 0.610267 |
| *SLC16A6* | | 0 | 5 | 0.610267 |
| *SOHLH2* | | 0 | 5 | 0.610267 |
| *STEAP2* | | 0 | 5 | 0.610267 |
| *TAS2R60* | | 0 | 5 | 0.610267 |
| *TBX2* | | 0 | 5 | 0.610267 |
| *TRPV3* | | 0 | 5 | 0.610267 |
| *TUBB3* | | 0 | 5 | 0.610267 |
| *UBXN4* | | 0 | 5 | 0.610267 |
| *USP49* | | 0 | 5 | 0.610267 |
| *WDR41* | | 0 | 5 | 0.610267 |
| *YIPF1* | | 0 | 5 | 0.610267 |
| *ZBTB33* | | 0 | 5 | 0.610267 |
| *ZC3H3* | | 0 | 5 | 0.610267 |
| *ZNF551* | | 0 | 5 | 0.610267 |
| *CADM1* | | 6 | 0 | 0.610267 |
| *CHPT1* | | 6 | 0 | 0.610267 |
| *CPSF6* | | 6 | 0 | 0.610267 |
| *KDM6B* | | 6 | 0 | 0.610267 |
| *KSR1* | | 6 | 0 | 0.610267 |
| *MORC4* | | 6 | 0 | 0.610267 |
| *NCOA4* | | 6 | 0 | 0.610267 |
| *RBP2* | | 6 | 0 | 0.610267 |
| *SBF2* | | 3 | 11 | 0.610267 |
| *STON1* | | 3 | 11 | 0.610267 |
| *TNRC18* | | 3 | 11 | 0.610267 |
| *TRPM2* | | 3 | 11 | 0.610267 |
| *CCDC47* | | 2 | 9 | 0.610267 |
| *DENND2A* | | 2 | 9 | 0.610267 |
| *EFHB* | | 2 | 9 | 0.610267 |
| *HORMAD1* | | 2 | 9 | 0.610267 |
| *MPEG1* | | 2 | 9 | 0.610267 |
| *MYRF* | | 2 | 9 | 0.610267 |
| *NEO1* | | 2 | 9 | 0.610267 |
| *NOC3L* | | 2 | 9 | 0.610267 |
| *RPGR* | | 2 | 9 | 0.610267 |
| *TBCD* | | 2 | 9 | 0.610267 |
| *ZBTB21* | | 2 | 9 | 0.610267 |
| *ZNF502* | | 2 | 9 | 0.610267 |
| *KCNT1* | | 10 | 2 | 0.610267 |
| *UGGT1* | | 10 | 2 | 0.610267 |
| *SOS1* | | 5 | 14 | 0.610267 |
| *ADAM15* | | 1 | 7 | 0.610267 |
| *ANKRD28* | | 1 | 7 | 0.610267 |
| *ARHGAP11A* | | 1 | 7 | 0.610267 |
| *ATE1* | | 1 | 7 | 0.610267 |
| *ATG4D* | | 1 | 7 | 0.610267 |
| *CCDC88B* | | 1 | 7 | 0.610267 |
| *CD96* | | 1 | 7 | 0.610267 |
| *CDCA7L* | | 1 | 7 | 0.610267 |
| *CLIP4* | | 1 | 7 | 0.610267 |
| *EHMT1* | | 1 | 7 | 0.610267 |
| *EIF2B5* | | 1 | 7 | 0.610267 |
| *FAM129A* | | 1 | 7 | 0.610267 |
| *HCN4* | | 1 | 7 | 0.610267 |
| *ICA1* | | 1 | 7 | 0.610267 |
| *ITGA5* | | 1 | 7 | 0.610267 |
| *KCNJ6* | | 1 | 7 | 0.610267 |
| *MDM1* | | 1 | 7 | 0.610267 |
| *MFSD11* | | 1 | 7 | 0.610267 |
| *NCOA5* | | 1 | 7 | 0.610267 |
| *NOL9* | | 1 | 7 | 0.610267 |
| *NRAS* | | 1 | 7 | 0.610267 |
| *NRBP1* | | 1 | 7 | 0.610267 |
| *OR13C3* | | 1 | 7 | 0.610267 |
| *OR2T12* | | 1 | 7 | 0.610267 |
| *PCDHB15* | | 1 | 7 | 0.610267 |
| *RIN3* | | 1 | 7 | 0.610267 |
| *SCARF2* | | 1 | 7 | 0.610267 |
| *SNRPB* | | 1 | 7 | 0.610267 |
| *TAF15* | | 1 | 7 | 0.610267 |
| *TBC1D9* | | 1 | 7 | 0.610267 |
| *THOC2* | | 1 | 7 | 0.610267 |
| *TUBA1A* | | 1 | 7 | 0.610267 |
| *WDR44* | | 1 | 7 | 0.610267 |
| *ZNF813* | | 1 | 7 | 0.610267 |
| *ABL1* | | 8 | 1 | 0.610267 |
| *DLL1* | | 8 | 1 | 0.610267 |
| *HSPA4L* | | 8 | 1 | 0.610267 |
| *PLCH2* | | 8 | 1 | 0.610267 |
| *SLC26A9* | | 8 | 1 | 0.610267 |
| *ADAMTS9* | | 18 | 7 | 0.610267 |
| *FMN2* | | 18 | 7 | 0.610267 |
| *NFE2L2* | | 18 | 7 | 0.610267 |
| *ADAMTS16* | | 13 | 4 | 0.654086 |
| *ARFGEF2* | | 4 | 12 | 0.654086 |
| *ASTN1* | | 4 | 12 | 0.654086 |
| *PTPRB* | | 4 | 12 | 0.654086 |
| *ROBO1* | | 4 | 12 | 0.654086 |
| *SMG1* | | 6 | 15 | 0.654086 |
| *BSN* | | 9 | 19 | 0.654086 |
| *CDH17* | | 3 | 10 | 0.654086 |
| *CEP104* | | 3 | 10 | 0.654086 |
| *EPHB1* | | 3 | 10 | 0.654086 |
| *FGD6* | | 3 | 10 | 0.654086 |
| *LRRC37B* | | 3 | 10 | 0.654086 |
| *MAP4K3* | | 3 | 10 | 0.654086 |
| *MBD5* | | 3 | 10 | 0.654086 |
| *MYOM2* | | 3 | 10 | 0.654086 |
| *PPFIA1* | | 3 | 10 | 0.654086 |
| *SORCS3* | | 3 | 10 | 0.654086 |
| *KDM6A* | | 44 | 61 | 0.654086 |
| *SMARCA2* | | 7 | 16 | 0.654086 |
| *APC* | | 17 | 7 | 0.654086 |
| *CUL1* | | 14 | 5 | 0.654086 |
| *FNBP4* | | 5 | 13 | 0.654086 |
| *TP53BP2* | | 5 | 13 | 0.654086 |
| *FBXW7* | | 21 | 10 | 0.654086 |
| *ARRB1* | | 5 | 0 | 0.654086 |
| *CHST8* | | 5 | 0 | 0.654086 |
| *CREB3L2* | | 5 | 0 | 0.654086 |
| *CRTAM* | | 5 | 0 | 0.654086 |
| *DCP1A* | | 5 | 0 | 0.654086 |
| *DZANK1* | | 5 | 0 | 0.654086 |
| *ERC1* | | 5 | 0 | 0.654086 |
| *FAM169A* | | 5 | 0 | 0.654086 |
| *FAM170A* | | 5 | 0 | 0.654086 |
| *GPLD1* | | 5 | 0 | 0.654086 |
| *GTF2F1* | | 5 | 0 | 0.654086 |
| *ITPKC* | | 5 | 0 | 0.654086 |
| *MTF2* | | 5 | 0 | 0.654086 |
| *NAGPA* | | 5 | 0 | 0.654086 |
| *OR13C9* | | 5 | 0 | 0.654086 |
| *OR51D1* | | 5 | 0 | 0.654086 |
| *PAPOLG* | | 5 | 0 | 0.654086 |
| *PARD6A* | | 5 | 0 | 0.654086 |
| *10-Sep* | | 5 | 0 | 0.654086 |
| *STAM* | | 5 | 0 | 0.654086 |
| *TMEM5* | | 5 | 0 | 0.654086 |
| *TXNRD1* | | 5 | 0 | 0.654086 |
| *ZBTB3* | | 5 | 0 | 0.654086 |
| *ACLY* | | 9 | 2 | 0.654086 |
| *DDX11* | | 9 | 2 | 0.654086 |
| *IBTK* | | 9 | 2 | 0.654086 |
| *IQSEC3* | | 9 | 2 | 0.654086 |
| *PAXBP1* | | 9 | 2 | 0.654086 |
| *PRDM9* | | 9 | 2 | 0.654086 |
| *SEC31B* | | 9 | 2 | 0.654086 |
| *SMARCC1* | | 9 | 2 | 0.654086 |
| *ACOX2* | | 2 | 8 | 0.654086 |
| *ACTN2* | | 2 | 8 | 0.654086 |
| *BEND3* | | 2 | 8 | 0.654086 |
| *CATSPERG* | | 2 | 8 | 0.654086 |
| *DROSHA* | | 2 | 8 | 0.654086 |
| *HSP90AB1* | | 2 | 8 | 0.654086 |
| *LDLR* | | 2 | 8 | 0.654086 |
| *MASTL* | | 2 | 8 | 0.654086 |
| *MEFV* | | 2 | 8 | 0.654086 |
| *MIER1* | | 2 | 8 | 0.654086 |
| *NOM1* | | 2 | 8 | 0.654086 |
| *OR6N1* | | 2 | 8 | 0.654086 |
| *PIGS* | | 2 | 8 | 0.654086 |
| *SGOL2* | | 2 | 8 | 0.654086 |
| *SMC6* | | 2 | 8 | 0.654086 |
| *TGFBRAP1* | | 2 | 8 | 0.654086 |
| *TMTC2* | | 2 | 8 | 0.654086 |
| *UNC13D* | | 2 | 8 | 0.654086 |
| *SZT2* | | 8 | 17 | 0.654086 |
| *WNK1* | | 8 | 17 | 0.654086 |
| *ELF3* | | 18 | 30 | 0.654086 |
| *ZFHX4* | | 18 | 30 | 0.654086 |
| *BCO2* | | 1 | 6 | 0.654086 |
| *C12orf4* | | 1 | 6 | 0.654086 |
| *CASC4* | | 1 | 6 | 0.654086 |
| *CCDC87* | | 1 | 6 | 0.654086 |
| *CCNL2* | | 1 | 6 | 0.654086 |
| *CDC42BPG* | | 1 | 6 | 0.654086 |
| *CDK11A* | | 1 | 6 | 0.654086 |
| *CDK9* | | 1 | 6 | 0.654086 |
| *CEP76* | | 1 | 6 | 0.654086 |
| *CERKL* | | 1 | 6 | 0.654086 |
| *CLSTN3* | | 1 | 6 | 0.654086 |
| *COL28A1* | | 1 | 6 | 0.654086 |
| *CYLC1* | | 1 | 6 | 0.654086 |
| *DCLRE1C* | | 1 | 6 | 0.654086 |
| *DHX37* | | 1 | 6 | 0.654086 |
| *EIF5* | | 1 | 6 | 0.654086 |
| *ERCC6L* | | 1 | 6 | 0.654086 |
| *ESRP2* | | 1 | 6 | 0.654086 |
| *FAM175B* | | 1 | 6 | 0.654086 |
| *FOXJ2* | | 1 | 6 | 0.654086 |
| *GLDC* | | 1 | 6 | 0.654086 |
| *IKZF3* | | 1 | 6 | 0.654086 |
| *IQCG* | | 1 | 6 | 0.654086 |
| *ITGA7* | | 1 | 6 | 0.654086 |
| *KBTBD3* | | 1 | 6 | 0.654086 |
| *LONRF2* | | 1 | 6 | 0.654086 |
| *LSM14B* | | 1 | 6 | 0.654086 |
| *LTBP3* | | 1 | 6 | 0.654086 |
| *MARK4* | | 1 | 6 | 0.654086 |
| *MOAP1* | | 1 | 6 | 0.654086 |
| *MTR* | | 1 | 6 | 0.654086 |
| *NBEAL2* | | 1 | 6 | 0.654086 |
| *NEDD9* | | 1 | 6 | 0.654086 |
| *NEK5* | | 1 | 6 | 0.654086 |
| *OR10J5* | | 1 | 6 | 0.654086 |
| *OR4M1* | | 1 | 6 | 0.654086 |
| *PARL* | | 1 | 6 | 0.654086 |
| *POGLUT1* | | 1 | 6 | 0.654086 |
| *POMGNT1* | | 1 | 6 | 0.654086 |
| *PPP1R15B* | | 1 | 6 | 0.654086 |
| *PPP1R18* | | 1 | 6 | 0.654086 |
| *PUS10* | | 1 | 6 | 0.654086 |
| *PXK* | | 1 | 6 | 0.654086 |
| *RHOBTB3* | | 1 | 6 | 0.654086 |
| *RNF112* | | 1 | 6 | 0.654086 |
| *SLMAP* | | 1 | 6 | 0.654086 |
| *SNRPA* | | 1 | 6 | 0.654086 |
| *SNTG1* | | 1 | 6 | 0.654086 |
| *SNX25* | | 1 | 6 | 0.654086 |
| *SRBD1* | | 1 | 6 | 0.654086 |
| *SRSF1* | | 1 | 6 | 0.654086 |
| *TCF4* | | 1 | 6 | 0.654086 |
| *TIMM44* | | 1 | 6 | 0.654086 |
| *TMCC2* | | 1 | 6 | 0.654086 |
| *TNFAIP6* | | 1 | 6 | 0.654086 |
| *TXLNB* | | 1 | 6 | 0.654086 |
| *UGT2B15* | | 1 | 6 | 0.654086 |
| *VAV1* | | 1 | 6 | 0.654086 |
| *WBP11* | | 1 | 6 | 0.654086 |
| *WDR93* | | 1 | 6 | 0.654086 |
| *ZFP69B* | | 1 | 6 | 0.654086 |
| *ZNF155* | | 1 | 6 | 0.654086 |
| *ZNF285* | | 1 | 6 | 0.654086 |
| *ZNF440* | | 1 | 6 | 0.654086 |
| *ZNF443* | | 1 | 6 | 0.654086 |
| *ZNF473* | | 1 | 6 | 0.654086 |
| *ZNF57* | | 1 | 6 | 0.654086 |
| *ZNF587* | | 1 | 6 | 0.654086 |
| *ZNF624* | | 1 | 6 | 0.654086 |
| *ZSWIM1* | | 1 | 6 | 0.654086 |
| *ABHD16A* | | 7 | 1 | 0.654086 |
| *ATP8B3* | | 7 | 1 | 0.654086 |
| *B4GALNT2* | | 7 | 1 | 0.654086 |
| *CLPTM1* | | 7 | 1 | 0.654086 |
| *EVC* | | 7 | 1 | 0.654086 |
| *KIAA0586* | | 7 | 1 | 0.654086 |
| *NFX1* | | 7 | 1 | 0.654086 |
| *PLXDC2* | | 7 | 1 | 0.654086 |
| *PPFIA4* | | 7 | 1 | 0.654086 |
| *PTPN14* | | 7 | 1 | 0.654086 |
| *RNF168* | | 7 | 1 | 0.654086 |
| *SLC30A6* | | 7 | 1 | 0.654086 |
| *TMEM145* | | 7 | 1 | 0.654086 |
| *VGF* | | 7 | 1 | 0.654086 |
| *ZC3H12A* | | 7 | 1 | 0.654086 |
| *ZNF12* | | 7 | 1 | 0.654086 |
| *EPHA3* | | 6 | 14 | 0.654086 |
| *MEGF8* | | 6 | 14 | 0.654086 |
| *UNC79* | | 6 | 14 | 0.654086 |
| *ANKAR* | | 4 | 11 | 0.654086 |
| *RAPGEF2* | | 4 | 11 | 0.654086 |
| *AFF4* | | 12 | 4 | 0.654086 |
| *BAI3* | | 12 | 4 | 0.654086 |
| *MYH1* | | 12 | 4 | 0.654086 |
| *PRDM16* | | 12 | 4 | 0.654086 |
| *UTRN* | | 12 | 22 | 0.654086 |
| *VPS13D* | | 12 | 22 | 0.654086 |
| *DNAH17* | | 23 | 12 | 0.654086 |
| *VCAN* | | 23 | 12 | 0.654086 |
| *DCC* | | 9 | 18 | 0.654086 |
| *MYO9A* | | 9 | 18 | 0.654086 |
| *TET1* | | 7 | 15 | 0.654086 |
| *SCN1A* | | 10 | 19 | 0.654086 |
| *C4orf21* | | 10 | 3 | 0.654086 |
| *NUP98* | | 10 | 3 | 0.654086 |
| *TAS1R3* | | 10 | 3 | 0.654086 |
| *UHRF1BP1* | | 10 | 3 | 0.654086 |
| *ABCC4* | | 3 | 9 | 0.654086 |
| *ANKLE2* | | 3 | 9 | 0.654086 |
| *CARF* | | 3 | 9 | 0.654086 |
| *H6PD* | | 3 | 9 | 0.654086 |
| *LCMT2* | | 3 | 9 | 0.654086 |
| *LRPPRC* | | 3 | 9 | 0.654086 |
| *NCKAP1* | | 3 | 9 | 0.654086 |
| *RBM47* | | 3 | 9 | 0.654086 |
| *SCN11A* | | 3 | 9 | 0.654086 |
| *SEC23IP* | | 3 | 9 | 0.654086 |
| *ZNF845* | | 3 | 9 | 0.654086 |
| *METTL3* | | 5 | 12 | 0.654086 |
| *NIPBL* | | 5 | 12 | 0.654086 |
| *PARP4* | | 5 | 12 | 0.654086 |
| *PXDN* | | 5 | 12 | 0.654086 |
| *AHNAK2* | | 19 | 30 | 0.654086 |
| *MYO3A* | | 8 | 16 | 0.654086 |
| *NRXN1* | | 8 | 16 | 0.654086 |
| *EP300* | | 38 | 25 | 0.654086 |
| *ABCD2* | | 2 | 7 | 0.654086 |
| *ANKRD55* | | 2 | 7 | 0.654086 |
| *ARHGAP12* | | 2 | 7 | 0.654086 |
| *ASTN2* | | 2 | 7 | 0.654086 |
| *ATMIN* | | 2 | 7 | 0.654086 |
| *ATP1A1* | | 2 | 7 | 0.654086 |
| *BLNK* | | 2 | 7 | 0.654086 |
| *CALCR* | | 2 | 7 | 0.654086 |
| *CNTN2* | | 2 | 7 | 0.654086 |
| *DNMT3A* | | 2 | 7 | 0.654086 |
| *DYRK1A* | | 2 | 7 | 0.654086 |
| *ERBB2IP* | | 2 | 7 | 0.654086 |
| *EXOC1* | | 2 | 7 | 0.654086 |
| *FAM214A* | | 2 | 7 | 0.654086 |
| *FAP* | | 2 | 7 | 0.654086 |
| *FGD5* | | 2 | 7 | 0.654086 |
| *GLTSCR1* | | 2 | 7 | 0.654086 |
| *GPR141* | | 2 | 7 | 0.654086 |
| *HTR1A* | | 2 | 7 | 0.654086 |
| *INTS8* | | 2 | 7 | 0.654086 |
| *IQGAP2* | | 2 | 7 | 0.654086 |
| *KCNJ2* | | 2 | 7 | 0.654086 |
| *KIF4B* | | 2 | 7 | 0.654086 |
| *LRRFIP2* | | 2 | 7 | 0.654086 |
| *LY9* | | 2 | 7 | 0.654086 |
| *MACC1* | | 2 | 7 | 0.654086 |
| *MAP10* | | 2 | 7 | 0.654086 |
| *MARK1* | | 2 | 7 | 0.654086 |
| *MTA2* | | 2 | 7 | 0.654086 |
| *MYH14* | | 2 | 7 | 0.654086 |
| *NCKAP1L* | | 2 | 7 | 0.654086 |
| *NCSTN* | | 2 | 7 | 0.654086 |
| *NOC2L* | | 2 | 7 | 0.654086 |
| *OFD1* | | 2 | 7 | 0.654086 |
| *PCDHGB6* | | 2 | 7 | 0.654086 |
| *PFAS* | | 2 | 7 | 0.654086 |
| *PNPT1* | | 2 | 7 | 0.654086 |
| *PPCS* | | 2 | 7 | 0.654086 |
| *SCYL3* | | 2 | 7 | 0.654086 |
| *SEL1L2* | | 2 | 7 | 0.654086 |
| *SEMA3C* | | 2 | 7 | 0.654086 |
| *SGIP1* | | 2 | 7 | 0.654086 |
| *SLC9C2* | | 2 | 7 | 0.654086 |
| *SPTB* | | 2 | 7 | 0.654086 |
| *SPTLC3* | | 2 | 7 | 0.654086 |
| *TARS2* | | 2 | 7 | 0.654086 |
| *TBC1D16* | | 2 | 7 | 0.654086 |
| *TCTN2* | | 2 | 7 | 0.654086 |
| *TDRD1* | | 2 | 7 | 0.654086 |
| *UMODL1* | | 2 | 7 | 0.654086 |
| *ZC3H6* | | 2 | 7 | 0.654086 |
| *ZNF382* | | 2 | 7 | 0.654086 |
| *ZNF516* | | 2 | 7 | 0.654086 |
| *ZNF568* | | 2 | 7 | 0.654086 |
| *ZNF710* | | 2 | 7 | 0.654086 |
| *C15orf52* | | 8 | 2 | 0.654086 |
| *CDH19* | | 8 | 2 | 0.654086 |
| *CNOT3* | | 8 | 2 | 0.654086 |
| *COL27A1* | | 8 | 2 | 0.654086 |
| *DENND2C* | | 8 | 2 | 0.654086 |
| *DNM1* | | 8 | 2 | 0.654086 |
| *FARP1* | | 8 | 2 | 0.654086 |
| *GPR64* | | 8 | 2 | 0.654086 |
| *KIAA0922* | | 8 | 2 | 0.654086 |
| *MUC2* | | 8 | 2 | 0.654086 |
| *MYOT* | | 8 | 2 | 0.654086 |
| *NF2* | | 8 | 2 | 0.654086 |
| *NRXN3* | | 8 | 2 | 0.654086 |
| *PIK3CG* | | 8 | 2 | 0.654086 |
| *PLEKHG4* | | 8 | 2 | 0.654086 |
| *RNF10* | | 8 | 2 | 0.654086 |
| *SON* | | 8 | 2 | 0.654086 |
| *TMEM132A* | | 8 | 2 | 0.654086 |
| *ZFP37* | | 8 | 2 | 0.654086 |
| *RELN* | | 12 | 21 | 0.654086 |
| *ARHGAP31* | | 6 | 13 | 0.654086 |
| *CCDC108* | | 6 | 13 | 0.654086 |
| *DOCK9* | | 6 | 13 | 0.654086 |
| *RBBP6* | | 6 | 13 | 0.654086 |
| *SOX5* | | 6 | 13 | 0.654086 |
| *LMTK2* | | 14 | 6 | 0.654086 |
| *TENM1* | | 9 | 17 | 0.654086 |
| *RNF216* | | 11 | 4 | 0.654086 |
| *SAMD9* | | 11 | 4 | 0.654086 |
| *USP9X* | | 11 | 4 | 0.654086 |
| *ZFC3H1* | | 11 | 4 | 0.654086 |
| *AMER3* | | 4 | 10 | 0.654086 |
| *ANO4* | | 4 | 10 | 0.654086 |
| *ARHGEF12* | | 4 | 10 | 0.654086 |
| *CDH8* | | 4 | 10 | 0.654086 |
| *CLASP1* | | 4 | 10 | 0.654086 |
| *CNTN5* | | 4 | 10 | 0.654086 |
| *DCDC1* | | 4 | 10 | 0.654086 |
| *DNHD1* | | 4 | 10 | 0.654086 |
| *FOXQ1* | | 4 | 10 | 0.654086 |
| *FSIP2* | | 4 | 10 | 0.654086 |
| *LRRC16A* | | 4 | 10 | 0.654086 |
| *NUP153* | | 4 | 10 | 0.654086 |
| *PALB2* | | 4 | 10 | 0.654086 |
| *PIBF1* | | 4 | 10 | 0.654086 |
| *PRRC2C* | | 4 | 10 | 0.654086 |
| *PUM2* | | 4 | 10 | 0.654086 |
| *VPS8* | | 4 | 10 | 0.654086 |
| *ATM* | | 22 | 33 | 0.654086 |
| *SPTAN1* | | 18 | 28 | 0.654086 |
| *ACSBG2* | | 6 | 1 | 0.654086 |
| *ACTA2* | | 6 | 1 | 0.654086 |
| *ARHGEF15* | | 6 | 1 | 0.654086 |
| *ATP11B* | | 6 | 1 | 0.654086 |
| *CCT8L2* | | 6 | 1 | 0.654086 |
| *CEACAM5* | | 6 | 1 | 0.654086 |
| *CEP63* | | 6 | 1 | 0.654086 |
| *CHMP2A* | | 6 | 1 | 0.654086 |
| *CTD-3074O7.11* | | 6 | 1 | 0.654086 |
| *EFCAB13* | | 6 | 1 | 0.654086 |
| *FAM134A* | | 6 | 1 | 0.654086 |
| *FHDC1* | | 6 | 1 | 0.654086 |
| *FRMPD3* | | 6 | 1 | 0.654086 |
| *GNAI3* | | 6 | 1 | 0.654086 |
| *GNE* | | 6 | 1 | 0.654086 |
| *GPR97* | | 6 | 1 | 0.654086 |
| *HTRA2* | | 6 | 1 | 0.654086 |
| *IRF2BP1* | | 6 | 1 | 0.654086 |
| *KRT79* | | 6 | 1 | 0.654086 |
| *METTL13* | | 6 | 1 | 0.654086 |
| *MINPP1* | | 6 | 1 | 0.654086 |
| *MOV10* | | 6 | 1 | 0.654086 |
| *MYO18A* | | 6 | 1 | 0.654086 |
| *OR2L3* | | 6 | 1 | 0.654086 |
| *PLEKHH3* | | 6 | 1 | 0.654086 |
| *PLS3* | | 6 | 1 | 0.654086 |
| *SHKBP1* | | 6 | 1 | 0.654086 |
| *SLC24A2* | | 6 | 1 | 0.654086 |
| *SMAD3* | | 6 | 1 | 0.654086 |
| *SNW1* | | 6 | 1 | 0.654086 |
| *TBRG4* | | 6 | 1 | 0.654086 |
| *TFAP2C* | | 6 | 1 | 0.654086 |
| *TNFAIP3* | | 6 | 1 | 0.654086 |
| *TPCN1* | | 6 | 1 | 0.654086 |
| *WDR35* | | 6 | 1 | 0.654086 |
| *ZNF445* | | 6 | 1 | 0.654086 |
| *ACOX1* | | 1 | 5 | 0.654086 |
| *ADAM32* | | 1 | 5 | 0.654086 |
| *ADCY1* | | 1 | 5 | 0.654086 |
| *ADNP* | | 1 | 5 | 0.654086 |
| *AEBP1* | | 1 | 5 | 0.654086 |
| *ALDH1A1* | | 1 | 5 | 0.654086 |
| *ALG10* | | 1 | 5 | 0.654086 |
| *ANAPC7* | | 1 | 5 | 0.654086 |
| *ANGPT4* | | 1 | 5 | 0.654086 |
| *AQPEP* | | 1 | 5 | 0.654086 |
| *ARHGAP18* | | 1 | 5 | 0.654086 |
| *ATP13A1* | | 1 | 5 | 0.654086 |
| *ATXN1* | | 1 | 5 | 0.654086 |
| *AZI2* | | 1 | 5 | 0.654086 |
| *C12orf50* | | 1 | 5 | 0.654086 |
| *C6orf10* | | 1 | 5 | 0.654086 |
| *C9orf41* | | 1 | 5 | 0.654086 |
| *CAPN9* | | 1 | 5 | 0.654086 |
| *CCDC13* | | 1 | 5 | 0.654086 |
| *CCDC173* | | 1 | 5 | 0.654086 |
| *CCDC176* | | 1 | 5 | 0.654086 |
| *CCDC28A* | | 1 | 5 | 0.654086 |
| *CGNL1* | | 1 | 5 | 0.654086 |
| *CHST15* | | 1 | 5 | 0.654086 |
| *CNOT10* | | 1 | 5 | 0.654086 |
| *CRMP1* | | 1 | 5 | 0.654086 |
| *CTNNA1* | | 1 | 5 | 0.654086 |
| *CYP2C18* | | 1 | 5 | 0.654086 |
| *DBT* | | 1 | 5 | 0.654086 |
| *DCST1* | | 1 | 5 | 0.654086 |
| *DCTN4* | | 1 | 5 | 0.654086 |
| *DIS3* | | 1 | 5 | 0.654086 |
| *DNAH12* | | 1 | 5 | 0.654086 |
| *DPAGT1* | | 1 | 5 | 0.654086 |
| *DPH2* | | 1 | 5 | 0.654086 |
| *DUOX2* | | 1 | 5 | 0.654086 |
| *ELK4* | | 1 | 5 | 0.654086 |
| *EPS15L1* | | 1 | 5 | 0.654086 |
| *ERP29* | | 1 | 5 | 0.654086 |
| *EXTL3* | | 1 | 5 | 0.654086 |
| *FAM171B* | | 1 | 5 | 0.654086 |
| *FAM188B* | | 1 | 5 | 0.654086 |
| *FAM65C* | | 1 | 5 | 0.654086 |
| *FARSB* | | 1 | 5 | 0.654086 |
| *FASTKD5* | | 1 | 5 | 0.654086 |
| *FDPS* | | 1 | 5 | 0.654086 |
| *FGD2* | | 1 | 5 | 0.654086 |
| *FPR2* | | 1 | 5 | 0.654086 |
| *FZD7* | | 1 | 5 | 0.654086 |
| *GAN* | | 1 | 5 | 0.654086 |
| *GATAD2A* | | 1 | 5 | 0.654086 |
| *GGCX* | | 1 | 5 | 0.654086 |
| *GIMAP6* | | 1 | 5 | 0.654086 |
| *GLRA1* | | 1 | 5 | 0.654086 |
| *GLTP* | | 1 | 5 | 0.654086 |
| *GPD1* | | 1 | 5 | 0.654086 |
| *HHLA2* | | 1 | 5 | 0.654086 |
| *HLA-A* | | 1 | 5 | 0.654086 |
| *HNRNPUL2* | | 1 | 5 | 0.654086 |
| *HSD17B2* | | 1 | 5 | 0.654086 |
| *HSPA12B* | | 1 | 5 | 0.654086 |
| *HTR1F* | | 1 | 5 | 0.654086 |
| *HTRA3* | | 1 | 5 | 0.654086 |
| *IGF2BP1* | | 1 | 5 | 0.654086 |
| *IL7R* | | 1 | 5 | 0.654086 |
| *INCENP* | | 1 | 5 | 0.654086 |
| *KCNA2* | | 1 | 5 | 0.654086 |
| *KCNA6* | | 1 | 5 | 0.654086 |
| *KDM2A* | | 1 | 5 | 0.654086 |
| *KLK15* | | 1 | 5 | 0.654086 |
| *KNSTRN* | | 1 | 5 | 0.654086 |
| *KRT75* | | 1 | 5 | 0.654086 |
| *KTN1* | | 1 | 5 | 0.654086 |
| *LGR4* | | 1 | 5 | 0.654086 |
| *LGSN* | | 1 | 5 | 0.654086 |
| *LIMD1* | | 1 | 5 | 0.654086 |
| *LOX* | | 1 | 5 | 0.654086 |
| *LRG1* | | 1 | 5 | 0.654086 |
| *LRRC43* | | 1 | 5 | 0.654086 |
| *LRRC8B* | | 1 | 5 | 0.654086 |
| *LST3* | | 1 | 5 | 0.654086 |
| *MCF2* | | 1 | 5 | 0.654086 |
| *MCOLN3* | | 1 | 5 | 0.654086 |
| *MED17* | | 1 | 5 | 0.654086 |
| *MED26* | | 1 | 5 | 0.654086 |
| *METTL24* | | 1 | 5 | 0.654086 |
| *MFSD6* | | 1 | 5 | 0.654086 |
| *MKL2* | | 1 | 5 | 0.654086 |
| *MMP10* | | 1 | 5 | 0.654086 |
| *MOGS* | | 1 | 5 | 0.654086 |
| *MSL3* | | 1 | 5 | 0.654086 |
| *MYCN* | | 1 | 5 | 0.654086 |
| *MYO1G* | | 1 | 5 | 0.654086 |
| *NPR3* | | 1 | 5 | 0.654086 |
| *NUDT16* | | 1 | 5 | 0.654086 |
| *NXPE3* | | 1 | 5 | 0.654086 |
| *OIP5* | | 1 | 5 | 0.654086 |
| *PAF1* | | 1 | 5 | 0.654086 |
| *PAPLN* | | 1 | 5 | 0.654086 |
| *PDHA2* | | 1 | 5 | 0.654086 |
| *PER2* | | 1 | 5 | 0.654086 |
| *PET112* | | 1 | 5 | 0.654086 |
| *PEX19* | | 1 | 5 | 0.654086 |
| *PHF8* | | 1 | 5 | 0.654086 |
| *PLCG2* | | 1 | 5 | 0.654086 |
| *PLXNA1* | | 1 | 5 | 0.654086 |
| *PMPCB* | | 1 | 5 | 0.654086 |
| *POGZ* | | 1 | 5 | 0.654086 |
| *PPOX* | | 1 | 5 | 0.654086 |
| *PPP1CA* | | 1 | 5 | 0.654086 |
| *PRDM13* | | 1 | 5 | 0.654086 |
| *PTGS2* | | 1 | 5 | 0.654086 |
| *PTK2B* | | 1 | 5 | 0.654086 |
| *RAB11FIP4* | | 1 | 5 | 0.654086 |
| *RACGAP1* | | 1 | 5 | 0.654086 |
| *RALY* | | 1 | 5 | 0.654086 |
| *RASA2* | | 1 | 5 | 0.654086 |
| *RASIP1* | | 1 | 5 | 0.654086 |
| *RFX7* | | 1 | 5 | 0.654086 |
| *RHPN1* | | 1 | 5 | 0.654086 |
| *RNF103* | | 1 | 5 | 0.654086 |
| *ROR1* | | 1 | 5 | 0.654086 |
| *SCML2* | | 1 | 5 | 0.654086 |
| *SCNN1B* | | 1 | 5 | 0.654086 |
| *SELE* | | 1 | 5 | 0.654086 |
| *11-Sep* | | 1 | 5 | 0.654086 |
| *SIGLEC8* | | 1 | 5 | 0.654086 |
| *SKIV2L* | | 1 | 5 | 0.654086 |
| *SLC12A1* | | 1 | 5 | 0.654086 |
| *SLC25A13* | | 1 | 5 | 0.654086 |
| *SLC25A2* | | 1 | 5 | 0.654086 |
| *SLCO6A1* | | 1 | 5 | 0.654086 |
| *SNRNP200* | | 1 | 5 | 0.654086 |
| *SPG7* | | 1 | 5 | 0.654086 |
| *SPNS1* | | 1 | 5 | 0.654086 |
| *SYT5* | | 1 | 5 | 0.654086 |
| *TBC1D8* | | 1 | 5 | 0.654086 |
| *TBCE* | | 1 | 5 | 0.654086 |
| *TMEM143* | | 1 | 5 | 0.654086 |
| *TMEM260* | | 1 | 5 | 0.654086 |
| *TRH* | | 1 | 5 | 0.654086 |
| *TRIM56* | | 1 | 5 | 0.654086 |
| *TSR1* | | 1 | 5 | 0.654086 |
| *UTP6* | | 1 | 5 | 0.654086 |
| *WDR45* | | 1 | 5 | 0.654086 |
| *ZC3H7A* | | 1 | 5 | 0.654086 |
| *ZNF438* | | 1 | 5 | 0.654086 |
| *ZNF510* | | 1 | 5 | 0.654086 |
| *ZNF556* | | 1 | 5 | 0.654086 |
| *ZNF662* | | 1 | 5 | 0.654086 |
| *ZNF724P* | | 1 | 5 | 0.654086 |
| *ZNF774* | | 1 | 5 | 0.654086 |
| *DSP* | | 19 | 10 | 0.654086 |
| *MED12* | | 15 | 7 | 0.65753 |
| *CASC5* | | 7 | 14 | 0.65753 |
| *CLIP1* | | 7 | 14 | 0.65753 |
| *RP1L1* | | 7 | 14 | 0.65753 |
| *DNAH8* | | 15 | 24 | 0.690012 |
| *PKHD1L1* | | 15 | 24 | 0.690012 |
| *GRIA1* | | 12 | 5 | 0.690012 |
| *PRDM2* | | 12 | 5 | 0.690012 |
| *ATP8B1* | | 5 | 11 | 0.690012 |
| *BAZ2A* | | 5 | 11 | 0.690012 |
| *C1orf173* | | 5 | 11 | 0.690012 |
| *CHD8* | | 5 | 11 | 0.690012 |
| *COL4A4* | | 5 | 11 | 0.690012 |
| *INTS1* | | 5 | 11 | 0.690012 |
| *KIF21B* | | 5 | 11 | 0.690012 |
| *MYLK* | | 5 | 11 | 0.690012 |
| *NLRP8* | | 5 | 11 | 0.690012 |
| *UACA* | | 5 | 11 | 0.690012 |
| *FAM193A* | | 8 | 15 | 0.690012 |
| *BAZ1B* | | 9 | 3 | 0.690012 |
| *COL6A5* | | 9 | 3 | 0.690012 |
| *CRNKL1* | | 9 | 3 | 0.690012 |
| *DCAF4L2* | | 9 | 3 | 0.690012 |
| *EIF4ENIF1* | | 9 | 3 | 0.690012 |
| *ERCC6* | | 9 | 3 | 0.690012 |
| *HEPHL1* | | 9 | 3 | 0.690012 |
| *7-Mar* | | 9 | 3 | 0.690012 |
| *NDST4* | | 9 | 3 | 0.690012 |
| *RUNX1* | | 9 | 3 | 0.690012 |
| *TRIM51* | | 9 | 3 | 0.690012 |
| *VWA8* | | 9 | 3 | 0.690012 |
| *ARHGEF7* | | 3 | 8 | 0.690012 |
| *ATP2B1* | | 3 | 8 | 0.690012 |
| *ATP6V0A1* | | 3 | 8 | 0.690012 |
| *ATP8A1* | | 3 | 8 | 0.690012 |
| *C8orf34* | | 3 | 8 | 0.690012 |
| *CCHCR1* | | 3 | 8 | 0.690012 |
| *CHGB* | | 3 | 8 | 0.690012 |
| *CLSPN* | | 3 | 8 | 0.690012 |
| *COL4A2* | | 3 | 8 | 0.690012 |
| *EPHB3* | | 3 | 8 | 0.690012 |
| *GAPVD1* | | 3 | 8 | 0.690012 |
| *GUCY1A3* | | 3 | 8 | 0.690012 |
| *IQGAP1* | | 3 | 8 | 0.690012 |
| *LRFN2* | | 3 | 8 | 0.690012 |
| *NLRP2* | | 3 | 8 | 0.690012 |
| *PCDH7* | | 3 | 8 | 0.690012 |
| *PCDHA1* | | 3 | 8 | 0.690012 |
| *PCDHA8* | | 3 | 8 | 0.690012 |
| *PLEKHG2* | | 3 | 8 | 0.690012 |
| *PLXNB1* | | 3 | 8 | 0.690012 |
| *PLXND1* | | 3 | 8 | 0.690012 |
| *PMFBP1* | | 3 | 8 | 0.690012 |
| *RAB3GAP2* | | 3 | 8 | 0.690012 |
| *RBM17* | | 3 | 8 | 0.690012 |
| *SCG2* | | 3 | 8 | 0.690012 |
| *SFI1* | | 3 | 8 | 0.690012 |
| *SPECC1* | | 3 | 8 | 0.690012 |
| *SYMPK* | | 3 | 8 | 0.690012 |
| *TFG* | | 3 | 8 | 0.690012 |
| *TTLL5* | | 3 | 8 | 0.690012 |
| *USP31* | | 3 | 8 | 0.690012 |
| *VCAM1* | | 3 | 8 | 0.690012 |
| *XDH* | | 3 | 8 | 0.690012 |
| *ZNF800* | | 3 | 8 | 0.690012 |
| *NEB* | | 21 | 31 | 0.690012 |
| *DCHS2* | | 12 | 20 | 0.697198 |
| *LRRK2* | | 12 | 20 | 0.697198 |
| *AKAP9* | | 17 | 26 | 0.697198 |
| *CACNA1A* | | 9 | 16 | 0.697198 |
| *APOB* | | 13 | 21 | 0.697198 |
| *LRP1* | | 13 | 21 | 0.697198 |
| *MED13* | | 13 | 21 | 0.697198 |
| *BUB1* | | 6 | 12 | 0.697198 |
| *FLNC* | | 6 | 12 | 0.697198 |
| *SCN2A* | | 6 | 12 | 0.697198 |
| *SHROOM3* | | 6 | 12 | 0.697198 |
| *SYNJ1* | | 6 | 12 | 0.697198 |
| *TIAM1* | | 6 | 12 | 0.697198 |
| *WDR17* | | 6 | 12 | 0.697198 |
| *ZNFX1* | | 6 | 12 | 0.697198 |
| *ACTB* | | 13 | 6 | 0.697198 |
| *KDM5A* | | 13 | 6 | 0.697198 |
| *CREBBP* | | 29 | 19 | 0.697198 |
| *DIDO1* | | 14 | 22 | 0.697198 |
| *COL6A6* | | 10 | 17 | 0.697198 |
| *HUWE1* | | 10 | 17 | 0.697198 |
| *SPEN* | | 10 | 17 | 0.697198 |
| *ABCC12* | | 4 | 9 | 0.697198 |
| *ALK* | | 4 | 9 | 0.697198 |
| *CCDC36* | | 4 | 9 | 0.697198 |
| *CDK13* | | 4 | 9 | 0.697198 |
| *CEP290* | | 4 | 9 | 0.697198 |
| *DIP2C* | | 4 | 9 | 0.697198 |
| *DOCK3* | | 4 | 9 | 0.697198 |
| *FBXO42* | | 4 | 9 | 0.697198 |
| *FSTL5* | | 4 | 9 | 0.697198 |
| *HIST1H3B* | | 4 | 9 | 0.697198 |
| *IGF2R* | | 4 | 9 | 0.697198 |
| *LRGUK* | | 4 | 9 | 0.697198 |
| *MED13L* | | 4 | 9 | 0.697198 |
| *NLRP3* | | 4 | 9 | 0.697198 |
| *NPAS4* | | 4 | 9 | 0.697198 |
| *OAS2* | | 4 | 9 | 0.697198 |
| *PCDH1* | | 4 | 9 | 0.697198 |
| *PRUNE2* | | 4 | 9 | 0.697198 |
| *PTPRG* | | 4 | 9 | 0.697198 |
| *SLC12A5* | | 4 | 9 | 0.697198 |
| *SULF1* | | 4 | 9 | 0.697198 |
| *TSHZ2* | | 4 | 9 | 0.697198 |
| *USP48* | | 4 | 9 | 0.697198 |
| *ZEB1* | | 4 | 9 | 0.697198 |
| *ZNF569* | | 4 | 9 | 0.697198 |
| *ZNF737* | | 4 | 9 | 0.697198 |
| *CDH16* | | 10 | 4 | 0.697198 |
| *CFH* | | 10 | 4 | 0.697198 |
| *GOLGA3* | | 10 | 4 | 0.697198 |
| *KCNQ5* | | 10 | 4 | 0.697198 |
| *PLCL1* | | 10 | 4 | 0.697198 |
| *SPEG* | | 10 | 4 | 0.697198 |
| *SYBU* | | 10 | 4 | 0.697198 |
| *USP8* | | 10 | 4 | 0.697198 |
| *WAC* | | 10 | 4 | 0.697198 |
| *ABI3BP* | | 7 | 2 | 0.697198 |
| *CCDC144A* | | 7 | 2 | 0.697198 |
| *CNKSR3* | | 7 | 2 | 0.697198 |
| *CRTC3* | | 7 | 2 | 0.697198 |
| *CTAGE1* | | 7 | 2 | 0.697198 |
| *DLGAP5* | | 7 | 2 | 0.697198 |
| *EEF2K* | | 7 | 2 | 0.697198 |
| *EIF5B* | | 7 | 2 | 0.697198 |
| *GOLGA1* | | 7 | 2 | 0.697198 |
| *GOLGA5* | | 7 | 2 | 0.697198 |
| *IFT140* | | 7 | 2 | 0.697198 |
| *KCNH4* | | 7 | 2 | 0.697198 |
| *KIAA1683* | | 7 | 2 | 0.697198 |
| *LETMD1* | | 7 | 2 | 0.697198 |
| *NAT10* | | 7 | 2 | 0.697198 |
| *NEURL4* | | 7 | 2 | 0.697198 |
| *PCDHB3* | | 7 | 2 | 0.697198 |
| *SKIV2L2* | | 7 | 2 | 0.697198 |
| *SUN1* | | 7 | 2 | 0.697198 |
| *TLL2* | | 7 | 2 | 0.697198 |
| *TOX3* | | 7 | 2 | 0.697198 |
| *TSC22D1* | | 7 | 2 | 0.697198 |
| *USP21* | | 7 | 2 | 0.697198 |
| *WEE1* | | 7 | 2 | 0.697198 |
| *WWC3* | | 7 | 2 | 0.697198 |
| *ZC3H14* | | 7 | 2 | 0.697198 |
| *ZC3H4* | | 7 | 2 | 0.697198 |
| *ABL2* | | 2 | 6 | 0.697198 |
| *ACOX3* | | 2 | 6 | 0.697198 |
| *AGO2* | | 2 | 6 | 0.697198 |
| *AMZ2* | | 2 | 6 | 0.697198 |
| *AP4E1* | | 2 | 6 | 0.697198 |
| *ASMTL* | | 2 | 6 | 0.697198 |
| *ATP1A3* | | 2 | 6 | 0.697198 |
| *BEST3* | | 2 | 6 | 0.697198 |
| *C1orf168* | | 2 | 6 | 0.697198 |
| *CCNE2* | | 2 | 6 | 0.697198 |
| *CDH15* | | 2 | 6 | 0.697198 |
| *CDH5* | | 2 | 6 | 0.697198 |
| *CDKAL1* | | 2 | 6 | 0.697198 |
| *CFHR5* | | 2 | 6 | 0.697198 |
| *CIITA* | | 2 | 6 | 0.697198 |
| *CNNM2* | | 2 | 6 | 0.697198 |
| *COL4A3BP* | | 2 | 6 | 0.697198 |
| *CPSF3* | | 2 | 6 | 0.697198 |
| *DHX16* | | 2 | 6 | 0.697198 |
| *DNAAF1* | | 2 | 6 | 0.697198 |
| *DNMT1* | | 2 | 6 | 0.697198 |
| *DPY19L2* | | 2 | 6 | 0.697198 |
| *EML2* | | 2 | 6 | 0.697198 |
| *EXOC3* | | 2 | 6 | 0.697198 |
| *FAM161A* | | 2 | 6 | 0.697198 |
| *FAM217A* | | 2 | 6 | 0.697198 |
| *FZD10* | | 2 | 6 | 0.697198 |
| *GALNTL5* | | 2 | 6 | 0.697198 |
| *GNAS* | | 2 | 6 | 0.697198 |
| *GRM3* | | 2 | 6 | 0.697198 |
| *GRM8* | | 2 | 6 | 0.697198 |
| *IGSF3* | | 2 | 6 | 0.697198 |
| *INF2* | | 2 | 6 | 0.697198 |
| *INTS5* | | 2 | 6 | 0.697198 |
| *ITGA1* | | 2 | 6 | 0.697198 |
| *ITGB2* | | 2 | 6 | 0.697198 |
| *KCNB1* | | 2 | 6 | 0.697198 |
| *KIAA1549L* | | 2 | 6 | 0.697198 |
| *KIF2B* | | 2 | 6 | 0.697198 |
| *LMBRD2* | | 2 | 6 | 0.697198 |
| *LRMP* | | 2 | 6 | 0.697198 |
| *LRRC6* | | 2 | 6 | 0.697198 |
| *MEGF10* | | 2 | 6 | 0.697198 |
| *NCKAP5L* | | 2 | 6 | 0.697198 |
| *NDE1* | | 2 | 6 | 0.697198 |
| *NOP2* | | 2 | 6 | 0.697198 |
| *NPHP1* | | 2 | 6 | 0.697198 |
| *NRP2* | | 2 | 6 | 0.697198 |
| *OR4C15* | | 2 | 6 | 0.697198 |
| *PCDHB13* | | 2 | 6 | 0.697198 |
| *PCDHGB1* | | 2 | 6 | 0.697198 |
| *PIK3CB* | | 2 | 6 | 0.697198 |
| *PIWIL2* | | 2 | 6 | 0.697198 |
| *PKP4* | | 2 | 6 | 0.697198 |
| *POLG* | | 2 | 6 | 0.697198 |
| *POLM* | | 2 | 6 | 0.697198 |
| *PRPF4B* | | 2 | 6 | 0.697198 |
| *PRPF8* | | 2 | 6 | 0.697198 |
| *PTCHD1* | | 2 | 6 | 0.697198 |
| *PTPDC1* | | 2 | 6 | 0.697198 |
| *PTPRH* | | 2 | 6 | 0.697198 |
| *RASAL1* | | 2 | 6 | 0.697198 |
| *RBM44* | | 2 | 6 | 0.697198 |
| *RFX6* | | 2 | 6 | 0.697198 |
| *RGS7* | | 2 | 6 | 0.697198 |
| *SAMD4A* | | 2 | 6 | 0.697198 |
| *SCAF1* | | 2 | 6 | 0.697198 |
| *SEC24A* | | 2 | 6 | 0.697198 |
| *14-Sep* | | 2 | 6 | 0.697198 |
| *SFSWAP* | | 2 | 6 | 0.697198 |
| *SHC1* | | 2 | 6 | 0.697198 |
| *SHROOM4* | | 2 | 6 | 0.697198 |
| *SLC24A3* | | 2 | 6 | 0.697198 |
| *SLC26A4* | | 2 | 6 | 0.697198 |
| *SLC6A3* | | 2 | 6 | 0.697198 |
| *SNED1* | | 2 | 6 | 0.697198 |
| *SOGA1* | | 2 | 6 | 0.697198 |
| *SRRM4* | | 2 | 6 | 0.697198 |
| *SYNJ2* | | 2 | 6 | 0.697198 |
| *TARS* | | 2 | 6 | 0.697198 |
| *TAX1BP1* | | 2 | 6 | 0.697198 |
| *TBC1D5* | | 2 | 6 | 0.697198 |
| *TBCK* | | 2 | 6 | 0.697198 |
| *TBK1* | | 2 | 6 | 0.697198 |
| *TCHP* | | 2 | 6 | 0.697198 |
| *TEK* | | 2 | 6 | 0.697198 |
| *TLR8* | | 2 | 6 | 0.697198 |
| *TMX4* | | 2 | 6 | 0.697198 |
| *TPTE2* | | 2 | 6 | 0.697198 |
| *TSC22D2* | | 2 | 6 | 0.697198 |
| *TTC30A* | | 2 | 6 | 0.697198 |
| *U2AF1* | | 2 | 6 | 0.697198 |
| *UHRF2* | | 2 | 6 | 0.697198 |
| *UNC5D* | | 2 | 6 | 0.697198 |
| *WDR19* | | 2 | 6 | 0.697198 |
| *ZFP30* | | 2 | 6 | 0.697198 |
| *ZKSCAN2* | | 2 | 6 | 0.697198 |
| *ZNF280A* | | 2 | 6 | 0.697198 |
| *ZNF425* | | 2 | 6 | 0.697198 |
| *ZNF430* | | 2 | 6 | 0.697198 |
| *ZNF432* | | 2 | 6 | 0.697198 |
| *ZNF439* | | 2 | 6 | 0.697198 |
| *ZNF532* | | 2 | 6 | 0.697198 |
| *ZNF675* | | 2 | 6 | 0.697198 |
| *ZNF730* | | 2 | 6 | 0.697198 |
| *BRCA2* | | 15 | 23 | 0.703198 |
| *PTPN13* | | 14 | 7 | 0.703198 |
| *ALPK2* | | 7 | 13 | 0.703198 |
| *BOD1L1* | | 7 | 13 | 0.703198 |
| *BRIP1* | | 7 | 13 | 0.703198 |
| *NBPF1* | | 7 | 13 | 0.703198 |
| *NUP188* | | 7 | 13 | 0.703198 |
| *RYR3* | | 17 | 25 | 0.727049 |
| *CHD4* | | 8 | 14 | 0.727049 |
| *GOLGA4* | | 8 | 14 | 0.727049 |
| *NKTR* | | 8 | 14 | 0.727049 |
| *DNAJC13* | | 15 | 8 | 0.727049 |
| *NCKAP5* | | 15 | 8 | 0.727049 |
| *UTP20* | | 15 | 8 | 0.727049 |
| *KMT2C* | | 43 | 32 | 0.727049 |
| *AFF2* | | 5 | 10 | 0.727049 |
| *ATG2B* | | 5 | 10 | 0.727049 |
| *DGKG* | | 5 | 10 | 0.727049 |
| *DMBT1* | | 5 | 10 | 0.727049 |
| *EHBP1* | | 5 | 10 | 0.727049 |
| *GRIK2* | | 5 | 10 | 0.727049 |
| *KDR* | | 5 | 10 | 0.727049 |
| *MYO7B* | | 5 | 10 | 0.727049 |
| *MYPN* | | 5 | 10 | 0.727049 |
| *PAPPA* | | 5 | 10 | 0.727049 |
| *PIK3C2B* | | 5 | 10 | 0.727049 |
| *PPFIA2* | | 5 | 10 | 0.727049 |
| *RAD21* | | 5 | 10 | 0.727049 |
| *SAMD9L* | | 5 | 10 | 0.727049 |
| *SCN10A* | | 5 | 10 | 0.727049 |
| *ITGB4* | | 11 | 5 | 0.727049 |
| *KRAS* | | 11 | 5 | 0.727049 |
| *PPRC1* | | 11 | 5 | 0.727049 |
| *TPO* | | 11 | 5 | 0.727049 |
| *ZNF831* | | 11 | 5 | 0.727049 |
| *CSMD3* | | 24 | 33 | 0.727049 |
| *KMT2A* | | 27 | 18 | 0.727049 |
| *ARID1B* | | 9 | 15 | 0.727049 |
| *C2orf16* | | 9 | 15 | 0.727049 |
| *TP53BP1* | | 9 | 15 | 0.727049 |
| *TTC3* | | 9 | 15 | 0.727049 |
| *ACHE* | | 5 | 1 | 0.727049 |
| *ACO2* | | 5 | 1 | 0.727049 |
| *ADAM19* | | 5 | 1 | 0.727049 |
| *APPL1* | | 5 | 1 | 0.727049 |
| *BORA* | | 5 | 1 | 0.727049 |
| *BRI3BP* | | 5 | 1 | 0.727049 |
| *C9orf131* | | 5 | 1 | 0.727049 |
| *CACNA1I* | | 5 | 1 | 0.727049 |
| *CCDC138* | | 5 | 1 | 0.727049 |
| *CCDC27* | | 5 | 1 | 0.727049 |
| *CCDC63* | | 5 | 1 | 0.727049 |
| *CEP78* | | 5 | 1 | 0.727049 |
| *CERCAM* | | 5 | 1 | 0.727049 |
| *CLCN3* | | 5 | 1 | 0.727049 |
| *CMIP* | | 5 | 1 | 0.727049 |
| *COL4A6* | | 5 | 1 | 0.727049 |
| *CSDE1* | | 5 | 1 | 0.727049 |
| *CSK* | | 5 | 1 | 0.727049 |
| *CSNK1A1* | | 5 | 1 | 0.727049 |
| *CYP2F1* | | 5 | 1 | 0.727049 |
| *EXD3* | | 5 | 1 | 0.727049 |
| *FANCC* | | 5 | 1 | 0.727049 |
| *FBXO22* | | 5 | 1 | 0.727049 |
| *FBXO38* | | 5 | 1 | 0.727049 |
| *FOXN2* | | 5 | 1 | 0.727049 |
| *GALNT8* | | 5 | 1 | 0.727049 |
| *GC* | | 5 | 1 | 0.727049 |
| *GIMAP1* | | 5 | 1 | 0.727049 |
| *GMPS* | | 5 | 1 | 0.727049 |
| *HAL* | | 5 | 1 | 0.727049 |
| *HHIP* | | 5 | 1 | 0.727049 |
| *HMGCLL1* | | 5 | 1 | 0.727049 |
| *HOXA7* | | 5 | 1 | 0.727049 |
| *HOXB1* | | 5 | 1 | 0.727049 |
| *IFI44L* | | 5 | 1 | 0.727049 |
| *INPP5A* | | 5 | 1 | 0.727049 |
| *IRF2* | | 5 | 1 | 0.727049 |
| *KIAA1324L* | | 5 | 1 | 0.727049 |
| *KLHDC8B* | | 5 | 1 | 0.727049 |
| *LAMC2* | | 5 | 1 | 0.727049 |
| *LATS2* | | 5 | 1 | 0.727049 |
| *LCA5* | | 5 | 1 | 0.727049 |
| *LIMK1* | | 5 | 1 | 0.727049 |
| *LINGO3* | | 5 | 1 | 0.727049 |
| *LLGL2* | | 5 | 1 | 0.727049 |
| *MAMDC4* | | 5 | 1 | 0.727049 |
| *6-Mar* | | 5 | 1 | 0.727049 |
| *MEF2C* | | 5 | 1 | 0.727049 |
| *MINK1* | | 5 | 1 | 0.727049 |
| *MMEL1* | | 5 | 1 | 0.727049 |
| *MRPL44* | | 5 | 1 | 0.727049 |
| *MTMR2* | | 5 | 1 | 0.727049 |
| *MYBPC3* | | 5 | 1 | 0.727049 |
| *NLRX1* | | 5 | 1 | 0.727049 |
| *NTRK3* | | 5 | 1 | 0.727049 |
| *NUP43* | | 5 | 1 | 0.727049 |
| *ORC1* | | 5 | 1 | 0.727049 |
| *OXSM* | | 5 | 1 | 0.727049 |
| *PDE6C* | | 5 | 1 | 0.727049 |
| *PIAS3* | | 5 | 1 | 0.727049 |
| *PIK3AP1* | | 5 | 1 | 0.727049 |
| *PIK3R3* | | 5 | 1 | 0.727049 |
| *PIWIL3* | | 5 | 1 | 0.727049 |
| *PLAGL2* | | 5 | 1 | 0.727049 |
| *PLEKHA8* | | 5 | 1 | 0.727049 |
| *PLEKHG1* | | 5 | 1 | 0.727049 |
| *PRDM14* | | 5 | 1 | 0.727049 |
| *PRDX4* | | 5 | 1 | 0.727049 |
| *PRSS36* | | 5 | 1 | 0.727049 |
| *PTBP3* | | 5 | 1 | 0.727049 |
| *RAD51C* | | 5 | 1 | 0.727049 |
| *RHBDL3* | | 5 | 1 | 0.727049 |
| *RUFY2* | | 5 | 1 | 0.727049 |
| *S100PBP* | | 5 | 1 | 0.727049 |
| *SCNN1D* | | 5 | 1 | 0.727049 |
| *SERPINE1* | | 5 | 1 | 0.727049 |
| *SKIDA1* | | 5 | 1 | 0.727049 |
| *SLC26A7* | | 5 | 1 | 0.727049 |
| *SLC8A3* | | 5 | 1 | 0.727049 |
| *SOCS6* | | 5 | 1 | 0.727049 |
| *SRGAP1* | | 5 | 1 | 0.727049 |
| *ST7* | | 5 | 1 | 0.727049 |
| *STK39* | | 5 | 1 | 0.727049 |
| *TGM1* | | 5 | 1 | 0.727049 |
| *TNKS* | | 5 | 1 | 0.727049 |
| *TP73* | | 5 | 1 | 0.727049 |
| *TTLL12* | | 5 | 1 | 0.727049 |
| *UBA3* | | 5 | 1 | 0.727049 |
| *UBXN6* | | 5 | 1 | 0.727049 |
| *USP1* | | 5 | 1 | 0.727049 |
| *WDR76* | | 5 | 1 | 0.727049 |
| *YAP1* | | 5 | 1 | 0.727049 |
| *AASDH* | | 8 | 3 | 0.727049 |
| *BCL9L* | | 8 | 3 | 0.727049 |
| *CD1D* | | 8 | 3 | 0.727049 |
| *DISP1* | | 8 | 3 | 0.727049 |
| *EPHB2* | | 8 | 3 | 0.727049 |
| *FAM71A* | | 8 | 3 | 0.727049 |
| *FRMPD1* | | 8 | 3 | 0.727049 |
| *MCM4* | | 8 | 3 | 0.727049 |
| *MPHOSPH8* | | 8 | 3 | 0.727049 |
| *PCDHA6* | | 8 | 3 | 0.727049 |
| *PIDD* | | 8 | 3 | 0.727049 |
| *SLC45A1* | | 8 | 3 | 0.727049 |
| *ADAMTS15* | | 3 | 7 | 0.727049 |
| *AKAP12* | | 3 | 7 | 0.727049 |
| *AMIGO3* | | 3 | 7 | 0.727049 |
| *ARNT* | | 3 | 7 | 0.727049 |
| *ATP13A4* | | 3 | 7 | 0.727049 |
| *BPIFB6* | | 3 | 7 | 0.727049 |
| *BRD7* | | 3 | 7 | 0.727049 |
| *BRINP1* | | 3 | 7 | 0.727049 |
| *BRPF1* | | 3 | 7 | 0.727049 |
| *CAPRIN2* | | 3 | 7 | 0.727049 |
| *CHSY1* | | 3 | 7 | 0.727049 |
| *COL11A2* | | 3 | 7 | 0.727049 |
| *COL16A1* | | 3 | 7 | 0.727049 |
| *DBF4* | | 3 | 7 | 0.727049 |
| *EPHA8* | | 3 | 7 | 0.727049 |
| *EXT1* | | 3 | 7 | 0.727049 |
| *FER* | | 3 | 7 | 0.727049 |
| *FIGN* | | 3 | 7 | 0.727049 |
| *FNIP1* | | 3 | 7 | 0.727049 |
| *GABRA1* | | 3 | 7 | 0.727049 |
| *INTS2* | | 3 | 7 | 0.727049 |
| *JAK1* | | 3 | 7 | 0.727049 |
| *KLHL40* | | 3 | 7 | 0.727049 |
| *KLHL6* | | 3 | 7 | 0.727049 |
| *LGR5* | | 3 | 7 | 0.727049 |
| *MTUS1* | | 3 | 7 | 0.727049 |
| *MVP* | | 3 | 7 | 0.727049 |
| *MYO3B* | | 3 | 7 | 0.727049 |
| *NLRC4* | | 3 | 7 | 0.727049 |
| *NXF1* | | 3 | 7 | 0.727049 |
| *PAPOLA* | | 3 | 7 | 0.727049 |
| *PCDHB1* | | 3 | 7 | 0.727049 |
| *PCDHGA1* | | 3 | 7 | 0.727049 |
| *PHKB* | | 3 | 7 | 0.727049 |
| *PIGO* | | 3 | 7 | 0.727049 |
| *PIK3C2G* | | 3 | 7 | 0.727049 |
| *POLR2B* | | 3 | 7 | 0.727049 |
| *POLR3A* | | 3 | 7 | 0.727049 |
| *POM121* | | 3 | 7 | 0.727049 |
| *PRKD1* | | 3 | 7 | 0.727049 |
| *PTPN9* | | 3 | 7 | 0.727049 |
| *RBBP8* | | 3 | 7 | 0.727049 |
| *RRBP1* | | 3 | 7 | 0.727049 |
| *SIPA1L1* | | 3 | 7 | 0.727049 |
| *SLCO5A1* | | 3 | 7 | 0.727049 |
| *SLX4IP* | | 3 | 7 | 0.727049 |
| *TNIK* | | 3 | 7 | 0.727049 |
| *TNRC6C* | | 3 | 7 | 0.727049 |
| *TRIM42* | | 3 | 7 | 0.727049 |
| *TRPV5* | | 3 | 7 | 0.727049 |
| *TTF2* | | 3 | 7 | 0.727049 |
| *USP9Y* | | 3 | 7 | 0.727049 |
| *WDHD1* | | 3 | 7 | 0.727049 |
| *WDR78* | | 3 | 7 | 0.727049 |
| *ZMIZ2* | | 3 | 7 | 0.727049 |
| *ZNF14* | | 3 | 7 | 0.727049 |
| *ZNF28* | | 3 | 7 | 0.727049 |
| *ZNF546* | | 3 | 7 | 0.727049 |
| *ZNF699* | | 3 | 7 | 0.727049 |
| *ZNF700* | | 3 | 7 | 0.727049 |
| *ZNF791* | | 3 | 7 | 0.727049 |
| *SYNE2* | | 29 | 20 | 0.734292 |
| *GPATCH8* | | 6 | 11 | 0.744789 |
| *GRM5* | | 6 | 11 | 0.744789 |
| *NBEA* | | 6 | 11 | 0.744789 |
| *RGS22* | | 6 | 11 | 0.744789 |
| *RIPK4* | | 6 | 11 | 0.744789 |
| *TRERF1* | | 6 | 11 | 0.744789 |
| *SUPT6H* | | 12 | 6 | 0.744789 |
| *TDRD6* | | 12 | 6 | 0.744789 |
| *CUBN* | | 21 | 29 | 0.752086 |
| *C5orf42* | | 10 | 16 | 0.752086 |
| *DNAH2* | | 10 | 16 | 0.752086 |
| *IGSF10* | | 10 | 16 | 0.752086 |
| *SPHKAP* | | 10 | 16 | 0.752086 |
| *UBR4* | | 24 | 16 | 0.752086 |
| *NAV3* | | 11 | 17 | 0.752086 |
| *SRRM2* | | 11 | 17 | 0.752086 |
| *EPHA2* | | 13 | 7 | 0.752086 |
| *RBM10* | | 13 | 7 | 0.752086 |
| *ACAN* | | 7 | 12 | 0.752086 |
| *CELSR1* | | 7 | 12 | 0.752086 |
| *COL5A2* | | 7 | 12 | 0.752086 |
| *DYNC2H1* | | 7 | 12 | 0.752086 |
| *HIVEP2* | | 7 | 12 | 0.752086 |
| *MECOM* | | 7 | 12 | 0.752086 |
| *MYH13* | | 7 | 12 | 0.752086 |
| *RIF1* | | 7 | 12 | 0.752086 |
| *TIAM2* | | 7 | 12 | 0.752086 |
| *ABCA4* | | 4 | 8 | 0.752086 |
| *ACTG1* | | 4 | 8 | 0.752086 |
| *ARVCF* | | 4 | 8 | 0.752086 |
| *BCOR* | | 4 | 8 | 0.752086 |
| *BUD13* | | 4 | 8 | 0.752086 |
| *CADPS2* | | 4 | 8 | 0.752086 |
| *CCDC132* | | 4 | 8 | 0.752086 |
| *CDHR2* | | 4 | 8 | 0.752086 |
| *CEP152* | | 4 | 8 | 0.752086 |
| *CEP97* | | 4 | 8 | 0.752086 |
| *CFHR4* | | 4 | 8 | 0.752086 |
| *CKAP5* | | 4 | 8 | 0.752086 |
| *CSRNP3* | | 4 | 8 | 0.752086 |
| *CYP11B2* | | 4 | 8 | 0.752086 |
| *DGKH* | | 4 | 8 | 0.752086 |
| *EPHA6* | | 4 | 8 | 0.752086 |
| *EXOC4* | | 4 | 8 | 0.752086 |
| *FASTKD3* | | 4 | 8 | 0.752086 |
| *HLCS* | | 4 | 8 | 0.752086 |
| *IGSF9B* | | 4 | 8 | 0.752086 |
| *KAT7* | | 4 | 8 | 0.752086 |
| *KHDRBS2* | | 4 | 8 | 0.752086 |
| *MIPOL1* | | 4 | 8 | 0.752086 |
| *MON2* | | 4 | 8 | 0.752086 |
| *NISCH* | | 4 | 8 | 0.752086 |
| *PARP8* | | 4 | 8 | 0.752086 |
| *PLB1* | | 4 | 8 | 0.752086 |
| *PLEKHA7* | | 4 | 8 | 0.752086 |
| *PRPF6* | | 4 | 8 | 0.752086 |
| *RGPD4* | | 4 | 8 | 0.752086 |
| *SCAPER* | | 4 | 8 | 0.752086 |
| *SEMA6D* | | 4 | 8 | 0.752086 |
| *THADA* | | 4 | 8 | 0.752086 |
| *TMCO4* | | 4 | 8 | 0.752086 |
| *ZNF394* | | 4 | 8 | 0.752086 |
| *ZNF407* | | 4 | 8 | 0.752086 |
| *ARHGAP29* | | 9 | 4 | 0.752086 |
| *ATP10D* | | 9 | 4 | 0.752086 |
| *C20orf26* | | 9 | 4 | 0.752086 |
| *DPP10* | | 9 | 4 | 0.752086 |
| *LTN1* | | 9 | 4 | 0.752086 |
| *NLRP4* | | 9 | 4 | 0.752086 |
| *NPC1L1* | | 9 | 4 | 0.752086 |
| *PCDHA7* | | 9 | 4 | 0.752086 |
| *PRDM5* | | 9 | 4 | 0.752086 |
| *PTEN* | | 9 | 4 | 0.752086 |
| *RGS12* | | 9 | 4 | 0.752086 |
| *TCF20* | | 9 | 4 | 0.752086 |
| *TRAF3IP2* | | 9 | 4 | 0.752086 |
| *ZNF160* | | 9 | 4 | 0.752086 |
| *HSPG2* | | 12 | 18 | 0.752086 |
| *PCLO* | | 18 | 25 | 0.752086 |
| *KIAA0947* | | 8 | 13 | 0.752086 |
| *MYH4* | | 8 | 13 | 0.752086 |
| *PSIP1* | | 8 | 13 | 0.752086 |
| *ZNF292* | | 8 | 13 | 0.752086 |
| *EFCAB5* | | 14 | 8 | 0.752086 |
| *F5* | | 14 | 8 | 0.752086 |
| *MICAL2* | | 14 | 8 | 0.752086 |
| *LYST* | | 13 | 19 | 0.752086 |
| *ABCB8* | | 2 | 5 | 0.752086 |
| *ACAD11* | | 6 | 2 | 0.752086 |
| *ACP5* | | 6 | 2 | 0.752086 |
| *ACSM2B* | | 2 | 5 | 0.752086 |
| *ACSS1* | | 2 | 5 | 0.752086 |
| *ADAMTS4* | | 6 | 2 | 0.752086 |
| *ADAMTS8* | | 2 | 5 | 0.752086 |
| *ADRM1* | | 2 | 5 | 0.752086 |
| *AFAP1L2* | | 6 | 2 | 0.752086 |
| *AKAP3* | | 2 | 5 | 0.752086 |
| *ALDH1L2* | | 2 | 5 | 0.752086 |
| *ANAPC4* | | 6 | 2 | 0.752086 |
| *ANO6* | | 6 | 2 | 0.752086 |
| *AOX1* | | 6 | 2 | 0.752086 |
| *AP1G2* | | 6 | 2 | 0.752086 |
| *ARAP3* | | 2 | 5 | 0.752086 |
| *ARHGEF17* | | 6 | 2 | 0.752086 |
| *ARHGEF6* | | 2 | 5 | 0.752086 |
| *ARID5B* | | 6 | 2 | 0.752086 |
| *ATP6V0A4* | | 6 | 2 | 0.752086 |
| *AUP1* | | 2 | 5 | 0.752086 |
| *BAHD1* | | 2 | 5 | 0.752086 |
| *BAIAP3* | | 2 | 5 | 0.752086 |
| *BFAR* | | 2 | 5 | 0.752086 |
| *C16orf62* | | 6 | 2 | 0.752086 |
| *C1S* | | 2 | 5 | 0.752086 |
| *C2CD5* | | 6 | 2 | 0.752086 |
| *C3orf17* | | 2 | 5 | 0.752086 |
| *C8A* | | 2 | 5 | 0.752086 |
| *C9orf3* | | 6 | 2 | 0.752086 |
| *CARD14* | | 6 | 2 | 0.752086 |
| *CASR* | | 2 | 5 | 0.752086 |
| *CATSPERD* | | 6 | 2 | 0.752086 |
| *CCDC136* | | 6 | 2 | 0.752086 |
| *CCDC14* | | 2 | 5 | 0.752086 |
| *CCDC147* | | 2 | 5 | 0.752086 |
| *CCDC41* | | 2 | 5 | 0.752086 |
| *CDH26* | | 6 | 2 | 0.752086 |
| *CDK15* | | 6 | 2 | 0.752086 |
| *CDK8* | | 2 | 5 | 0.752086 |
| *CFB* | | 6 | 2 | 0.752086 |
| *CHAF1A* | | 6 | 2 | 0.752086 |
| *CHRND* | | 2 | 5 | 0.752086 |
| *CLVS2* | | 2 | 5 | 0.752086 |
| *CNTN3* | | 6 | 2 | 0.752086 |
| *COASY* | | 2 | 5 | 0.752086 |
| *COG2* | | 2 | 5 | 0.752086 |
| *COG7* | | 2 | 5 | 0.752086 |
| *COPB2* | | 6 | 2 | 0.752086 |
| *CPE* | | 2 | 5 | 0.752086 |
| *CTNNBL1* | | 2 | 5 | 0.752086 |
| *CYFIP2* | | 2 | 5 | 0.752086 |
| *CYP27B1* | | 6 | 2 | 0.752086 |
| *CYP3A4* | | 6 | 2 | 0.752086 |
| *CYP46A1* | | 2 | 5 | 0.752086 |
| *DDX21* | | 2 | 5 | 0.752086 |
| *DDX26B* | | 2 | 5 | 0.752086 |
| *DHDDS* | | 2 | 5 | 0.752086 |
| *DLGAP4* | | 2 | 5 | 0.752086 |
| *DNAJC3* | | 2 | 5 | 0.752086 |
| *DOCK11* | | 2 | 5 | 0.752086 |
| *E2F7* | | 2 | 5 | 0.752086 |
| *ECI2* | | 6 | 2 | 0.752086 |
| *EEF1A1* | | 2 | 5 | 0.752086 |
| *EGFR* | | 2 | 5 | 0.752086 |
| *EIF3D* | | 2 | 5 | 0.752086 |
| *ELAVL1* | | 2 | 5 | 0.752086 |
| *ELMSAN1* | | 6 | 2 | 0.752086 |
| *ENOX1* | | 2 | 5 | 0.752086 |
| *EPB41L2* | | 2 | 5 | 0.752086 |
| *EPC2* | | 2 | 5 | 0.752086 |
| *EPHX3* | | 2 | 5 | 0.752086 |
| *ERCC3* | | 2 | 5 | 0.752086 |
| *ERCC5* | | 2 | 5 | 0.752086 |
| *ETNPPL* | | 2 | 5 | 0.752086 |
| *EXOC2* | | 6 | 2 | 0.752086 |
| *EYA4* | | 2 | 5 | 0.752086 |
| *F7* | | 6 | 2 | 0.752086 |
| *FADD* | | 2 | 5 | 0.752086 |
| *FAM111B* | | 2 | 5 | 0.752086 |
| *FAM189B* | | 2 | 5 | 0.752086 |
| *FAM49A* | | 2 | 5 | 0.752086 |
| *FARP2* | | 2 | 5 | 0.752086 |
| *FBXO18* | | 2 | 5 | 0.752086 |
| *FMO2* | | 2 | 5 | 0.752086 |
| *FOXE1* | | 6 | 2 | 0.752086 |
| *FRG1B* | | 2 | 5 | 0.752086 |
| *GATA3* | | 2 | 5 | 0.752086 |
| *GP2* | | 6 | 2 | 0.752086 |
| *GPR107* | | 2 | 5 | 0.752086 |
| *GPR161* | | 2 | 5 | 0.752086 |
| *GPR56* | | 2 | 5 | 0.752086 |
| *HINFP* | | 2 | 5 | 0.752086 |
| *HSPA14* | | 2 | 5 | 0.752086 |
| *HUNK* | | 2 | 5 | 0.752086 |
| *ICAM1* | | 2 | 5 | 0.752086 |
| *IGDCC4* | | 2 | 5 | 0.752086 |
| *INTS6* | | 2 | 5 | 0.752086 |
| *IQCC* | | 6 | 2 | 0.752086 |
| *ITGA3* | | 2 | 5 | 0.752086 |
| *ITK* | | 2 | 5 | 0.752086 |
| *KDM1A* | | 2 | 5 | 0.752086 |
| *KIAA0196* | | 6 | 2 | 0.752086 |
| *KIF24* | | 2 | 5 | 0.752086 |
| *KIN* | | 2 | 5 | 0.752086 |
| *LGR6* | | 2 | 5 | 0.752086 |
| *LHCGR* | | 2 | 5 | 0.752086 |
| *LRP10* | | 6 | 2 | 0.752086 |
| *LRP5* | | 6 | 2 | 0.752086 |
| *LRRTM3* | | 2 | 5 | 0.752086 |
| *MAN1B1* | | 6 | 2 | 0.752086 |
| *MAP4K4* | | 2 | 5 | 0.752086 |
| *MASP1* | | 2 | 5 | 0.752086 |
| *MAST2* | | 6 | 2 | 0.752086 |
| *MCC* | | 6 | 2 | 0.752086 |
| *MGAT1* | | 6 | 2 | 0.752086 |
| *MICAL3* | | 2 | 5 | 0.752086 |
| *MNDA* | | 2 | 5 | 0.752086 |
| *MPL* | | 2 | 5 | 0.752086 |
| *MRGPRX1* | | 2 | 5 | 0.752086 |
| *MTF1* | | 6 | 2 | 0.752086 |
| *MTUS2* | | 2 | 5 | 0.752086 |
| *MUC21* | | 2 | 5 | 0.752086 |
| *MYO19* | | 6 | 2 | 0.752086 |
| *MYO6* | | 2 | 5 | 0.752086 |
| *NARS2* | | 2 | 5 | 0.752086 |
| *NEMF* | | 2 | 5 | 0.752086 |
| *NEU3* | | 2 | 5 | 0.752086 |
| *NR2F2* | | 2 | 5 | 0.752086 |
| *NUAK1* | | 2 | 5 | 0.752086 |
| *OPHN1* | | 6 | 2 | 0.752086 |
| *OPTN* | | 6 | 2 | 0.752086 |
| *OR4C46* | | 2 | 5 | 0.752086 |
| *OR8I2* | | 2 | 5 | 0.752086 |
| *OSBPL7* | | 6 | 2 | 0.752086 |
| *PBLD* | | 2 | 5 | 0.752086 |
| *PCK1* | | 6 | 2 | 0.752086 |
| *PDE10A* | | 6 | 2 | 0.752086 |
| *PDE1A* | | 2 | 5 | 0.752086 |
| *PHKA2* | | 2 | 5 | 0.752086 |
| *PITPNM1* | | 2 | 5 | 0.752086 |
| *PITPNM2* | | 2 | 5 | 0.752086 |
| *PLAUR* | | 2 | 5 | 0.752086 |
| *POSTN* | | 2 | 5 | 0.752086 |
| *PRKRIR* | | 2 | 5 | 0.752086 |
| *PRMT7* | | 6 | 2 | 0.752086 |
| *PTCD1* | | 2 | 5 | 0.752086 |
| *PTPRU* | | 2 | 5 | 0.752086 |
| *RALGPS2* | | 2 | 5 | 0.752086 |
| *RECQL4* | | 2 | 5 | 0.752086 |
| *RINT1* | | 2 | 5 | 0.752086 |
| *RLTPR* | | 6 | 2 | 0.752086 |
| *ROBO4* | | 6 | 2 | 0.752086 |
| *RSPRY1* | | 2 | 5 | 0.752086 |
| *RTL1* | | 6 | 2 | 0.752086 |
| *RUNX1T1* | | 2 | 5 | 0.752086 |
| *S1PR1* | | 6 | 2 | 0.752086 |
| *SAMD4B* | | 2 | 5 | 0.752086 |
| *SAMD7* | | 2 | 5 | 0.752086 |
| *SCAI* | | 6 | 2 | 0.752086 |
| *SDHA* | | 6 | 2 | 0.752086 |
| *SEC31A* | | 6 | 2 | 0.752086 |
| *SEMA3F* | | 2 | 5 | 0.752086 |
| *SEMG2* | | 2 | 5 | 0.752086 |
| *1-Sep* | | 6 | 2 | 0.752086 |
| *SESTD1* | | 2 | 5 | 0.752086 |
| *SH2B1* | | 6 | 2 | 0.752086 |
| *SLAIN2* | | 2 | 5 | 0.752086 |
| *SLC27A5* | | 6 | 2 | 0.752086 |
| *SLC4A1AP* | | 2 | 5 | 0.752086 |
| *SLC7A1* | | 6 | 2 | 0.752086 |
| *SLCO1A2* | | 2 | 5 | 0.752086 |
| *SMC3* | | 2 | 5 | 0.752086 |
| *SORCS2* | | 2 | 5 | 0.752086 |
| *SPATA7* | | 6 | 2 | 0.752086 |
| *SPRED2* | | 2 | 5 | 0.752086 |
| *STK10* | | 2 | 5 | 0.752086 |
| *STOX1* | | 2 | 5 | 0.752086 |
| *TBC1D20* | | 2 | 5 | 0.752086 |
| *TBL1XR1* | | 6 | 2 | 0.752086 |
| *TCF12* | | 2 | 5 | 0.752086 |
| *TDRD7* | | 2 | 5 | 0.752086 |
| *TGS1* | | 6 | 2 | 0.752086 |
| *TMC1* | | 2 | 5 | 0.752086 |
| *TMC4* | | 2 | 5 | 0.752086 |
| *TMPRSS7* | | 2 | 5 | 0.752086 |
| *TRPC5* | | 2 | 5 | 0.752086 |
| *TRPM4* | | 2 | 5 | 0.752086 |
| *TRPM8* | | 2 | 5 | 0.752086 |
| *TSEN2* | | 2 | 5 | 0.752086 |
| *UBA52* | | 2 | 5 | 0.752086 |
| *VPS9D1* | | 6 | 2 | 0.752086 |
| *WDPCP* | | 2 | 5 | 0.752086 |
| *WDR20* | | 2 | 5 | 0.752086 |
| *WDR46* | | 2 | 5 | 0.752086 |
| *WDR49* | | 6 | 2 | 0.752086 |
| *YIPF3* | | 2 | 5 | 0.752086 |
| *YTHDC1* | | 6 | 2 | 0.752086 |
| *ZBTB41* | | 2 | 5 | 0.752086 |
| *ZBTB45* | | 2 | 5 | 0.752086 |
| *ZBTB5* | | 2 | 5 | 0.752086 |
| *ZMPSTE24* | | 2 | 5 | 0.752086 |
| *ZNF221* | | 2 | 5 | 0.752086 |
| *ZNF284* | | 2 | 5 | 0.752086 |
| *ZNF492* | | 2 | 5 | 0.752086 |
| *ZNF528* | | 2 | 5 | 0.752086 |
| *ZNF585B* | | 6 | 2 | 0.752086 |
| *ZNF713* | | 2 | 5 | 0.752086 |
| *ZRANB1* | | 6 | 2 | 0.752086 |
| *ABCA6* | | 5 | 9 | 0.758138 |
| *BAP1* | | 5 | 9 | 0.758138 |
| *CAD* | | 5 | 9 | 0.758138 |
| *CDH2* | | 5 | 9 | 0.758138 |
| *COL3A1* | | 5 | 9 | 0.758138 |
| *COL5A3* | | 5 | 9 | 0.758138 |
| *CUL7* | | 5 | 9 | 0.758138 |
| *DPYD* | | 5 | 9 | 0.758138 |
| *EIF4G1* | | 5 | 9 | 0.758138 |
| *IFT172* | | 5 | 9 | 0.758138 |
| *LATS1* | | 5 | 9 | 0.758138 |
| *MAST1* | | 5 | 9 | 0.758138 |
| *MYBPC1* | | 5 | 9 | 0.758138 |
| *NCAM2* | | 5 | 9 | 0.758138 |
| *PARP14* | | 5 | 9 | 0.758138 |
| *QSER1* | | 5 | 9 | 0.758138 |
| *STAB2* | | 5 | 9 | 0.758138 |
| *XIRP1* | | 5 | 9 | 0.758138 |
| *ZIC1* | | 5 | 9 | 0.758138 |
| *ZNF804B* | | 5 | 9 | 0.758138 |
| *A2M* | | 10 | 5 | 0.758138 |
| *FAM179B* | | 10 | 5 | 0.758138 |
| *PROS1* | | 10 | 5 | 0.758138 |
| *PRRC2B* | | 10 | 5 | 0.758138 |
| *PTPRK* | | 10 | 5 | 0.758138 |
| *PTPRM* | | 10 | 5 | 0.758138 |
| *SPG11* | | 10 | 5 | 0.758138 |
| *ZNF99* | | 10 | 5 | 0.758138 |
| *TG* | | 15 | 9 | 0.758138 |
| *COL12A1* | | 9 | 14 | 0.758138 |
| *SF3B1* | | 9 | 14 | 0.758138 |
| *SMARCA4* | | 9 | 14 | 0.758138 |
| *USH2A* | | 15 | 21 | 0.778653 |
| *DNAH11* | | 23 | 30 | 0.782092 |
| *CHD9* | | 10 | 15 | 0.782092 |
| *GON4L* | | 10 | 15 | 0.782092 |
| *SVIL* | | 16 | 10 | 0.782092 |
| *RYR2* | | 34 | 42 | 0.782092 |
| *STAG2* | | 24 | 31 | 0.782092 |
| *CACNA1B* | | 11 | 6 | 0.782092 |
| *CCDC178* | | 11 | 6 | 0.782092 |
| *CD101* | | 11 | 6 | 0.782092 |
| *PAN2* | | 11 | 6 | 0.782092 |
| *SPEF2* | | 11 | 6 | 0.782092 |
| *ZMYND8* | | 11 | 6 | 0.782092 |
| *ACIN1* | | 6 | 10 | 0.782092 |
| *ANKRD26* | | 6 | 10 | 0.782092 |
| *ATN1* | | 6 | 10 | 0.782092 |
| *CACNA1D* | | 6 | 10 | 0.782092 |
| *DLC1* | | 6 | 10 | 0.782092 |
| *FANCD2* | | 6 | 10 | 0.782092 |
| *HECW1* | | 6 | 10 | 0.782092 |
| *ITGAL* | | 6 | 10 | 0.782092 |
| *NEK11* | | 6 | 10 | 0.782092 |
| *NRCAM* | | 6 | 10 | 0.782092 |
| *PIK3C2A* | | 6 | 10 | 0.782092 |
| *PLCH1* | | 6 | 10 | 0.782092 |
| *PTPRC* | | 6 | 10 | 0.782092 |
| *RALGAPB* | | 6 | 10 | 0.782092 |
| *RASAL2* | | 6 | 10 | 0.782092 |
| *SBNO1* | | 6 | 10 | 0.782092 |
| *PHF3* | | 17 | 11 | 0.782092 |
| *POLQ* | | 17 | 11 | 0.782092 |
| *SETD2* | | 17 | 11 | 0.782092 |
| *CDK12* | | 11 | 16 | 0.782092 |
| *KALRN* | | 11 | 16 | 0.782092 |
| *FAT3* | | 25 | 18 | 0.782092 |
| *ABCE1* | | 3 | 6 | 0.782092 |
| *ABCG2* | | 7 | 3 | 0.782092 |
| *ACSM5* | | 7 | 3 | 0.782092 |
| *ADAMTS13* | | 3 | 6 | 0.782092 |
| *ADD2* | | 3 | 6 | 0.782092 |
| *ALDH16A1* | | 3 | 6 | 0.782092 |
| *APBA2* | | 3 | 6 | 0.782092 |
| *ARHGAP33* | | 3 | 6 | 0.782092 |
| *ARHGEF10L* | | 3 | 6 | 0.782092 |
| *ARMC4* | | 3 | 6 | 0.782092 |
| *ATP1A4* | | 3 | 6 | 0.782092 |
| *BTBD11* | | 3 | 6 | 0.782092 |
| *C11orf63* | | 3 | 6 | 0.782092 |
| *C2orf71* | | 3 | 6 | 0.782092 |
| *C6* | | 3 | 6 | 0.782092 |
| *CABIN1* | | 7 | 3 | 0.782092 |
| *CCDC73* | | 3 | 6 | 0.782092 |
| *CCDC88C* | | 7 | 3 | 0.782092 |
| *CDH22* | | 7 | 3 | 0.782092 |
| *CDK5RAP2* | | 3 | 6 | 0.782092 |
| *CEACAM8* | | 3 | 6 | 0.782092 |
| *CENPJ* | | 7 | 3 | 0.782092 |
| *CHEK2* | | 3 | 6 | 0.782092 |
| *CHFR* | | 7 | 3 | 0.782092 |
| *COBL* | | 7 | 3 | 0.782092 |
| *CPAMD8* | | 3 | 6 | 0.782092 |
| *CPSF1* | | 7 | 3 | 0.782092 |
| *CSE1L* | | 3 | 6 | 0.782092 |
| *CXorf22* | | 3 | 6 | 0.782092 |
| *DARS2* | | 7 | 3 | 0.782092 |
| *DMBX1* | | 3 | 6 | 0.782092 |
| *DMTF1* | | 7 | 3 | 0.782092 |
| *DSC3* | | 7 | 3 | 0.782092 |
| *DSPP* | | 3 | 6 | 0.782092 |
| *EIF2B4* | | 3 | 6 | 0.782092 |
| *EPB41L4B* | | 3 | 6 | 0.782092 |
| *FAM120B* | | 3 | 6 | 0.782092 |
| *FAM120C* | | 7 | 3 | 0.782092 |
| *FOXP1* | | 3 | 6 | 0.782092 |
| *FURIN* | | 3 | 6 | 0.782092 |
| *GCFC2* | | 7 | 3 | 0.782092 |
| *GIMAP8* | | 3 | 6 | 0.782092 |
| *GMIP* | | 7 | 3 | 0.782092 |
| *GPD2* | | 3 | 6 | 0.782092 |
| *GPRASP1* | | 7 | 3 | 0.782092 |
| *GRIN2C* | | 7 | 3 | 0.782092 |
| *GTF3C2* | | 3 | 6 | 0.782092 |
| *GTF3C3* | | 3 | 6 | 0.782092 |
| *HCRTR2* | | 7 | 3 | 0.782092 |
| *HDAC6* | | 3 | 6 | 0.782092 |
| *HDAC9* | | 3 | 6 | 0.782092 |
| *HIP1R* | | 3 | 6 | 0.782092 |
| *HMGCS2* | | 3 | 6 | 0.782092 |
| *HSP90B1* | | 7 | 3 | 0.782092 |
| *IFT88* | | 3 | 6 | 0.782092 |
| *IL6ST* | | 3 | 6 | 0.782092 |
| *IPO5* | | 3 | 6 | 0.782092 |
| *IRX5* | | 3 | 6 | 0.782092 |
| *ITGB3* | | 3 | 6 | 0.782092 |
| *KAT2A* | | 3 | 6 | 0.782092 |
| *KCNH6* | | 7 | 3 | 0.782092 |
| *KCNMA1* | | 3 | 6 | 0.782092 |
| *KCNU1* | | 3 | 6 | 0.782092 |
| *KCTD3* | | 3 | 6 | 0.782092 |
| *KHDRBS1* | | 3 | 6 | 0.782092 |
| *KIF19* | | 3 | 6 | 0.782092 |
| *KLHDC2* | | 7 | 3 | 0.782092 |
| *LILRB3* | | 3 | 6 | 0.782092 |
| *LONP1* | | 3 | 6 | 0.782092 |
| *LPP* | | 7 | 3 | 0.782092 |
| *LRRTM1* | | 7 | 3 | 0.782092 |
| *LTBP4* | | 3 | 6 | 0.782092 |
| *MAGEC3* | | 3 | 6 | 0.782092 |
| *MCM10* | | 3 | 6 | 0.782092 |
| *MCM8* | | 3 | 6 | 0.782092 |
| *MEI1* | | 3 | 6 | 0.782092 |
| *MRM1* | | 3 | 6 | 0.782092 |
| *MRPS22* | | 3 | 6 | 0.782092 |
| *MSH4* | | 3 | 6 | 0.782092 |
| *MYCBPAP* | | 3 | 6 | 0.782092 |
| *OR5L2* | | 3 | 6 | 0.782092 |
| *OR5M10* | | 3 | 6 | 0.782092 |
| *OR8H1* | | 3 | 6 | 0.782092 |
| *PABPC4* | | 7 | 3 | 0.782092 |
| *PCDH11Y* | | 3 | 6 | 0.782092 |
| *PCDHA10* | | 7 | 3 | 0.782092 |
| *PCDHGA2* | | 7 | 3 | 0.782092 |
| *PDS5A* | | 3 | 6 | 0.782092 |
| *PHIP* | | 3 | 6 | 0.782092 |
| *PHLPP2* | | 3 | 6 | 0.782092 |
| *PI4KA* | | 3 | 6 | 0.782092 |
| *PIP5K1A* | | 3 | 6 | 0.782092 |
| *PLCB4* | | 3 | 6 | 0.782092 |
| *PMS2* | | 3 | 6 | 0.782092 |
| *POU6F2* | | 3 | 6 | 0.782092 |
| *PRTG* | | 3 | 6 | 0.782092 |
| *PSD* | | 3 | 6 | 0.782092 |
| *PSMC6* | | 3 | 6 | 0.782092 |
| *RAPGEF4* | | 3 | 6 | 0.782092 |
| *RAPH1* | | 7 | 3 | 0.782092 |
| *RASA1* | | 3 | 6 | 0.782092 |
| *RGPD3* | | 7 | 3 | 0.782092 |
| *RNASEL* | | 7 | 3 | 0.782092 |
| *RP11-1055B8.7* | | 3 | 6 | 0.782092 |
| *RPGRIP1* | | 7 | 3 | 0.782092 |
| *RPS6KA3* | | 3 | 6 | 0.782092 |
| *SCN8A* | | 3 | 6 | 0.782092 |
| *SEC24B* | | 7 | 3 | 0.782092 |
| *SEL1L3* | | 7 | 3 | 0.782092 |
| *SH3D19* | | 3 | 6 | 0.782092 |
| *SIGLEC10* | | 3 | 6 | 0.782092 |
| *SLC26A3* | | 7 | 3 | 0.782092 |
| *SLC4A7* | | 3 | 6 | 0.782092 |
| *SLC9C1* | | 3 | 6 | 0.782092 |
| *SLIT1* | | 3 | 6 | 0.782092 |
| *SLTM* | | 7 | 3 | 0.782092 |
| *SMARCA1* | | 7 | 3 | 0.782092 |
| *TACC3* | | 3 | 6 | 0.782092 |
| *TBC1D15* | | 3 | 6 | 0.782092 |
| *TBC1D31* | | 3 | 6 | 0.782092 |
| *TBC1D9B* | | 7 | 3 | 0.782092 |
| *TCHHL1* | | 3 | 6 | 0.782092 |
| *TCOF1* | | 3 | 6 | 0.782092 |
| *TOP3A* | | 7 | 3 | 0.782092 |
| *TRIM71* | | 3 | 6 | 0.782092 |
| *TRMT2A* | | 3 | 6 | 0.782092 |
| *TXK* | | 3 | 6 | 0.782092 |
| *TYK2* | | 3 | 6 | 0.782092 |
| *UBTF* | | 3 | 6 | 0.782092 |
| *VAC14* | | 3 | 6 | 0.782092 |
| *WDR3* | | 7 | 3 | 0.782092 |
| *WDR72* | | 3 | 6 | 0.782092 |
| *WDR90* | | 3 | 6 | 0.782092 |
| *XKR7* | | 3 | 6 | 0.782092 |
| *XPO4* | | 3 | 6 | 0.782092 |
| *XPOT* | | 3 | 6 | 0.782092 |
| *ZCWPW2* | | 3 | 6 | 0.782092 |
| *ZNF441* | | 3 | 6 | 0.782092 |
| *ZNF451* | | 7 | 3 | 0.782092 |
| *ZNF479* | | 7 | 3 | 0.782092 |
| *ZNF544* | | 7 | 3 | 0.782092 |
| *ZNF615* | | 7 | 3 | 0.782092 |
| *ZNF623* | | 3 | 6 | 0.782092 |
| *ZNF827* | | 3 | 6 | 0.782092 |
| *ZSWIM4* | | 3 | 6 | 0.782092 |
| *ZXDC* | | 3 | 6 | 0.782092 |
| *ARID2* | | 12 | 17 | 0.792111 |
| *ASH1L* | | 12 | 17 | 0.792111 |
| *DNAH10* | | 12 | 17 | 0.792111 |
| *HRNR* | | 12 | 17 | 0.792111 |
| *PRKDC* | | 12 | 17 | 0.792111 |
| *ACACB* | | 7 | 11 | 0.799159 |
| *OGDH* | | 7 | 11 | 0.799159 |
| *PCDHA9* | | 7 | 11 | 0.799159 |
| *SLC9A4* | | 7 | 11 | 0.799159 |
| *SPTBN1* | | 7 | 11 | 0.799159 |
| *SSH3* | | 7 | 11 | 0.799159 |
| *WDR96* | | 7 | 11 | 0.799159 |
| *MROH2B* | | 12 | 7 | 0.799159 |
| *MACF1* | | 30 | 37 | 0.808896 |
| *ATR* | | 13 | 18 | 0.820344 |
| *DENND5B* | | 8 | 12 | 0.832082 |
| *DOPEY1* | | 8 | 12 | 0.832082 |
| *KIAA0100* | | 8 | 12 | 0.832082 |
| *ROBO2* | | 8 | 12 | 0.832082 |
| *TICRR* | | 8 | 12 | 0.832082 |
| *ZFYVE26* | | 13 | 8 | 0.832082 |
| *ZNF638* | | 8 | 12 | 0.832082 |
| *WDFY3* | | 20 | 14 | 0.832082 |
| *FRAS1* | | 14 | 19 | 0.832082 |
| *KIAA1109* | | 14 | 19 | 0.832082 |
| *AASS* | | 4 | 7 | 0.832082 |
| *ACE* | | 8 | 4 | 0.832082 |
| *ADAM2* | | 4 | 7 | 0.832082 |
| *ADAMTS14* | | 4 | 7 | 0.832082 |
| *ADD1* | | 4 | 7 | 0.832082 |
| *AGTPBP1* | | 4 | 7 | 0.832082 |
| *ALAS1* | | 8 | 4 | 0.832082 |
| *ANKRD27* | | 4 | 7 | 0.832082 |
| *AQR* | | 4 | 7 | 0.832082 |
| *BZRAP1* | | 8 | 4 | 0.832082 |
| *C10orf137* | | 4 | 7 | 0.832082 |
| *C10orf90* | | 4 | 7 | 0.832082 |
| *C20orf194* | | 4 | 7 | 0.832082 |
| *CCAR1* | | 8 | 4 | 0.832082 |
| *CDON* | | 4 | 7 | 0.832082 |
| *CNST* | | 8 | 4 | 0.832082 |
| *COL15A1* | | 4 | 7 | 0.832082 |
| *COL2A1* | | 8 | 4 | 0.832082 |
| *CRYBG3* | | 4 | 7 | 0.832082 |
| *CSPP1* | | 4 | 7 | 0.832082 |
| *DGKB* | | 8 | 4 | 0.832082 |
| *DNAJC16* | | 4 | 7 | 0.832082 |
| *DOCK1* | | 8 | 4 | 0.832082 |
| *EPRS* | | 4 | 7 | 0.832082 |
| *FAM171A1* | | 8 | 4 | 0.832082 |
| *FBXO43* | | 4 | 7 | 0.832082 |
| *FLT4* | | 4 | 7 | 0.832082 |
| *GPC6* | | 4 | 7 | 0.832082 |
| *GRIP1* | | 4 | 7 | 0.832082 |
| *HELZ2* | | 4 | 7 | 0.832082 |
| *HHIPL2* | | 4 | 7 | 0.832082 |
| *IPO8* | | 4 | 7 | 0.832082 |
| *ITGA2B* | | 8 | 4 | 0.832082 |
| *ITGB8* | | 8 | 4 | 0.832082 |
| *ITIH2* | | 4 | 7 | 0.832082 |
| *KAT6B* | | 4 | 7 | 0.832082 |
| *KPNB1* | | 4 | 7 | 0.832082 |
| *KRIT1* | | 4 | 7 | 0.832082 |
| *LAMB4* | | 8 | 4 | 0.832082 |
| *LARS* | | 8 | 4 | 0.832082 |
| *LRRK1* | | 4 | 7 | 0.832082 |
| *MBD6* | | 4 | 7 | 0.832082 |
| *MDGA2* | | 4 | 7 | 0.832082 |
| *MYO5C* | | 4 | 7 | 0.832082 |
| *NFASC* | | 4 | 7 | 0.832082 |
| *NUPL2* | | 4 | 7 | 0.832082 |
| *PCDHB12* | | 4 | 7 | 0.832082 |
| *PCIF1* | | 4 | 7 | 0.832082 |
| *PDE1C* | | 4 | 7 | 0.832082 |
| *PI4KB* | | 8 | 4 | 0.832082 |
| *PLEKHA4* | | 4 | 7 | 0.832082 |
| *PLEKHG4B* | | 8 | 4 | 0.832082 |
| *POLK* | | 4 | 7 | 0.832082 |
| *POLN* | | 4 | 7 | 0.832082 |
| *POLR2A* | | 4 | 7 | 0.832082 |
| *PRC1* | | 4 | 7 | 0.832082 |
| *PRKCB* | | 8 | 4 | 0.832082 |
| *PTK2* | | 4 | 7 | 0.832082 |
| *PTPN21* | | 8 | 4 | 0.832082 |
| *RSRC2* | | 8 | 4 | 0.832082 |
| *RUSC2* | | 8 | 4 | 0.832082 |
| *SLCO4C1* | | 4 | 7 | 0.832082 |
| *SMG5* | | 8 | 4 | 0.832082 |
| *SPIDR* | | 4 | 7 | 0.832082 |
| *TIMELESS* | | 4 | 7 | 0.832082 |
| *TMEM67* | | 8 | 4 | 0.832082 |
| *TNPO1* | | 8 | 4 | 0.832082 |
| *TNPO3* | | 8 | 4 | 0.832082 |
| *VARS2* | | 8 | 4 | 0.832082 |
| *XPR1* | | 4 | 7 | 0.832082 |
| *YY1AP1* | | 4 | 7 | 0.832082 |
| *ZKSCAN7* | | 4 | 7 | 0.832082 |
| *ZNF217* | | 4 | 7 | 0.832082 |
| *ZNF471* | | 4 | 7 | 0.832082 |
| *ZNF567* | | 8 | 4 | 0.832082 |
| *ZNF93* | | 4 | 7 | 0.832082 |
| *DMD* | | 15 | 20 | 0.836044 |
| *TCHH* | | 15 | 20 | 0.836044 |
| *MKI67* | | 21 | 15 | 0.836044 |
| *ANK1* | | 9 | 13 | 0.848529 |
| *CPD* | | 9 | 13 | 0.848529 |
| *FAM208B* | | 9 | 13 | 0.848529 |
| *FCGBP* | | 9 | 13 | 0.848529 |
| *FLNB* | | 9 | 13 | 0.848529 |
| *JMJD1C* | | 9 | 13 | 0.848529 |
| *LRRIQ1* | | 9 | 13 | 0.848529 |
| *MYH11* | | 9 | 13 | 0.848529 |
| *SALL1* | | 9 | 13 | 0.848529 |
| *INO80* | | 14 | 9 | 0.848529 |
| *ZZEF1* | | 14 | 9 | 0.848529 |
| *ERCC2* | | 22 | 16 | 0.855119 |
| *SACS* | | 22 | 16 | 0.855119 |
| *LRP2* | | 17 | 22 | 0.856224 |
| *ASXL1* | | 10 | 14 | 0.856224 |
| *CIT* | | 10 | 14 | 0.856224 |
| *MGA* | | 10 | 14 | 0.856224 |
| *PARD3* | | 10 | 14 | 0.856224 |
| *TENM2* | | 10 | 14 | 0.856224 |
| *ZFP36L1* | | 10 | 14 | 0.856224 |
| *ABCC11* | | 5 | 8 | 0.856224 |
| *ADAM10* | | 5 | 8 | 0.856224 |
| *ATF7IP* | | 5 | 8 | 0.856224 |
| *ATP13A2* | | 5 | 8 | 0.856224 |
| *CARD6* | | 5 | 8 | 0.856224 |
| *CCDC30* | | 5 | 8 | 0.856224 |
| *CD1E* | | 5 | 8 | 0.856224 |
| *CNTNAP5* | | 5 | 8 | 0.856224 |
| *EHMT2* | | 5 | 8 | 0.856224 |
| *FBXL7* | | 5 | 8 | 0.856224 |
| *FCHO1* | | 5 | 8 | 0.856224 |
| *FOXA1* | | 5 | 8 | 0.856224 |
| *HDLBP* | | 5 | 8 | 0.856224 |
| *KIAA0319L* | | 5 | 8 | 0.856224 |
| *LEO1* | | 5 | 8 | 0.856224 |
| *LIPE* | | 5 | 8 | 0.856224 |
| *LRP12* | | 5 | 8 | 0.856224 |
| *LRRC41* | | 5 | 8 | 0.856224 |
| *MME* | | 5 | 8 | 0.856224 |
| *NID1* | | 5 | 8 | 0.856224 |
| *NRD1* | | 5 | 8 | 0.856224 |
| *OPA1* | | 5 | 8 | 0.856224 |
| *PCDHB11* | | 5 | 8 | 0.856224 |
| *PKD1* | | 5 | 8 | 0.856224 |
| *POT1* | | 5 | 8 | 0.856224 |
| *PPP4R1* | | 5 | 8 | 0.856224 |
| *QRICH2* | | 5 | 8 | 0.856224 |
| *RNF123* | | 5 | 8 | 0.856224 |
| *SLC12A7* | | 5 | 8 | 0.856224 |
| *SLC44A2* | | 5 | 8 | 0.856224 |
| *SPTBN2* | | 5 | 8 | 0.856224 |
| *TNS3* | | 5 | 8 | 0.856224 |
| *TTC18* | | 5 | 8 | 0.856224 |
| *USP29* | | 5 | 8 | 0.856224 |
| *USP54* | | 5 | 8 | 0.856224 |
| *USPL1* | | 5 | 8 | 0.856224 |
| *ZCCHC8* | | 5 | 8 | 0.856224 |
| *ZNF106* | | 5 | 8 | 0.856224 |
| *ABCC3* | | 9 | 5 | 0.856224 |
| *AHCTF1* | | 9 | 5 | 0.856224 |
| *CD163* | | 9 | 5 | 0.856224 |
| *CHD3* | | 9 | 5 | 0.856224 |
| *CR2* | | 9 | 5 | 0.856224 |
| *EGFLAM* | | 9 | 5 | 0.856224 |
| *FNDC1* | | 9 | 5 | 0.856224 |
| *GRIN2A* | | 9 | 5 | 0.856224 |
| *MAN2A1* | | 9 | 5 | 0.856224 |
| *NAA15* | | 9 | 5 | 0.856224 |
| *NUMA1* | | 9 | 5 | 0.856224 |
| *PREX1* | | 9 | 5 | 0.856224 |
| *SYTL2* | | 9 | 5 | 0.856224 |
| *ZFPM2* | | 9 | 5 | 0.856224 |
| *ACACA* | | 11 | 15 | 0.856224 |
| *ANKRD11* | | 11 | 15 | 0.856224 |
| *CDKN2A* | | 16 | 11 | 0.856224 |
| *CHD2* | | 16 | 11 | 0.856224 |
| *COL6A3* | | 12 | 16 | 0.856224 |
| *CSMD2* | | 17 | 12 | 0.856224 |
| *MUC16* | | 54 | 61 | 0.856224 |
| *DEPDC5* | | 10 | 6 | 0.856224 |
| *GOLGA2* | | 10 | 6 | 0.856224 |
| *NDC80* | | 10 | 6 | 0.856224 |
| *PTCH1* | | 10 | 6 | 0.856224 |
| *RNF17* | | 10 | 6 | 0.856224 |
| *RTTN* | | 10 | 6 | 0.856224 |
| *SLX4* | | 10 | 6 | 0.856224 |
| *SMC4* | | 10 | 6 | 0.856224 |
| *ZC3H13* | | 10 | 6 | 0.856224 |
| *ADCY10* | | 6 | 9 | 0.856224 |
| *ATP8B4* | | 6 | 9 | 0.856224 |
| *CDH18* | | 6 | 9 | 0.856224 |
| *DLG5* | | 6 | 9 | 0.856224 |
| *EPB41L3* | | 6 | 9 | 0.856224 |
| *GBF1* | | 6 | 9 | 0.856224 |
| *ITSN1* | | 6 | 9 | 0.856224 |
| *KIAA1551* | | 6 | 9 | 0.856224 |
| *MYH6* | | 6 | 9 | 0.856224 |
| *MYOM1* | | 6 | 9 | 0.856224 |
| *NLRP9* | | 6 | 9 | 0.856224 |
| *OSBPL3* | | 6 | 9 | 0.856224 |
| *PCDHA2* | | 6 | 9 | 0.856224 |
| *PCDHB6* | | 6 | 9 | 0.856224 |
| *PCNXL4* | | 6 | 9 | 0.856224 |
| *RAB11FIP1* | | 6 | 9 | 0.856224 |
| *RPS6KC1* | | 6 | 9 | 0.856224 |
| *RREB1* | | 6 | 9 | 0.856224 |
| *SCN5A* | | 6 | 9 | 0.856224 |
| *SYCP2* | | 6 | 9 | 0.856224 |
| *TBC1D8B* | | 6 | 9 | 0.856224 |
| *TP63* | | 6 | 9 | 0.856224 |
| *TRPC4* | | 6 | 9 | 0.856224 |
| *VIT* | | 6 | 9 | 0.856224 |
| *ADAMTSL4* | | 5 | 2 | 0.856224 |
| *AFG3L2* | | 5 | 2 | 0.856224 |
| *ALB* | | 5 | 2 | 0.856224 |
| *AMACR* | | 5 | 2 | 0.856224 |
| *ARHGAP39* | | 5 | 2 | 0.856224 |
| *ARHGEF10* | | 5 | 2 | 0.856224 |
| *ARHGEF28* | | 5 | 2 | 0.856224 |
| *ARMCX2* | | 5 | 2 | 0.856224 |
| *ATG4C* | | 5 | 2 | 0.856224 |
| *ATP7A* | | 5 | 2 | 0.856224 |
| *BICD2* | | 5 | 2 | 0.856224 |
| *BNC1* | | 5 | 2 | 0.856224 |
| *C1orf141* | | 5 | 2 | 0.856224 |
| *CAPN3* | | 5 | 2 | 0.856224 |
| *CASP5* | | 5 | 2 | 0.856224 |
| *CATSPER1* | | 5 | 2 | 0.856224 |
| *CATSPER2* | | 5 | 2 | 0.856224 |
| *CCDC33* | | 5 | 2 | 0.856224 |
| *CDK5RAP1* | | 5 | 2 | 0.856224 |
| *CDK7* | | 5 | 2 | 0.856224 |
| *CORO2B* | | 5 | 2 | 0.856224 |
| *CPA6* | | 5 | 2 | 0.856224 |
| *CPN2* | | 5 | 2 | 0.856224 |
| *CYFIP1* | | 5 | 2 | 0.856224 |
| *DACT1* | | 5 | 2 | 0.856224 |
| *DOCK5* | | 5 | 2 | 0.856224 |
| *DSTYK* | | 5 | 2 | 0.856224 |
| *DUSP27* | | 5 | 2 | 0.856224 |
| *ECT2L* | | 5 | 2 | 0.856224 |
| *FAM13B* | | 5 | 2 | 0.856224 |
| *FAM208A* | | 5 | 2 | 0.856224 |
| *FASTKD1* | | 5 | 2 | 0.856224 |
| *FBXO28* | | 5 | 2 | 0.856224 |
| *FILIP1L* | | 5 | 2 | 0.856224 |
| *FSTL4* | | 5 | 2 | 0.856224 |
| *HCAR2* | | 5 | 2 | 0.856224 |
| *HIST1H2BI* | | 5 | 2 | 0.856224 |
| *HNF1A* | | 5 | 2 | 0.856224 |
| *HOMEZ* | | 5 | 2 | 0.856224 |
| *IL17RC* | | 5 | 2 | 0.856224 |
| *ITIH6* | | 5 | 2 | 0.856224 |
| *KCNF1* | | 5 | 2 | 0.856224 |
| *KEAP1* | | 5 | 2 | 0.856224 |
| *KIF3C* | | 5 | 2 | 0.856224 |
| *LAX1* | | 5 | 2 | 0.856224 |
| *LMOD2* | | 5 | 2 | 0.856224 |
| *LRRC49* | | 5 | 2 | 0.856224 |
| *LTF* | | 5 | 2 | 0.856224 |
| *MALT1* | | 5 | 2 | 0.856224 |
| *MAP3K15* | | 5 | 2 | 0.856224 |
| *MARCO* | | 5 | 2 | 0.856224 |
| *MB21D2* | | 5 | 2 | 0.856224 |
| *MBTD1* | | 5 | 2 | 0.856224 |
| *MED15* | | 5 | 2 | 0.856224 |
| *MFSD8* | | 5 | 2 | 0.856224 |
| *MIB2* | | 5 | 2 | 0.856224 |
| *MID1* | | 5 | 2 | 0.856224 |
| *MN1* | | 5 | 2 | 0.856224 |
| *MTMR12* | | 5 | 2 | 0.856224 |
| *MZF1* | | 5 | 2 | 0.856224 |
| *NHLRC2* | | 5 | 2 | 0.856224 |
| *NPAS2* | | 5 | 2 | 0.856224 |
| *NPR1* | | 5 | 2 | 0.856224 |
| *NXF3* | | 5 | 2 | 0.856224 |
| *OR2G2* | | 5 | 2 | 0.856224 |
| *PCDHA11* | | 5 | 2 | 0.856224 |
| *PDGFRB* | | 5 | 2 | 0.856224 |
| *PDXDC1* | | 5 | 2 | 0.856224 |
| *PHTF1* | | 5 | 2 | 0.856224 |
| *PICALM* | | 5 | 2 | 0.856224 |
| *POU4F2* | | 5 | 2 | 0.856224 |
| *PPM1B* | | 5 | 2 | 0.856224 |
| *PSG6* | | 5 | 2 | 0.856224 |
| *PTRF* | | 5 | 2 | 0.856224 |
| *RTN4* | | 5 | 2 | 0.856224 |
| *SDPR* | | 5 | 2 | 0.856224 |
| *SEMA5A* | | 5 | 2 | 0.856224 |
| *SERPINI2* | | 5 | 2 | 0.856224 |
| *SGSM2* | | 5 | 2 | 0.856224 |
| *SLC17A6* | | 5 | 2 | 0.856224 |
| *SLC2A2* | | 5 | 2 | 0.856224 |
| *SLC35G3* | | 5 | 2 | 0.856224 |
| *SLC4A3* | | 5 | 2 | 0.856224 |
| *SMPD1* | | 5 | 2 | 0.856224 |
| *STAC3* | | 5 | 2 | 0.856224 |
| *STK3* | | 5 | 2 | 0.856224 |
| *SYT15* | | 5 | 2 | 0.856224 |
| *TAF6* | | 5 | 2 | 0.856224 |
| *TFPI2* | | 5 | 2 | 0.856224 |
| *TLR9* | | 5 | 2 | 0.856224 |
| *TMEM245* | | 5 | 2 | 0.856224 |
| *TOP3B* | | 5 | 2 | 0.856224 |
| *TRHR* | | 5 | 2 | 0.856224 |
| *TRIP13* | | 5 | 2 | 0.856224 |
| *TSKS* | | 5 | 2 | 0.856224 |
| *TUBB6* | | 5 | 2 | 0.856224 |
| *USP43* | | 5 | 2 | 0.856224 |
| *UTP14C* | | 5 | 2 | 0.856224 |
| *VPS52* | | 5 | 2 | 0.856224 |
| *WWC1* | | 5 | 2 | 0.856224 |
| *ZFP28* | | 5 | 2 | 0.856224 |
| *ZNF257* | | 5 | 2 | 0.856224 |
| *ZNF383* | | 5 | 2 | 0.856224 |
| *ZNF484* | | 5 | 2 | 0.856224 |
| *BPTF* | | 13 | 17 | 0.856224 |
| *TENM3* | | 14 | 18 | 0.856224 |
| *PLEC* | | 19 | 14 | 0.856224 |
| *ABCC9* | | 11 | 7 | 0.856224 |
| *CDH4* | | 11 | 7 | 0.856224 |
| *GLI3* | | 11 | 7 | 0.856224 |
| *MYOM3* | | 11 | 7 | 0.856224 |
| *PEAK1* | | 11 | 7 | 0.856224 |
| *PLCB1* | | 11 | 7 | 0.856224 |
| *TNS1* | | 11 | 7 | 0.856224 |
| *VWF* | | 11 | 7 | 0.856224 |
| *AGL* | | 7 | 10 | 0.856224 |
| *CALD1* | | 7 | 10 | 0.856224 |
| *CTTNBP2* | | 7 | 10 | 0.856224 |
| *DENND4A* | | 7 | 10 | 0.856224 |
| *GLI2* | | 7 | 10 | 0.856224 |
| *HEATR5B* | | 7 | 10 | 0.856224 |
| *HFM1* | | 7 | 10 | 0.856224 |
| *MED1* | | 7 | 10 | 0.856224 |
| *PHF20* | | 7 | 10 | 0.856224 |
| *PXDNL* | | 7 | 10 | 0.856224 |
| *SCRIB* | | 7 | 10 | 0.856224 |
| *SEC16A* | | 7 | 10 | 0.856224 |
| *MDN1* | | 20 | 15 | 0.856224 |
| *TSC1* | | 15 | 19 | 0.856224 |
| *ARID1A* | | 53 | 46 | 0.856224 |
| *CEP192* | | 12 | 8 | 0.856224 |
| *ITPR1* | | 12 | 8 | 0.856224 |
| *MPDZ* | | 12 | 8 | 0.856224 |
| *MYH7* | | 12 | 8 | 0.856224 |
| *SI* | | 12 | 8 | 0.856224 |
| *TRPS1* | | 12 | 8 | 0.856224 |
| *ABCC5* | | 8 | 11 | 0.856224 |
| *ATP10A* | | 8 | 11 | 0.856224 |
| *CENPF* | | 8 | 11 | 0.856224 |
| *GCN1L1* | | 8 | 11 | 0.856224 |
| *GPR158* | | 8 | 11 | 0.856224 |
| *GRM1* | | 8 | 11 | 0.856224 |
| *IMPG2* | | 8 | 11 | 0.856224 |
| *MYH7B* | | 8 | 11 | 0.856224 |
| *RC3H1* | | 8 | 11 | 0.856224 |
| *TPTE* | | 8 | 11 | 0.856224 |
| *FREM2* | | 17 | 21 | 0.856224 |
| *ADAM21* | | 6 | 3 | 0.856224 |
| *ADARB2* | | 6 | 3 | 0.856224 |
| *ADAT1* | | 6 | 3 | 0.856224 |
| *ANKZF1* | | 6 | 3 | 0.856224 |
| *BARD1* | | 6 | 3 | 0.856224 |
| *C11orf82* | | 6 | 3 | 0.856224 |
| *C3orf67* | | 6 | 3 | 0.856224 |
| *CEP164* | | 6 | 3 | 0.856224 |
| *COG4* | | 6 | 3 | 0.856224 |
| *COL17A1* | | 6 | 3 | 0.856224 |
| *CTR9* | | 6 | 3 | 0.856224 |
| *CTTN* | | 6 | 3 | 0.856224 |
| *DAAM2* | | 6 | 3 | 0.856224 |
| *DZIP3* | | 6 | 3 | 0.856224 |
| *FAM65A* | | 6 | 3 | 0.856224 |
| *FGFR2* | | 6 | 3 | 0.856224 |
| *FNIP2* | | 6 | 3 | 0.856224 |
| *GIGYF2* | | 6 | 3 | 0.856224 |
| *HIPK1* | | 6 | 3 | 0.856224 |
| *HIRA* | | 6 | 3 | 0.856224 |
| *HK3* | | 6 | 3 | 0.856224 |
| *KCND2* | | 6 | 3 | 0.856224 |
| *KCTD19* | | 6 | 3 | 0.856224 |
| *KIF6* | | 6 | 3 | 0.856224 |
| *KLHL41* | | 6 | 3 | 0.856224 |
| *KPNA1* | | 6 | 3 | 0.856224 |
| *LRIG2* | | 6 | 3 | 0.856224 |
| *LRRC31* | | 6 | 3 | 0.856224 |
| *MAP3K6* | | 6 | 3 | 0.856224 |
| *MED23* | | 6 | 3 | 0.856224 |
| *MGEA5* | | 6 | 3 | 0.856224 |
| *MMS22L* | | 6 | 3 | 0.856224 |
| *MUT* | | 6 | 3 | 0.856224 |
| *MYLK3* | | 6 | 3 | 0.856224 |
| *NCAPD3* | | 6 | 3 | 0.856224 |
| *NR1D2* | | 6 | 3 | 0.856224 |
| *NRP1* | | 6 | 3 | 0.856224 |
| *NSUN6* | | 6 | 3 | 0.856224 |
| *OR4A47* | | 6 | 3 | 0.856224 |
| *OSBPL8* | | 6 | 3 | 0.856224 |
| *PAX3* | | 6 | 3 | 0.856224 |
| *PCDHGC3* | | 6 | 3 | 0.856224 |
| *PCSK6* | | 6 | 3 | 0.856224 |
| *PEX1* | | 6 | 3 | 0.856224 |
| *PHF1* | | 6 | 3 | 0.856224 |
| *PIEZO2* | | 6 | 3 | 0.856224 |
| *PIK3R4* | | 6 | 3 | 0.856224 |
| *POLD1* | | 6 | 3 | 0.856224 |
| *PRICKLE1* | | 6 | 3 | 0.856224 |
| *RBM5* | | 6 | 3 | 0.856224 |
| *REV1* | | 6 | 3 | 0.856224 |
| *REXO1* | | 6 | 3 | 0.856224 |
| *RSPH6A* | | 6 | 3 | 0.856224 |
| *SAGE1* | | 6 | 3 | 0.856224 |
| *SDCCAG8* | | 6 | 3 | 0.856224 |
| *SLITRK3* | | 6 | 3 | 0.856224 |
| *SYT4* | | 6 | 3 | 0.856224 |
| *TCF25* | | 6 | 3 | 0.856224 |
| *TFAP2A* | | 6 | 3 | 0.856224 |
| *THOC5* | | 6 | 3 | 0.856224 |
| *TMC5* | | 6 | 3 | 0.856224 |
| *TOMM70A* | | 6 | 3 | 0.856224 |
| *UGP2* | | 6 | 3 | 0.856224 |
| *VEZT* | | 6 | 3 | 0.856224 |
| *ZHX3* | | 6 | 3 | 0.856224 |
| *ZKSCAN5* | | 6 | 3 | 0.856224 |
| *ZNF493* | | 6 | 3 | 0.856224 |
| *ZNF555* | | 6 | 3 | 0.856224 |
| *ZNF671* | | 6 | 3 | 0.856224 |
| *ZSCAN20* | | 6 | 3 | 0.856224 |
| *AC004381.6* | | 3 | 5 | 0.856224 |
| *ADAMTS5* | | 3 | 5 | 0.856224 |
| *AKAP1* | | 3 | 5 | 0.856224 |
| *ANKS1B* | | 3 | 5 | 0.856224 |
| *APLP2* | | 3 | 5 | 0.856224 |
| *ARHGAP17* | | 3 | 5 | 0.856224 |
| *ASIC5* | | 3 | 5 | 0.856224 |
| *ATF7IP2* | | 3 | 5 | 0.856224 |
| *ATP13A5* | | 3 | 5 | 0.856224 |
| *BRPF3* | | 3 | 5 | 0.856224 |
| *BRWD3* | | 3 | 5 | 0.856224 |
| *BTN3A3* | | 3 | 5 | 0.856224 |
| *C12orf40* | | 3 | 5 | 0.856224 |
| *C1orf116* | | 3 | 5 | 0.856224 |
| *C2CD2* | | 3 | 5 | 0.856224 |
| *CACNB2* | | 3 | 5 | 0.856224 |
| *CASC3* | | 3 | 5 | 0.856224 |
| *CASKIN2* | | 3 | 5 | 0.856224 |
| *CC2D1B* | | 3 | 5 | 0.856224 |
| *CCDC102B* | | 3 | 5 | 0.856224 |
| *CCDC110* | | 3 | 5 | 0.856224 |
| *CDC14B* | | 3 | 5 | 0.856224 |
| *CDC5L* | | 3 | 5 | 0.856224 |
| *CEL* | | 3 | 5 | 0.856224 |
| *CELF2* | | 3 | 5 | 0.856224 |
| *CEP68* | | 3 | 5 | 0.856224 |
| *CLCN2* | | 3 | 5 | 0.856224 |
| *CLN3* | | 3 | 5 | 0.856224 |
| *COG1* | | 3 | 5 | 0.856224 |
| *CSPG5* | | 3 | 5 | 0.856224 |
| *CSRP2BP* | | 3 | 5 | 0.856224 |
| *CYP2B6* | | 3 | 5 | 0.856224 |
| *CYP4A22* | | 3 | 5 | 0.856224 |
| *DCAF12L1* | | 3 | 5 | 0.856224 |
| *DDB2* | | 3 | 5 | 0.856224 |
| *DDX10* | | 3 | 5 | 0.856224 |
| *DENND3* | | 3 | 5 | 0.856224 |
| *DGKE* | | 3 | 5 | 0.856224 |
| *DLD* | | 3 | 5 | 0.856224 |
| *DNAAF2* | | 3 | 5 | 0.856224 |
| *DVL2* | | 3 | 5 | 0.856224 |
| *EDEM3* | | 3 | 5 | 0.856224 |
| *EFEMP1* | | 3 | 5 | 0.856224 |
| *EIF2AK3* | | 3 | 5 | 0.856224 |
| *ELP2* | | 3 | 5 | 0.856224 |
| *F13B* | | 3 | 5 | 0.856224 |
| *FAM83B* | | 3 | 5 | 0.856224 |
| *FBLN2* | | 3 | 5 | 0.856224 |
| *FCRL3* | | 3 | 5 | 0.856224 |
| *FCRL4* | | 3 | 5 | 0.856224 |
| *FHOD3* | | 3 | 5 | 0.856224 |
| *FLAD1* | | 3 | 5 | 0.856224 |
| *FRMPD2* | | 3 | 5 | 0.856224 |
| *FUK* | | 3 | 5 | 0.856224 |
| *GABRA6* | | 3 | 5 | 0.856224 |
| *GABRB1* | | 3 | 5 | 0.856224 |
| *GALNT2* | | 3 | 5 | 0.856224 |
| *GDPD4* | | 3 | 5 | 0.856224 |
| *GFM2* | | 3 | 5 | 0.856224 |
| *GPR110* | | 3 | 5 | 0.856224 |
| *GRM6* | | 3 | 5 | 0.856224 |
| *GTF2IRD1* | | 3 | 5 | 0.856224 |
| *HEATR4* | | 3 | 5 | 0.856224 |
| *HECTD2* | | 3 | 5 | 0.856224 |
| *HELB* | | 3 | 5 | 0.856224 |
| *HIRIP3* | | 3 | 5 | 0.856224 |
| *IARS2* | | 3 | 5 | 0.856224 |
| *IFIH1* | | 3 | 5 | 0.856224 |
| *IGSF22* | | 3 | 5 | 0.856224 |
| *IL21R* | | 3 | 5 | 0.856224 |
| *IMPDH2* | | 3 | 5 | 0.856224 |
| *IREB2* | | 3 | 5 | 0.856224 |
| *IRS1* | | 3 | 5 | 0.856224 |
| *ITGB6* | | 3 | 5 | 0.856224 |
| *JAK2* | | 3 | 5 | 0.856224 |
| *KCNA1* | | 3 | 5 | 0.856224 |
| *KIAA1244* | | 3 | 5 | 0.856224 |
| *KIF5B* | | 3 | 5 | 0.856224 |
| *KLHDC7A* | | 3 | 5 | 0.856224 |
| *KLHL12* | | 3 | 5 | 0.856224 |
| *KLHL2* | | 3 | 5 | 0.856224 |
| *KSR2* | | 3 | 5 | 0.856224 |
| *LARP4* | | 3 | 5 | 0.856224 |
| *LEPR* | | 3 | 5 | 0.856224 |
| *LMLN* | | 3 | 5 | 0.856224 |
| *LPPR5* | | 3 | 5 | 0.856224 |
| *LRIT1* | | 3 | 5 | 0.856224 |
| *LRRC4C* | | 3 | 5 | 0.856224 |
| *LRRIQ3* | | 3 | 5 | 0.856224 |
| *M1AP* | | 3 | 5 | 0.856224 |
| *MCAM* | | 3 | 5 | 0.856224 |
| *MCM6* | | 3 | 5 | 0.856224 |
| *MCM9* | | 3 | 5 | 0.856224 |
| *MCPH1* | | 3 | 5 | 0.856224 |
| *MEPE* | | 3 | 5 | 0.856224 |
| *METTL2A* | | 3 | 5 | 0.856224 |
| *MLH1* | | 3 | 5 | 0.856224 |
| *MMP2* | | 3 | 5 | 0.856224 |
| *MORC3* | | 3 | 5 | 0.856224 |
| *MS4A14* | | 3 | 5 | 0.856224 |
| *NCAM1* | | 3 | 5 | 0.856224 |
| *NKAPL* | | 3 | 5 | 0.856224 |
| *NLRP11* | | 3 | 5 | 0.856224 |
| *NLRP13* | | 3 | 5 | 0.856224 |
| *NRDE2* | | 3 | 5 | 0.856224 |
| *NUP85* | | 3 | 5 | 0.856224 |
| *OAS3* | | 3 | 5 | 0.856224 |
| *OR5A1* | | 3 | 5 | 0.856224 |
| *OTUD7A* | | 3 | 5 | 0.856224 |
| *PABPC3* | | 3 | 5 | 0.856224 |
| *PAXIP1* | | 3 | 5 | 0.856224 |
| *PCDHA3* | | 3 | 5 | 0.856224 |
| *PDSS1* | | 3 | 5 | 0.856224 |
| *PDSS2* | | 3 | 5 | 0.856224 |
| *PGM5* | | 3 | 5 | 0.856224 |
| *PMS1* | | 3 | 5 | 0.856224 |
| *PNLIPRP1* | | 3 | 5 | 0.856224 |
| *PNPLA6* | | 3 | 5 | 0.856224 |
| *POLA1* | | 3 | 5 | 0.856224 |
| *POP1* | | 3 | 5 | 0.856224 |
| *PRAMEF1* | | 3 | 5 | 0.856224 |
| *PRKAA1* | | 3 | 5 | 0.856224 |
| *PRRT3* | | 3 | 5 | 0.856224 |
| *PRUNE* | | 3 | 5 | 0.856224 |
| *PTGER2* | | 3 | 5 | 0.856224 |
| *PTPRN2* | | 3 | 5 | 0.856224 |
| *PTPRO* | | 3 | 5 | 0.856224 |
| *PYGB* | | 3 | 5 | 0.856224 |
| *RFC4* | | 3 | 5 | 0.856224 |
| *RIOK2* | | 3 | 5 | 0.856224 |
| *RSC1A1* | | 3 | 5 | 0.856224 |
| *RSPH4A* | | 3 | 5 | 0.856224 |
| *SBF1* | | 3 | 5 | 0.856224 |
| *SEC14L1* | | 3 | 5 | 0.856224 |
| *SECISBP2L* | | 3 | 5 | 0.856224 |
| *SEMA3G* | | 3 | 5 | 0.856224 |
| *SERPINA12* | | 3 | 5 | 0.856224 |
| *SFMBT2* | | 3 | 5 | 0.856224 |
| *SLC10A2* | | 3 | 5 | 0.856224 |
| *SLC12A6* | | 3 | 5 | 0.856224 |
| *SLC6A1* | | 3 | 5 | 0.856224 |
| *SLC6A19* | | 3 | 5 | 0.856224 |
| *SLC6A9* | | 3 | 5 | 0.856224 |
| *SNX33* | | 3 | 5 | 0.856224 |
| *SOS2* | | 3 | 5 | 0.856224 |
| *SP4* | | 3 | 5 | 0.856224 |
| *SPARCL1* | | 3 | 5 | 0.856224 |
| *SPHK2* | | 3 | 5 | 0.856224 |
| *SRCIN1* | | 3 | 5 | 0.856224 |
| *ST14* | | 3 | 5 | 0.856224 |
| *STAT6* | | 3 | 5 | 0.856224 |
| *STAU1* | | 3 | 5 | 0.856224 |
| *SYCP1* | | 3 | 5 | 0.856224 |
| *TDRD5* | | 3 | 5 | 0.856224 |
| *TEX26* | | 3 | 5 | 0.856224 |
| *THSD1* | | 3 | 5 | 0.856224 |
| *TIGD2* | | 3 | 5 | 0.856224 |
| *TMEM206* | | 3 | 5 | 0.856224 |
| *TMPRSS9* | | 3 | 5 | 0.856224 |
| *TRIM36* | | 3 | 5 | 0.856224 |
| *TRMT1L* | | 3 | 5 | 0.856224 |
| *TTC40* | | 3 | 5 | 0.856224 |
| *TTC7A* | | 3 | 5 | 0.856224 |
| *UBE4A* | | 3 | 5 | 0.856224 |
| *UBN1* | | 3 | 5 | 0.856224 |
| *VAV2* | | 3 | 5 | 0.856224 |
| *VILL* | | 3 | 5 | 0.856224 |
| *VPS11* | | 3 | 5 | 0.856224 |
| *VRTN* | | 3 | 5 | 0.856224 |
| *WRN* | | 3 | 5 | 0.856224 |
| *ZBTB17* | | 3 | 5 | 0.856224 |
| *ZBTB32* | | 3 | 5 | 0.856224 |
| *ZIK1* | | 3 | 5 | 0.856224 |
| *ZNF197* | | 3 | 5 | 0.856224 |
| *ZNF341* | | 3 | 5 | 0.856224 |
| *ZNF468* | | 3 | 5 | 0.856224 |
| *ZNF570* | | 3 | 5 | 0.856224 |
| *ZNF582* | | 3 | 5 | 0.856224 |
| *ZNF620* | | 3 | 5 | 0.856224 |
| *ZNF676* | | 3 | 5 | 0.856224 |
| *ZNF781* | | 3 | 5 | 0.856224 |
| *ZNF823* | | 3 | 5 | 0.856224 |
| *ZP2* | | 3 | 5 | 0.856224 |
| *COL22A1* | | 9 | 12 | 0.856967 |
| *DSCAM* | | 9 | 12 | 0.856967 |
| *KDM3B* | | 9 | 12 | 0.856967 |
| *SCAF4* | | 9 | 12 | 0.856967 |
| *TECTA* | | 9 | 12 | 0.856967 |
| *ZDBF2* | | 9 | 12 | 0.856967 |
| *ZNF208* | | 9 | 12 | 0.856967 |
| *ADCY2* | | 13 | 9 | 0.856967 |
| *PCDH9* | | 13 | 9 | 0.856967 |
| *ZNF804A* | | 13 | 9 | 0.856967 |
| *ANK2* | | 19 | 23 | 0.856967 |
| *ARHGAP32* | | 14 | 10 | 0.856967 |
| *RXRA* | | 14 | 10 | 0.856967 |
| *CEP350* | | 10 | 13 | 0.856967 |
| *CNTNAP2* | | 10 | 13 | 0.856967 |
| *KLF5* | | 10 | 13 | 0.856967 |
| *TAF1L* | | 10 | 13 | 0.856967 |
| *SYNE1* | | 44 | 38 | 0.856967 |
| *LRP1B* | | 22 | 26 | 0.856967 |
| *ABCA5* | | 4 | 6 | 0.856967 |
| *ABCC1* | | 4 | 6 | 0.856967 |
| *ABCC8* | | 4 | 6 | 0.856967 |
| *ACAD10* | | 4 | 6 | 0.856967 |
| *ADAMTS10* | | 4 | 6 | 0.856967 |
| *AGGF1* | | 4 | 6 | 0.856967 |
| *AKNAD1* | | 4 | 6 | 0.856967 |
| *AMOT* | | 4 | 6 | 0.856967 |
| *AMPD1* | | 4 | 6 | 0.856967 |
| *ANO5* | | 4 | 6 | 0.856967 |
| *ARAP1* | | 4 | 6 | 0.856967 |
| *ARIH2* | | 4 | 6 | 0.856967 |
| *ASUN* | | 4 | 6 | 0.856967 |
| *ATP7B* | | 4 | 6 | 0.856967 |
| *BMS1* | | 4 | 6 | 0.856967 |
| *BTBD7* | | 4 | 6 | 0.856967 |
| *CACNA1S* | | 4 | 6 | 0.856967 |
| *CATSPERB* | | 4 | 6 | 0.856967 |
| *CCDC151* | | 4 | 6 | 0.856967 |
| *CCDC158* | | 4 | 6 | 0.856967 |
| *CDH9* | | 4 | 6 | 0.856967 |
| *CEP89* | | 4 | 6 | 0.856967 |
| *CLEC16A* | | 4 | 6 | 0.856967 |
| *CLHC1* | | 4 | 6 | 0.856967 |
| *CNKSR2* | | 4 | 6 | 0.856967 |
| *CNTN4* | | 4 | 6 | 0.856967 |
| *CORO6* | | 4 | 6 | 0.856967 |
| *CUL2* | | 4 | 6 | 0.856967 |
| *DCAF12L2* | | 4 | 6 | 0.856967 |
| *DCAF6* | | 4 | 6 | 0.856967 |
| *DCLK1* | | 4 | 6 | 0.856967 |
| *DDX42* | | 4 | 6 | 0.856967 |
| *DNMBP* | | 4 | 6 | 0.856967 |
| *DPP8* | | 4 | 6 | 0.856967 |
| *GALNT15* | | 4 | 6 | 0.856967 |
| *GIGYF1* | | 4 | 6 | 0.856967 |
| *GPNMB* | | 4 | 6 | 0.856967 |
| *GPR116* | | 4 | 6 | 0.856967 |
| *GUCY2F* | | 4 | 6 | 0.856967 |
| *HACL1* | | 4 | 6 | 0.856967 |
| *HAUS3* | | 4 | 6 | 0.856967 |
| *HERC6* | | 4 | 6 | 0.856967 |
| *HNRNPUL1* | | 4 | 6 | 0.856967 |
| *HSPA1L* | | 4 | 6 | 0.856967 |
| *IL1RAPL1* | | 4 | 6 | 0.856967 |
| *INSR* | | 4 | 6 | 0.856967 |
| *ITIH1* | | 4 | 6 | 0.856967 |
| *KIAA1324* | | 4 | 6 | 0.856967 |
| *KIAA1462* | | 4 | 6 | 0.856967 |
| *KIAA1524* | | 4 | 6 | 0.856967 |
| *KIF5A* | | 4 | 6 | 0.856967 |
| *KIT* | | 4 | 6 | 0.856967 |
| *LAMC3* | | 4 | 6 | 0.856967 |
| *LIFR* | | 4 | 6 | 0.856967 |
| *LMTK3* | | 4 | 6 | 0.856967 |
| *MATN4* | | 4 | 6 | 0.856967 |
| *MCM7* | | 4 | 6 | 0.856967 |
| *MTIF2* | | 4 | 6 | 0.856967 |
| *NAP1L1* | | 4 | 6 | 0.856967 |
| *NCAN* | | 4 | 6 | 0.856967 |
| *NCAPD2* | | 4 | 6 | 0.856967 |
| *NFATC3* | | 4 | 6 | 0.856967 |
| *NGLY1* | | 4 | 6 | 0.856967 |
| *NLGN1* | | 4 | 6 | 0.856967 |
| *OR4P4* | | 4 | 6 | 0.856967 |
| *PCDHB7* | | 4 | 6 | 0.856967 |
| *PCDHGA4* | | 4 | 6 | 0.856967 |
| *PCDHGB4* | | 4 | 6 | 0.856967 |
| *PDE3B* | | 4 | 6 | 0.856967 |
| *PDZRN4* | | 4 | 6 | 0.856967 |
| *PKN2* | | 4 | 6 | 0.856967 |
| *PKP2* | | 4 | 6 | 0.856967 |
| *PLK3* | | 4 | 6 | 0.856967 |
| *PLXNC1* | | 4 | 6 | 0.856967 |
| *PNLDC1* | | 4 | 6 | 0.856967 |
| *PPP1R9A* | | 4 | 6 | 0.856967 |
| *PRAM1* | | 4 | 6 | 0.856967 |
| *PRDM4* | | 4 | 6 | 0.856967 |
| *QRICH1* | | 4 | 6 | 0.856967 |
| *RAB3GAP1* | | 4 | 6 | 0.856967 |
| *RASGRF1* | | 4 | 6 | 0.856967 |
| *RBM14* | | 4 | 6 | 0.856967 |
| *RRP12* | | 4 | 6 | 0.856967 |
| *RTEL1* | | 4 | 6 | 0.856967 |
| *SAFB* | | 4 | 6 | 0.856967 |
| *SCAP* | | 4 | 6 | 0.856967 |
| *SIDT1* | | 4 | 6 | 0.856967 |
| *SIRT1* | | 4 | 6 | 0.856967 |
| *SLC29A4* | | 4 | 6 | 0.856967 |
| *SLC4A5* | | 4 | 6 | 0.856967 |
| *SPECC1L* | | 4 | 6 | 0.856967 |
| *SPINK5* | | 4 | 6 | 0.856967 |
| *TAF2* | | 4 | 6 | 0.856967 |
| *TGM4* | | 4 | 6 | 0.856967 |
| *TLR3* | | 4 | 6 | 0.856967 |
| *TMF1* | | 4 | 6 | 0.856967 |
| *TRPM7* | | 4 | 6 | 0.856967 |
| *TTI1* | | 4 | 6 | 0.856967 |
| *TYR* | | 4 | 6 | 0.856967 |
| *UBAP2L* | | 4 | 6 | 0.856967 |
| *UBASH3A* | | 4 | 6 | 0.856967 |
| *USP37* | | 4 | 6 | 0.856967 |
| *ZMIZ1* | | 4 | 6 | 0.856967 |
| *ZNF112* | | 4 | 6 | 0.856967 |
| *ZNF224* | | 4 | 6 | 0.856967 |
| *ZNF263* | | 4 | 6 | 0.856967 |
| *ZNF543* | | 4 | 6 | 0.856967 |
| *ZNF560* | | 4 | 6 | 0.856967 |
| *ZNF721* | | 4 | 6 | 0.856967 |
| *ZSWIM5* | | 4 | 6 | 0.856967 |
| *ALYREF* | | 7 | 4 | 0.856967 |
| *ANLN* | | 7 | 4 | 0.856967 |
| *AZI1* | | 7 | 4 | 0.856967 |
| *BCAS3* | | 7 | 4 | 0.856967 |
| *CDH13* | | 7 | 4 | 0.856967 |
| *CDHR5* | | 7 | 4 | 0.856967 |
| *CSF1R* | | 7 | 4 | 0.856967 |
| *CUX1* | | 7 | 4 | 0.856967 |
| *DCLK2* | | 7 | 4 | 0.856967 |
| *DDX5* | | 7 | 4 | 0.856967 |
| *DHX8* | | 7 | 4 | 0.856967 |
| *DSEL* | | 7 | 4 | 0.856967 |
| *EMILIN3* | | 7 | 4 | 0.856967 |
| *EZR* | | 7 | 4 | 0.856967 |
| *FAM178A* | | 7 | 4 | 0.856967 |
| *GARNL3* | | 7 | 4 | 0.856967 |
| *HECW2* | | 7 | 4 | 0.856967 |
| *IGF1R* | | 7 | 4 | 0.856967 |
| *INSRR* | | 7 | 4 | 0.856967 |
| *KCNC4* | | 7 | 4 | 0.856967 |
| *KCNQ3* | | 7 | 4 | 0.856967 |
| *KCNT2* | | 7 | 4 | 0.856967 |
| *KIAA0430* | | 7 | 4 | 0.856967 |
| *LIMA1* | | 7 | 4 | 0.856967 |
| *LTBP2* | | 7 | 4 | 0.856967 |
| *NASP* | | 7 | 4 | 0.856967 |
| *PCDH11X* | | 7 | 4 | 0.856967 |
| *PLK4* | | 7 | 4 | 0.856967 |
| *PRDM15* | | 7 | 4 | 0.856967 |
| *RBAK* | | 7 | 4 | 0.856967 |
| *RPS6KA6* | | 7 | 4 | 0.856967 |
| *SALL2* | | 7 | 4 | 0.856967 |
| *SERINC3* | | 7 | 4 | 0.856967 |
| *SMG8* | | 7 | 4 | 0.856967 |
| *STAT1* | | 7 | 4 | 0.856967 |
| *SUPT20H* | | 7 | 4 | 0.856967 |
| *TAOK3* | | 7 | 4 | 0.856967 |
| *TARBP1* | | 7 | 4 | 0.856967 |
| *TEC* | | 7 | 4 | 0.856967 |
| *TIE1* | | 7 | 4 | 0.856967 |
| *TOP2A* | | 7 | 4 | 0.856967 |
| *TRAPPC8* | | 7 | 4 | 0.856967 |
| *TSC2* | | 7 | 4 | 0.856967 |
| *UBE3A* | | 7 | 4 | 0.856967 |
| *UBE3C* | | 7 | 4 | 0.856967 |
| *UBN2* | | 7 | 4 | 0.856967 |
| *UVSSA* | | 7 | 4 | 0.856967 |
| *ZBTB40* | | 7 | 4 | 0.856967 |
| *ZNF229* | | 7 | 4 | 0.856967 |
| *ZNF354A* | | 7 | 4 | 0.856967 |
| *ZNF483* | | 7 | 4 | 0.856967 |
| *FAT2* | | 15 | 11 | 0.856967 |
| *LAMA1* | | 15 | 11 | 0.856967 |
| *RANBP2* | | 15 | 11 | 0.856967 |
| *SPAG17* | | 15 | 11 | 0.856967 |
| *ARHGAP35* | | 11 | 14 | 0.856967 |
| *COL14A1* | | 11 | 14 | 0.856967 |
| *EPG5* | | 11 | 14 | 0.856967 |
| *CHD7* | | 16 | 12 | 0.875995 |
| *LAMA2* | | 16 | 12 | 0.875995 |
| *UBR5* | | 16 | 12 | 0.875995 |
| *CHD6* | | 13 | 16 | 0.875995 |
| *ABCB1* | | 5 | 7 | 0.875995 |
| *ADAM30* | | 5 | 7 | 0.875995 |
| *ADCY6* | | 5 | 7 | 0.875995 |
| *ARHGAP21* | | 5 | 7 | 0.875995 |
| *ARID4B* | | 5 | 7 | 0.875995 |
| *C9orf84* | | 5 | 7 | 0.875995 |
| *CACNA1C* | | 5 | 7 | 0.875995 |
| *CBLB* | | 5 | 7 | 0.875995 |
| *CEBPZ* | | 5 | 7 | 0.875995 |
| *CSNK2A1* | | 5 | 7 | 0.875995 |
| *DHTKD1* | | 5 | 7 | 0.875995 |
| *DHX15* | | 5 | 7 | 0.875995 |
| *DHX34* | | 5 | 7 | 0.875995 |
| *DNAJC6* | | 5 | 7 | 0.875995 |
| *ELAVL4* | | 5 | 7 | 0.875995 |
| *ERBB4* | | 5 | 7 | 0.875995 |
| *FAM83G* | | 5 | 7 | 0.875995 |
| *FASN* | | 5 | 7 | 0.875995 |
| *GUCY2C* | | 5 | 7 | 0.875995 |
| *HEXIM1* | | 5 | 7 | 0.875995 |
| *ICAM5* | | 5 | 7 | 0.875995 |
| *KANK1* | | 5 | 7 | 0.875995 |
| *KCNH7* | | 5 | 7 | 0.875995 |
| *KIF1A* | | 5 | 7 | 0.875995 |
| *MTMR4* | | 5 | 7 | 0.875995 |
| *NOTCH4* | | 5 | 7 | 0.875995 |
| *NSMAF* | | 5 | 7 | 0.875995 |
| *NWD1* | | 5 | 7 | 0.875995 |
| *PCDH17* | | 5 | 7 | 0.875995 |
| *PCDHB4* | | 5 | 7 | 0.875995 |
| *PCDHB8* | | 5 | 7 | 0.875995 |
| *PHF20L1* | | 5 | 7 | 0.875995 |
| *PRX* | | 5 | 7 | 0.875995 |
| *RFTN2* | | 5 | 7 | 0.875995 |
| *SESN3* | | 5 | 7 | 0.875995 |
| *SIN3A* | | 5 | 7 | 0.875995 |
| *SLC26A8* | | 5 | 7 | 0.875995 |
| *SRRT* | | 5 | 7 | 0.875995 |
| *TAF3* | | 5 | 7 | 0.875995 |
| *TM9SF4* | | 5 | 7 | 0.875995 |
| *TMEM132B* | | 5 | 7 | 0.875995 |
| *TRIM37* | | 5 | 7 | 0.875995 |
| *TTLL7* | | 5 | 7 | 0.875995 |
| *USP32* | | 5 | 7 | 0.875995 |
| *VPS13A* | | 5 | 7 | 0.875995 |
| *WDR11* | | 5 | 7 | 0.875995 |
| *WDR52* | | 5 | 7 | 0.875995 |
| *ZC3HAV1* | | 5 | 7 | 0.875995 |
| *ZNF600* | | 5 | 7 | 0.875995 |
| *ADAM22* | | 8 | 5 | 0.875995 |
| *AP3B1* | | 8 | 5 | 0.875995 |
| *BRD4* | | 8 | 5 | 0.875995 |
| *CARD11* | | 8 | 5 | 0.875995 |
| *CCDC96* | | 8 | 5 | 0.875995 |
| *CDC42BPA* | | 8 | 5 | 0.875995 |
| *CDH7* | | 8 | 5 | 0.875995 |
| *COG5* | | 8 | 5 | 0.875995 |
| *COL4A3* | | 8 | 5 | 0.875995 |
| *DIP2B* | | 8 | 5 | 0.875995 |
| *ENAM* | | 8 | 5 | 0.875995 |
| *EXOSC10* | | 8 | 5 | 0.875995 |
| *FANCI* | | 8 | 5 | 0.875995 |
| *GRIK5* | | 8 | 5 | 0.875995 |
| *HIPK3* | | 8 | 5 | 0.875995 |
| *KIAA0368* | | 8 | 5 | 0.875995 |
| *LAMC1* | | 8 | 5 | 0.875995 |
| *LCT* | | 8 | 5 | 0.875995 |
| *MBD1* | | 8 | 5 | 0.875995 |
| *MYH8* | | 8 | 5 | 0.875995 |
| *MYO5B* | | 8 | 5 | 0.875995 |
| *NAA25* | | 8 | 5 | 0.875995 |
| *PDE3A* | | 8 | 5 | 0.875995 |
| *PPP2R3A* | | 8 | 5 | 0.875995 |
| *RAD51AP2* | | 8 | 5 | 0.875995 |
| *SLC32A1* | | 8 | 5 | 0.875995 |
| *SLK* | | 8 | 5 | 0.875995 |
| *SP100* | | 8 | 5 | 0.875995 |
| *ST18* | | 8 | 5 | 0.875995 |
| *STXBP5L* | | 8 | 5 | 0.875995 |
| *TOPBP1* | | 8 | 5 | 0.875995 |
| *UGGT2* | | 8 | 5 | 0.875995 |
| *HERC1* | | 18 | 14 | 0.88407 |
| *FRY* | | 14 | 17 | 0.88407 |
| *HERC2* | | 14 | 17 | 0.88407 |
| *PKHD1* | | 14 | 17 | 0.88407 |
| *VPS13B* | | 14 | 17 | 0.88407 |
| *RNF213* | | 19 | 15 | 0.894962 |
| *SRCAP* | | 15 | 18 | 0.894962 |
| *FLG* | | 32 | 36 | 0.894962 |
| *CAMSAP2* | | 9 | 6 | 0.894962 |
| *CD109* | | 9 | 6 | 0.894962 |
| *CELSR2* | | 9 | 6 | 0.894962 |
| *COL24A1* | | 9 | 6 | 0.894962 |
| *EMILIN2* | | 9 | 6 | 0.894962 |
| *EVC2* | | 9 | 6 | 0.894962 |
| *GABRA4* | | 9 | 6 | 0.894962 |
| *INADL* | | 9 | 6 | 0.894962 |
| *KCNB2* | | 9 | 6 | 0.894962 |
| *KIAA1217* | | 9 | 6 | 0.894962 |
| *KIAA1377* | | 9 | 6 | 0.894962 |
| *KIF13A* | | 9 | 6 | 0.894962 |
| *NFAT5* | | 9 | 6 | 0.894962 |
| *PTPN22* | | 9 | 6 | 0.894962 |
| *SUV420H1* | | 9 | 6 | 0.894962 |
| *TEP1* | | 9 | 6 | 0.894962 |
| *TRAPPC10* | | 9 | 6 | 0.894962 |
| *UNC13B* | | 9 | 6 | 0.894962 |
| *UPF2* | | 9 | 6 | 0.894962 |
| *ZNF462* | | 9 | 6 | 0.894962 |
| *ZNF644* | | 9 | 6 | 0.894962 |
| *ACSS3* | | 6 | 8 | 0.894962 |
| *ADAMTS3* | | 6 | 8 | 0.894962 |
| *CILP2* | | 6 | 8 | 0.894962 |
| *DCAF12* | | 6 | 8 | 0.894962 |
| *DICER1* | | 6 | 8 | 0.894962 |
| *EFCAB6* | | 6 | 8 | 0.894962 |
| *FLRT2* | | 6 | 8 | 0.894962 |
| *KDM3A* | | 6 | 8 | 0.894962 |
| *KIAA0754* | | 6 | 8 | 0.894962 |
| *KIAA1033* | | 6 | 8 | 0.894962 |
| *LAMB1* | | 6 | 8 | 0.894962 |
| *LY75* | | 6 | 8 | 0.894962 |
| *MCF2L2* | | 6 | 8 | 0.894962 |
| *NBR1* | | 6 | 8 | 0.894962 |
| *NID2* | | 6 | 8 | 0.894962 |
| *NOS1* | | 6 | 8 | 0.894962 |
| *NPHP3* | | 6 | 8 | 0.894962 |
| *NRG1* | | 6 | 8 | 0.894962 |
| *NYNRIN* | | 6 | 8 | 0.894962 |
| *PHKA1* | | 6 | 8 | 0.894962 |
| *PYGM* | | 6 | 8 | 0.894962 |
| *SCAF11* | | 6 | 8 | 0.894962 |
| *SEMA5B* | | 6 | 8 | 0.894962 |
| *SIPA1L2* | | 6 | 8 | 0.894962 |
| *SMC5* | | 6 | 8 | 0.894962 |
| *SORL1* | | 6 | 8 | 0.894962 |
| *SSH2* | | 6 | 8 | 0.894962 |
| *USP47* | | 6 | 8 | 0.894962 |
| *WDR33* | | 6 | 8 | 0.894962 |
| *LAMA3* | | 16 | 19 | 0.895576 |
| *ADAM29* | | 7 | 9 | 0.913546 |
| *ANKRD30A* | | 10 | 7 | 0.913546 |
| *AXDND1* | | 7 | 9 | 0.913546 |
| *BCLAF1* | | 10 | 7 | 0.913546 |
| *CAMTA1* | | 10 | 7 | 0.913546 |
| *CFTR* | | 10 | 7 | 0.913546 |
| *CHD5* | | 10 | 7 | 0.913546 |
| *CNTLN* | | 7 | 9 | 0.913546 |
| *CNTN6* | | 7 | 9 | 0.913546 |
| *COL19A1* | | 7 | 9 | 0.913546 |
| *CSPG4* | | 7 | 9 | 0.913546 |
| *CUL9* | | 10 | 7 | 0.913546 |
| *DENND4C* | | 7 | 9 | 0.913546 |
| *DOCK4* | | 7 | 9 | 0.913546 |
| *DOT1L* | | 7 | 9 | 0.913546 |
| *FANCM* | | 7 | 9 | 0.913546 |
| *FBF1* | | 7 | 9 | 0.913546 |
| *GPC5* | | 10 | 7 | 0.913546 |
| *HPS3* | | 7 | 9 | 0.913546 |
| *KAT6A* | | 10 | 7 | 0.913546 |
| *KIAA1522* | | 7 | 9 | 0.913546 |
| *KIAA2026* | | 10 | 7 | 0.913546 |
| *KIF26B* | | 7 | 9 | 0.913546 |
| *LRRCC1* | | 7 | 9 | 0.913546 |
| *MAP1A* | | 10 | 7 | 0.913546 |
| *MST1R* | | 10 | 7 | 0.913546 |
| *MYO10* | | 10 | 7 | 0.913546 |
| *MYOF* | | 10 | 7 | 0.913546 |
| *NUP214* | | 10 | 7 | 0.913546 |
| *PHRF1* | | 10 | 7 | 0.913546 |
| *PSME4* | | 7 | 9 | 0.913546 |
| *RP11-407N17.3* | | 7 | 9 | 0.913546 |
| *SETD5* | | 7 | 9 | 0.913546 |
| *SYNPO2* | | 7 | 9 | 0.913546 |
| *TMEM131* | | 7 | 9 | 0.913546 |
| *TNRC6A* | | 7 | 9 | 0.913546 |
| *ZFP36L2* | | 7 | 9 | 0.913546 |
| *GPR98* | | 24 | 20 | 0.929045 |
| *ABCB5* | | 8 | 10 | 0.933755 |
| *ADAMTSL1* | | 8 | 10 | 0.933755 |
| *AUTS2* | | 8 | 10 | 0.933755 |
| *C3orf70* | | 8 | 10 | 0.933755 |
| *ESPL1* | | 8 | 10 | 0.933755 |
| *IPO7* | | 8 | 10 | 0.933755 |
| *MYO9B* | | 8 | 10 | 0.933755 |
| *PKD1L1* | | 8 | 10 | 0.933755 |
| *PPP1R3A* | | 8 | 10 | 0.933755 |
| *RLF* | | 8 | 10 | 0.933755 |
| *SCN3A* | | 8 | 10 | 0.933755 |
| *SDK1* | | 8 | 10 | 0.933755 |
| *SLIT2* | | 8 | 10 | 0.933755 |
| *TXNIP* | | 8 | 10 | 0.933755 |
| *ZEB2* | | 8 | 10 | 0.933755 |
| *MYH3* | | 11 | 8 | 0.933755 |
| *ROS1* | | 11 | 8 | 0.933755 |
| *XIRP2* | | 23 | 26 | 0.948047 |
| *ASCC3* | | 9 | 11 | 0.948047 |
| *HIVEP3* | | 9 | 11 | 0.948047 |
| *KIDINS220* | | 9 | 11 | 0.948047 |
| *LPHN2* | | 9 | 11 | 0.948047 |
| *PTPRT* | | 9 | 11 | 0.948047 |
| *SPTBN4* | | 9 | 11 | 0.948047 |
| *ZNF236* | | 9 | 11 | 0.948047 |
| *BRWD1* | | 12 | 9 | 0.948047 |
| *DHX9* | | 12 | 9 | 0.948047 |
| *MYH15* | | 12 | 9 | 0.948047 |
| *MYT1L* | | 12 | 9 | 0.948047 |
| *TEX15* | | 12 | 9 | 0.948047 |
| *ZNF91* | | 12 | 9 | 0.948047 |
| *BIRC6* | | 24 | 27 | 0.948047 |
| *DNAH5* | | 25 | 28 | 0.948047 |
| *CNOT1* | | 10 | 12 | 0.948047 |
| *KIAA1429* | | 13 | 10 | 0.948047 |
| *KMT2B* | | 13 | 10 | 0.948047 |
| *PBRM1* | | 13 | 10 | 0.948047 |
| *ADAMTS12* | | 11 | 13 | 0.948047 |
| *DNAH1* | | 11 | 13 | 0.948047 |
| *REV3L* | | 11 | 13 | 0.948047 |
| *ABCA12* | | 14 | 11 | 0.948047 |
| *HYDIN* | | 14 | 11 | 0.948047 |
| *SVEP1* | | 14 | 11 | 0.948047 |
| *COL11A1* | | 12 | 14 | 0.948047 |
| *NUP205* | | 12 | 14 | 0.948047 |
| *RHOB* | | 12 | 14 | 0.948047 |
| *FBN1* | | 13 | 15 | 0.948047 |
| *VPS13C* | | 13 | 15 | 0.948047 |
| *NF1* | | 14 | 16 | 0.948047 |
| *PCNT* | | 14 | 16 | 0.948047 |
| *BDP1* | | 15 | 17 | 0.948047 |
| *DNAH9* | | 15 | 17 | 0.948047 |
| *ABCC10* | | 5 | 3 | 0.948047 |
| *ACTL6B* | | 5 | 3 | 0.948047 |
| *ACTR8* | | 5 | 3 | 0.948047 |
| *ACY3* | | 5 | 3 | 0.948047 |
| *ADCY4* | | 5 | 3 | 0.948047 |
| *ADCY9* | | 5 | 3 | 0.948047 |
| *ADD3* | | 5 | 3 | 0.948047 |
| *AFAP1* | | 5 | 3 | 0.948047 |
| *ANAPC2* | | 5 | 3 | 0.948047 |
| *ANO9* | | 5 | 3 | 0.948047 |
| *BBS10* | | 5 | 3 | 0.948047 |
| *BTBD10* | | 5 | 3 | 0.948047 |
| *C5* | | 5 | 3 | 0.948047 |
| *CALCRL* | | 5 | 3 | 0.948047 |
| *CAMSAP3* | | 5 | 3 | 0.948047 |
| *CCDC171* | | 5 | 3 | 0.948047 |
| *CEP135* | | 5 | 3 | 0.948047 |
| *CHERP* | | 5 | 3 | 0.948047 |
| *CLCN1* | | 5 | 3 | 0.948047 |
| *COG8* | | 5 | 3 | 0.948047 |
| *COL1A2* | | 5 | 3 | 0.948047 |
| *COPS2* | | 5 | 3 | 0.948047 |
| *COPS4* | | 5 | 3 | 0.948047 |
| *CORIN* | | 5 | 3 | 0.948047 |
| *CYC1* | | 5 | 3 | 0.948047 |
| *DAXX* | | 5 | 3 | 0.948047 |
| *DDHD2* | | 5 | 3 | 0.948047 |
| *DNMT3B* | | 5 | 3 | 0.948047 |
| *DNTT* | | 5 | 3 | 0.948047 |
| *ENTHD1* | | 5 | 3 | 0.948047 |
| *EPB41L1* | | 5 | 3 | 0.948047 |
| *F13A1* | | 5 | 3 | 0.948047 |
| *FAM160A2* | | 5 | 3 | 0.948047 |
| *FAM198B* | | 5 | 3 | 0.948047 |
| *FHOD1* | | 5 | 3 | 0.948047 |
| *FLT1* | | 5 | 3 | 0.948047 |
| *GBP1* | | 5 | 3 | 0.948047 |
| *GDF11* | | 5 | 3 | 0.948047 |
| *GINM1* | | 5 | 3 | 0.948047 |
| *GLTSCR1L* | | 5 | 3 | 0.948047 |
| *GPRC6A* | | 5 | 3 | 0.948047 |
| *HAO2* | | 5 | 3 | 0.948047 |
| *HIST1H1B* | | 5 | 3 | 0.948047 |
| *HIST1H1C* | | 5 | 3 | 0.948047 |
| *HIST1H2AG* | | 5 | 3 | 0.948047 |
| *HMGCS1* | | 5 | 3 | 0.948047 |
| *HMHA1* | | 5 | 3 | 0.948047 |
| *IFI16* | | 5 | 3 | 0.948047 |
| *IL10RA* | | 5 | 3 | 0.948047 |
| *INO80D* | | 5 | 3 | 0.948047 |
| *INTS7* | | 5 | 3 | 0.948047 |
| *IPO13* | | 5 | 3 | 0.948047 |
| *ITGA10* | | 5 | 3 | 0.948047 |
| *KAL1* | | 5 | 3 | 0.948047 |
| *KDM4C* | | 5 | 3 | 0.948047 |
| *KIAA1407* | | 5 | 3 | 0.948047 |
| *KIFC1* | | 5 | 3 | 0.948047 |
| *KL* | | 5 | 3 | 0.948047 |
| *KRI1* | | 5 | 3 | 0.948047 |
| *LMNB2* | | 5 | 3 | 0.948047 |
| *MAN2B1* | | 5 | 3 | 0.948047 |
| *MAN2C1* | | 5 | 3 | 0.948047 |
| *MAP7* | | 5 | 3 | 0.948047 |
| *MCF2L* | | 5 | 3 | 0.948047 |
| *MCTP2* | | 5 | 3 | 0.948047 |
| *METTL25* | | 5 | 3 | 0.948047 |
| *MLTK* | | 5 | 3 | 0.948047 |
| *MTERFD2* | | 5 | 3 | 0.948047 |
| *MTMR6* | | 5 | 3 | 0.948047 |
| *MUSK* | | 5 | 3 | 0.948047 |
| *MYNN* | | 5 | 3 | 0.948047 |
| *MYOCD* | | 5 | 3 | 0.948047 |
| *NRIP1* | | 5 | 3 | 0.948047 |
| *OR10R2* | | 5 | 3 | 0.948047 |
| *OR4A15* | | 5 | 3 | 0.948047 |
| *OR8B4* | | 5 | 3 | 0.948047 |
| *OTUD7B* | | 5 | 3 | 0.948047 |
| *PADI2* | | 5 | 3 | 0.948047 |
| *PARPBP* | | 5 | 3 | 0.948047 |
| *PCDHAC1* | | 5 | 3 | 0.948047 |
| *PDE4C* | | 5 | 3 | 0.948047 |
| *PHLDA3* | | 5 | 3 | 0.948047 |
| *PHLDB1* | | 5 | 3 | 0.948047 |
| *PHLPP1* | | 5 | 3 | 0.948047 |
| *PLXNB3* | | 5 | 3 | 0.948047 |
| *PPARGC1B* | | 5 | 3 | 0.948047 |
| *PRKG1* | | 5 | 3 | 0.948047 |
| *PRKG2* | | 5 | 3 | 0.948047 |
| *PSEN1* | | 5 | 3 | 0.948047 |
| *PSG1* | | 5 | 3 | 0.948047 |
| *PTGFRN* | | 5 | 3 | 0.948047 |
| *RAD51* | | 5 | 3 | 0.948047 |
| *RAD54L2* | | 5 | 3 | 0.948047 |
| *RAP1GDS1* | | 5 | 3 | 0.948047 |
| *RARS2* | | 5 | 3 | 0.948047 |
| *RECK* | | 5 | 3 | 0.948047 |
| *RECQL5* | | 5 | 3 | 0.948047 |
| *RFC1* | | 5 | 3 | 0.948047 |
| *RFX3* | | 5 | 3 | 0.948047 |
| *RPH3A* | | 5 | 3 | 0.948047 |
| *SCNM1* | | 5 | 3 | 0.948047 |
| *SEL1L* | | 5 | 3 | 0.948047 |
| *SERPINC1* | | 5 | 3 | 0.948047 |
| *SH3PXD2A* | | 5 | 3 | 0.948047 |
| *SIRPB1* | | 5 | 3 | 0.948047 |
| *SLC12A4* | | 5 | 3 | 0.948047 |
| *SLC9A3* | | 5 | 3 | 0.948047 |
| *SLITRK6* | | 5 | 3 | 0.948047 |
| *SMAD4* | | 5 | 3 | 0.948047 |
| *SNAPC4* | | 5 | 3 | 0.948047 |
| *STXBP1* | | 5 | 3 | 0.948047 |
| *SULF2* | | 5 | 3 | 0.948047 |
| *TAB3* | | 5 | 3 | 0.948047 |
| *TELO2* | | 5 | 3 | 0.948047 |
| *TGIF2LX* | | 5 | 3 | 0.948047 |
| *TMC2* | | 5 | 3 | 0.948047 |
| *TMCO3* | | 5 | 3 | 0.948047 |
| *TOPORS* | | 5 | 3 | 0.948047 |
| *TTK* | | 5 | 3 | 0.948047 |
| *UBE4B* | | 5 | 3 | 0.948047 |
| *WASF2* | | 5 | 3 | 0.948047 |
| *ZCWPW1* | | 5 | 3 | 0.948047 |
| *ZMYM2* | | 5 | 3 | 0.948047 |
| *ZNF148* | | 5 | 3 | 0.948047 |
| *ZNF184* | | 5 | 3 | 0.948047 |
| *ZNF45* | | 5 | 3 | 0.948047 |
| *ZNF836* | | 5 | 3 | 0.948047 |
| *ANK3* | | 16 | 18 | 0.948047 |
| *ASXL2* | | 17 | 19 | 0.948047 |
| *USP34* | | 17 | 19 | 0.948047 |
| *DNAH3* | | 19 | 21 | 0.948047 |
| *ABCA7* | | 6 | 4 | 0.948047 |
| *ACPP* | | 6 | 4 | 0.948047 |
| *AK9* | | 6 | 4 | 0.948047 |
| *ARHGEF18* | | 6 | 4 | 0.948047 |
| *ATP11A* | | 6 | 4 | 0.948047 |
| *B4GALNT3* | | 6 | 4 | 0.948047 |
| *BRINP2* | | 6 | 4 | 0.948047 |
| *CD2AP* | | 6 | 4 | 0.948047 |
| *CD93* | | 6 | 4 | 0.948047 |
| *CREB5* | | 6 | 4 | 0.948047 |
| *CRTC2* | | 6 | 4 | 0.948047 |
| *CTNNA3* | | 6 | 4 | 0.948047 |
| *CWC27* | | 6 | 4 | 0.948047 |
| *CXXC1* | | 6 | 4 | 0.948047 |
| *DAAM1* | | 6 | 4 | 0.948047 |
| *DCT* | | 6 | 4 | 0.948047 |
| *DDX20* | | 6 | 4 | 0.948047 |
| *DIEXF* | | 6 | 4 | 0.948047 |
| *DLG2* | | 6 | 4 | 0.948047 |
| *DSG4* | | 6 | 4 | 0.948047 |
| *EDC4* | | 6 | 4 | 0.948047 |
| *EHBP1L1* | | 6 | 4 | 0.948047 |
| *ELTD1* | | 6 | 4 | 0.948047 |
| *F11* | | 6 | 4 | 0.948047 |
| *FAM47C* | | 6 | 4 | 0.948047 |
| *FLT3* | | 6 | 4 | 0.948047 |
| *FNDC3B* | | 6 | 4 | 0.948047 |
| *GLUD2* | | 6 | 4 | 0.948047 |
| *GRIK1* | | 6 | 4 | 0.948047 |
| *GRM2* | | 6 | 4 | 0.948047 |
| *HKDC1* | | 6 | 4 | 0.948047 |
| *ITCH* | | 6 | 4 | 0.948047 |
| *KCNH5* | | 6 | 4 | 0.948047 |
| *KIAA1045* | | 6 | 4 | 0.948047 |
| *KIAA1614* | | 6 | 4 | 0.948047 |
| *KIF26A* | | 6 | 4 | 0.948047 |
| *KPRP* | | 6 | 4 | 0.948047 |
| *LPIN2* | | 6 | 4 | 0.948047 |
| *LRIG1* | | 6 | 4 | 0.948047 |
| *LRIG3* | | 6 | 4 | 0.948047 |
| *MAP3K1* | | 6 | 4 | 0.948047 |
| *MMP16* | | 6 | 4 | 0.948047 |
| *NKX2-2* | | 6 | 4 | 0.948047 |
| *NOL8* | | 6 | 4 | 0.948047 |
| *NOS2* | | 6 | 4 | 0.948047 |
| *OPCML* | | 6 | 4 | 0.948047 |
| *PAH* | | 6 | 4 | 0.948047 |
| *PARD3B* | | 6 | 4 | 0.948047 |
| *PCDH19* | | 6 | 4 | 0.948047 |
| *PCDH8* | | 6 | 4 | 0.948047 |
| *PCDHA13* | | 6 | 4 | 0.948047 |
| *PDE12* | | 6 | 4 | 0.948047 |
| *PDE4A* | | 6 | 4 | 0.948047 |
| *PDZRN3* | | 6 | 4 | 0.948047 |
| *PRPF3* | | 6 | 4 | 0.948047 |
| *PRSS12* | | 6 | 4 | 0.948047 |
| *SAP130* | | 6 | 4 | 0.948047 |
| *SCNN1A* | | 6 | 4 | 0.948047 |
| *SEMA3A* | | 6 | 4 | 0.948047 |
| *SLC24A1* | | 6 | 4 | 0.948047 |
| *SLC4A10* | | 6 | 4 | 0.948047 |
| *SLCO1B1* | | 6 | 4 | 0.948047 |
| *SLFN11* | | 6 | 4 | 0.948047 |
| *SOGA3* | | 6 | 4 | 0.948047 |
| *STK31* | | 6 | 4 | 0.948047 |
| *SYNE3* | | 6 | 4 | 0.948047 |
| *SYT10* | | 6 | 4 | 0.948047 |
| *TECPR2* | | 6 | 4 | 0.948047 |
| *TET3* | | 6 | 4 | 0.948047 |
| *UNC13A* | | 6 | 4 | 0.948047 |
| *YLPM1* | | 6 | 4 | 0.948047 |
| *ZNF276* | | 6 | 4 | 0.948047 |
| *ZNF35* | | 6 | 4 | 0.948047 |
| *ZNF470* | | 6 | 4 | 0.948047 |
| *ZNF646* | | 6 | 4 | 0.948047 |
| *ZNF687* | | 6 | 4 | 0.948047 |
| *ABCB4* | | 4 | 5 | 0.948047 |
| *AKR1E2* | | 4 | 5 | 0.948047 |
| *AMER2* | | 4 | 5 | 0.948047 |
| *AMOTL2* | | 4 | 5 | 0.948047 |
| *ANO1* | | 4 | 5 | 0.948047 |
| *ARHGEF11* | | 4 | 5 | 0.948047 |
| *BMPR2* | | 4 | 5 | 0.948047 |
| *C14orf39* | | 4 | 5 | 0.948047 |
| *CASK* | | 4 | 5 | 0.948047 |
| *CASZ1* | | 4 | 5 | 0.948047 |
| *CC2D1A* | | 4 | 5 | 0.948047 |
| *CCDC39* | | 4 | 5 | 0.948047 |
| *CCDC81* | | 4 | 5 | 0.948047 |
| *CENPB* | | 4 | 5 | 0.948047 |
| *CLIP2* | | 4 | 5 | 0.948047 |
| *CLOCK* | | 4 | 5 | 0.948047 |
| *CMSS1* | | 4 | 5 | 0.948047 |
| *COL1A1* | | 4 | 5 | 0.948047 |
| *COL21A1* | | 4 | 5 | 0.948047 |
| *CRAT* | | 4 | 5 | 0.948047 |
| *CUL3* | | 4 | 5 | 0.948047 |
| *CYP7A1* | | 4 | 5 | 0.948047 |
| *DDR2* | | 4 | 5 | 0.948047 |
| *DDX46* | | 4 | 5 | 0.948047 |
| *DGCR8* | | 4 | 5 | 0.948047 |
| *DGKD* | | 4 | 5 | 0.948047 |
| *DISP2* | | 4 | 5 | 0.948047 |
| *DLGAP3* | | 4 | 5 | 0.948047 |
| *DNA2* | | 4 | 5 | 0.948047 |
| *EIF2AK2* | | 4 | 5 | 0.948047 |
| *ESRP1* | | 4 | 5 | 0.948047 |
| *FAM13A* | | 4 | 5 | 0.948047 |
| *FAM160B1* | | 4 | 5 | 0.948047 |
| *FNBP1* | | 4 | 5 | 0.948047 |
| *FOXP2* | | 4 | 5 | 0.948047 |
| *FRS2* | | 4 | 5 | 0.948047 |
| *GABRB3* | | 4 | 5 | 0.948047 |
| *GEMIN5* | | 4 | 5 | 0.948047 |
| *GLI1* | | 4 | 5 | 0.948047 |
| *GNL3L* | | 4 | 5 | 0.948047 |
| *GPR125* | | 4 | 5 | 0.948047 |
| *GPR75* | | 4 | 5 | 0.948047 |
| *GRAMD1C* | | 4 | 5 | 0.948047 |
| *GRIA2* | | 4 | 5 | 0.948047 |
| *GRIN2D* | | 4 | 5 | 0.948047 |
| *HEATR5A* | | 4 | 5 | 0.948047 |
| *HEATR6* | | 4 | 5 | 0.948047 |
| *HERC4* | | 4 | 5 | 0.948047 |
| *HNRNPK* | | 4 | 5 | 0.948047 |
| *IFT122* | | 4 | 5 | 0.948047 |
| *IL18RAP* | | 4 | 5 | 0.948047 |
| *INPP4B* | | 4 | 5 | 0.948047 |
| *INPPL1* | | 4 | 5 | 0.948047 |
| *IPO9* | | 4 | 5 | 0.948047 |
| *ITGA9* | | 4 | 5 | 0.948047 |
| *KIAA0195* | | 4 | 5 | 0.948047 |
| *KIAA1009* | | 4 | 5 | 0.948047 |
| *KRT33B* | | 4 | 5 | 0.948047 |
| *LARP4B* | | 4 | 5 | 0.948047 |
| *LIG4* | | 4 | 5 | 0.948047 |
| *LOXL3* | | 4 | 5 | 0.948047 |
| *LPHN1* | | 4 | 5 | 0.948047 |
| *MAP3K12* | | 4 | 5 | 0.948047 |
| *MKRN3* | | 4 | 5 | 0.948047 |
| *MOCS3* | | 4 | 5 | 0.948047 |
| *MYT1* | | 4 | 5 | 0.948047 |
| *NBPF3* | | 4 | 5 | 0.948047 |
| *NEBL* | | 4 | 5 | 0.948047 |
| *NELL1* | | 4 | 5 | 0.948047 |
| *NOP9* | | 4 | 5 | 0.948047 |
| *NOX5* | | 4 | 5 | 0.948047 |
| *NPHS1* | | 4 | 5 | 0.948047 |
| *OSMR* | | 4 | 5 | 0.948047 |
| *PAK2* | | 4 | 5 | 0.948047 |
| *PALLD* | | 4 | 5 | 0.948047 |
| *PCDHGA10* | | 4 | 5 | 0.948047 |
| *PCDHGA12* | | 4 | 5 | 0.948047 |
| *PDIA5* | | 4 | 5 | 0.948047 |
| *PER3* | | 4 | 5 | 0.948047 |
| *PIK3C3* | | 4 | 5 | 0.948047 |
| *PLD1* | | 4 | 5 | 0.948047 |
| *PNMAL2* | | 4 | 5 | 0.948047 |
| *POLH* | | 4 | 5 | 0.948047 |
| *PPP1R36* | | 4 | 5 | 0.948047 |
| *PRAMEF12* | | 4 | 5 | 0.948047 |
| *PURB* | | 4 | 5 | 0.948047 |
| *RABEP2* | | 4 | 5 | 0.948047 |
| *RASGRF2* | | 4 | 5 | 0.948047 |
| *RIMBP2* | | 4 | 5 | 0.948047 |
| *RNF40* | | 4 | 5 | 0.948047 |
| *RPS6KA2* | | 4 | 5 | 0.948047 |
| *SATB2* | | 4 | 5 | 0.948047 |
| *SCUBE2* | | 4 | 5 | 0.948047 |
| *SEZ6L2* | | 4 | 5 | 0.948047 |
| *SGOL1* | | 4 | 5 | 0.948047 |
| *SH3RF1* | | 4 | 5 | 0.948047 |
| *SH3TC1* | | 4 | 5 | 0.948047 |
| *SLC25A12* | | 4 | 5 | 0.948047 |
| *SLC29A2* | | 4 | 5 | 0.948047 |
| *SLIT3* | | 4 | 5 | 0.948047 |
| *SND1* | | 4 | 5 | 0.948047 |
| *SNX14* | | 4 | 5 | 0.948047 |
| *SORBS1* | | 4 | 5 | 0.948047 |
| *SREBF2* | | 4 | 5 | 0.948047 |
| *STAT4* | | 4 | 5 | 0.948047 |
| *SYTL3* | | 4 | 5 | 0.948047 |
| *TBX3* | | 4 | 5 | 0.948047 |
| *TDRD3* | | 4 | 5 | 0.948047 |
| *THBS1* | | 4 | 5 | 0.948047 |
| *TJP1* | | 4 | 5 | 0.948047 |
| *TMPRSS11F* | | 4 | 5 | 0.948047 |
| *TNFRSF8* | | 4 | 5 | 0.948047 |
| *TRAK1* | | 4 | 5 | 0.948047 |
| *TRIP11* | | 4 | 5 | 0.948047 |
| *TRPC6* | | 4 | 5 | 0.948047 |
| *TTBK2* | | 4 | 5 | 0.948047 |
| *TTC16* | | 4 | 5 | 0.948047 |
| *TTC21B* | | 4 | 5 | 0.948047 |
| *TULP3* | | 4 | 5 | 0.948047 |
| *UBAP2* | | 4 | 5 | 0.948047 |
| *UBQLN2* | | 4 | 5 | 0.948047 |
| *USP15* | | 4 | 5 | 0.948047 |
| *USP40* | | 4 | 5 | 0.948047 |
| *VWCE* | | 4 | 5 | 0.948047 |
| *YTHDF2* | | 4 | 5 | 0.948047 |
| *ZFR2* | | 4 | 5 | 0.948047 |
| *ZMYM3* | | 4 | 5 | 0.948047 |
| *ZNF142* | | 4 | 5 | 0.948047 |
| *ZNF254* | | 4 | 5 | 0.948047 |
| *ZNF37A* | | 4 | 5 | 0.948047 |
| *ZNF574* | | 4 | 5 | 0.948047 |
| *ZNF578* | | 4 | 5 | 0.948047 |
| *ZNF599* | | 4 | 5 | 0.948047 |
| *ZNF606* | | 4 | 5 | 0.948047 |
| *ZNF667* | | 4 | 5 | 0.948047 |
| *ZNF695* | | 4 | 5 | 0.948047 |
| *ZNF728* | | 4 | 5 | 0.948047 |
| *ZNF839* | | 4 | 5 | 0.948047 |
| *ERBB2* | | 26 | 23 | 0.948047 |
| *OBSCN* | | 28 | 25 | 0.948047 |
| *ABCA1* | | 7 | 5 | 0.948047 |
| *ABCC2* | | 5 | 6 | 0.948047 |
| *ACTN4* | | 7 | 5 | 0.948047 |
| *AMBRA1* | | 5 | 6 | 0.948047 |
| *ANKRD35* | | 5 | 6 | 0.948047 |
| *ANKRD52* | | 5 | 6 | 0.948047 |
| *ANO8* | | 7 | 5 | 0.948047 |
| *AP3B2* | | 5 | 6 | 0.948047 |
| *ARMC3* | | 5 | 6 | 0.948047 |
| *ASAP2* | | 5 | 6 | 0.948047 |
| *ATP6V1A* | | 5 | 6 | 0.948047 |
| *ATP8B2* | | 5 | 6 | 0.948047 |
| *BACH2* | | 5 | 6 | 0.948047 |
| *CADM2* | | 5 | 6 | 0.948047 |
| *CASP1* | | 5 | 6 | 0.948047 |
| *CCDC141* | | 5 | 6 | 0.948047 |
| *CIC* | | 7 | 5 | 0.948047 |
| *CLCA4* | | 5 | 6 | 0.948047 |
| *CNTN1* | | 5 | 6 | 0.948047 |
| *CNTNAP1* | | 5 | 6 | 0.948047 |
| *COL5A1* | | 5 | 6 | 0.948047 |
| *CPS1* | | 7 | 5 | 0.948047 |
| *DACH1* | | 5 | 6 | 0.948047 |
| *DCLRE1A* | | 5 | 6 | 0.948047 |
| *DDHD1* | | 5 | 6 | 0.948047 |
| *DDX18* | | 5 | 6 | 0.948047 |
| *DDX60* | | 5 | 6 | 0.948047 |
| *DLG1* | | 7 | 5 | 0.948047 |
| *DOCK2* | | 7 | 5 | 0.948047 |
| *ECE2* | | 5 | 6 | 0.948047 |
| *EIF4A2* | | 7 | 5 | 0.948047 |
| *EPHB6* | | 5 | 6 | 0.948047 |
| *EPS15* | | 5 | 6 | 0.948047 |
| *ESYT1* | | 7 | 5 | 0.948047 |
| *FAM120A* | | 5 | 6 | 0.948047 |
| *FAM184A* | | 5 | 6 | 0.948047 |
| *FOXM1* | | 5 | 6 | 0.948047 |
| *FRMD4A* | | 5 | 6 | 0.948047 |
| *FZD6* | | 5 | 6 | 0.948047 |
| *GANAB* | | 5 | 6 | 0.948047 |
| *GPR50* | | 7 | 5 | 0.948047 |
| *GRID2* | | 7 | 5 | 0.948047 |
| *GRM7* | | 5 | 6 | 0.948047 |
| *GUCY1A2* | | 7 | 5 | 0.948047 |
| *HIP1* | | 7 | 5 | 0.948047 |
| *IKBKAP* | | 5 | 6 | 0.948047 |
| *INPP5F* | | 5 | 6 | 0.948047 |
| *IRX1* | | 5 | 6 | 0.948047 |
| *ITGA11* | | 5 | 6 | 0.948047 |
| *KANK4* | | 5 | 6 | 0.948047 |
| *KCNA5* | | 5 | 6 | 0.948047 |
| *KIAA1210* | | 7 | 5 | 0.948047 |
| *KIF20B* | | 7 | 5 | 0.948047 |
| *KIF27* | | 5 | 6 | 0.948047 |
| *LRRC36* | | 5 | 6 | 0.948047 |
| *MAP3K9* | | 5 | 6 | 0.948047 |
| *MAP4* | | 5 | 6 | 0.948047 |
| *MAPK8IP3* | | 5 | 6 | 0.948047 |
| *MARK2* | | 7 | 5 | 0.948047 |
| *MSH2* | | 7 | 5 | 0.948047 |
| *MSH6* | | 5 | 6 | 0.948047 |
| *MTHFD1L* | | 5 | 6 | 0.948047 |
| *MTMR3* | | 7 | 5 | 0.948047 |
| *MTOR* | | 5 | 6 | 0.948047 |
| *MYO1B* | | 5 | 6 | 0.948047 |
| *MYO7A* | | 5 | 6 | 0.948047 |
| *NBN* | | 5 | 6 | 0.948047 |
| *NCOA2* | | 7 | 5 | 0.948047 |
| *NEK10* | | 5 | 6 | 0.948047 |
| *NFATC2* | | 7 | 5 | 0.948047 |
| *NOP14* | | 5 | 6 | 0.948047 |
| *NOTCH2* | | 7 | 5 | 0.948047 |
| *NPC1* | | 7 | 5 | 0.948047 |
| *NRXN2* | | 5 | 6 | 0.948047 |
| *NUP107* | | 5 | 6 | 0.948047 |
| *NUTM1* | | 7 | 5 | 0.948047 |
| *OR4N2* | | 5 | 6 | 0.948047 |
| *PCDHB10* | | 5 | 6 | 0.948047 |
| *PLVAP* | | 7 | 5 | 0.948047 |
| *POTEC* | | 5 | 6 | 0.948047 |
| *PSD3* | | 7 | 5 | 0.948047 |
| *PTPN3* | | 5 | 6 | 0.948047 |
| *R3HDM1* | | 5 | 6 | 0.948047 |
| *RBM27* | | 5 | 6 | 0.948047 |
| *RBM6* | | 5 | 6 | 0.948047 |
| *RPAP1* | | 7 | 5 | 0.948047 |
| *RTN3* | | 5 | 6 | 0.948047 |
| *RUSC1* | | 5 | 6 | 0.948047 |
| *SETDB1* | | 5 | 6 | 0.948047 |
| *SEZ6L* | | 5 | 6 | 0.948047 |
| *SF1* | | 5 | 6 | 0.948047 |
| *SHPRH* | | 7 | 5 | 0.948047 |
| *SIPA1L3* | | 7 | 5 | 0.948047 |
| *SKIL* | | 7 | 5 | 0.948047 |
| *SLFN5* | | 5 | 6 | 0.948047 |
| *SMARCAD1* | | 5 | 6 | 0.948047 |
| *SMG7* | | 5 | 6 | 0.948047 |
| *STK32B* | | 5 | 6 | 0.948047 |
| *TAOK2* | | 7 | 5 | 0.948047 |
| *TBC1D4* | | 5 | 6 | 0.948047 |
| *TECRL* | | 5 | 6 | 0.948047 |
| *TLK2* | | 7 | 5 | 0.948047 |
| *TLN2* | | 7 | 5 | 0.948047 |
| *TNKS1BP1* | | 7 | 5 | 0.948047 |
| *TNKS2* | | 5 | 6 | 0.948047 |
| *TNR* | | 7 | 5 | 0.948047 |
| *TRIP12* | | 5 | 6 | 0.948047 |
| *TTC37* | | 5 | 6 | 0.948047 |
| *TTC7B* | | 7 | 5 | 0.948047 |
| *UBA6* | | 5 | 6 | 0.948047 |
| *UBE3B* | | 7 | 5 | 0.948047 |
| *UBR1* | | 5 | 6 | 0.948047 |
| *VWA7* | | 7 | 5 | 0.948047 |
| *WDR36* | | 7 | 5 | 0.948047 |
| *WDR81* | | 5 | 6 | 0.948047 |
| *YEATS2* | | 5 | 6 | 0.948047 |
| *ZBTB7B* | | 5 | 6 | 0.948047 |
| *ZFYVE16* | | 5 | 6 | 0.948047 |
| *ZHX2* | | 5 | 6 | 0.948047 |
| *ZNF385D* | | 7 | 5 | 0.948047 |
| *ZNF518B* | | 5 | 6 | 0.948047 |
| *ZNF594* | | 7 | 5 | 0.948047 |
| *ZNF750* | | 7 | 5 | 0.948047 |
| *ZNF75D* | | 5 | 6 | 0.948047 |
| *ZNF808* | | 5 | 6 | 0.948047 |
| *ZZZ3* | | 5 | 6 | 0.948047 |
| *FAT4* | | 30 | 32 | 0.948626 |
| *ANKRD17* | | 6 | 7 | 0.948626 |
| *AP2B1* | | 6 | 7 | 0.948626 |
| *ARHGAP30* | | 6 | 7 | 0.948626 |
| *ASAP1* | | 6 | 7 | 0.948626 |
| *BRINP3* | | 6 | 7 | 0.948626 |
| *CACNA1H* | | 6 | 7 | 0.948626 |
| *CAP1* | | 6 | 7 | 0.948626 |
| *CEP170* | | 6 | 7 | 0.948626 |
| *CR1* | | 6 | 7 | 0.948626 |
| *CTCF* | | 6 | 7 | 0.948626 |
| *DPYS* | | 6 | 7 | 0.948626 |
| *EIF2AK4* | | 6 | 7 | 0.948626 |
| *EIF3A* | | 6 | 7 | 0.948626 |
| *FNDC3A* | | 6 | 7 | 0.948626 |
| *GTF2I* | | 6 | 7 | 0.948626 |
| *IKZF2* | | 6 | 7 | 0.948626 |
| *ITGAM* | | 6 | 7 | 0.948626 |
| *KNDC1* | | 6 | 7 | 0.948626 |
| *LAMB2* | | 6 | 7 | 0.948626 |
| *MAGI3* | | 6 | 7 | 0.948626 |
| *N4BP2* | | 6 | 7 | 0.948626 |
| *NCOA6* | | 6 | 7 | 0.948626 |
| *NFE2L3* | | 6 | 7 | 0.948626 |
| *NHS* | | 6 | 7 | 0.948626 |
| *OCA2* | | 6 | 7 | 0.948626 |
| *PCDHGA7* | | 6 | 7 | 0.948626 |
| *PCNX* | | 6 | 7 | 0.948626 |
| *PIWIL1* | | 6 | 7 | 0.948626 |
| *PLCG1* | | 6 | 7 | 0.948626 |
| *PLEKHA5* | | 6 | 7 | 0.948626 |
| *PLEKHG6* | | 6 | 7 | 0.948626 |
| *RBP3* | | 6 | 7 | 0.948626 |
| *SLC39A12* | | 6 | 7 | 0.948626 |
| *SLITRK5* | | 6 | 7 | 0.948626 |
| *UGT1A1* | | 6 | 7 | 0.948626 |
| *UHRF1BP1L* | | 6 | 7 | 0.948626 |
| *WHSC1L1* | | 6 | 7 | 0.948626 |
| *WNK3* | | 6 | 7 | 0.948626 |
| *XRN1* | | 6 | 7 | 0.948626 |
| *YTHDC2* | | 6 | 7 | 0.948626 |
| *ALPK3* | | 8 | 6 | 0.948626 |
| *C15orf39* | | 8 | 6 | 0.948626 |
| *CTNNB1* | | 8 | 6 | 0.948626 |
| *DOCK6* | | 8 | 6 | 0.948626 |
| *EPS8* | | 8 | 6 | 0.948626 |
| *ERC2* | | 8 | 6 | 0.948626 |
| *KIAA0556* | | 8 | 6 | 0.948626 |
| *KIF16B* | | 8 | 6 | 0.948626 |
| *KLHL1* | | 8 | 6 | 0.948626 |
| *LPA* | | 8 | 6 | 0.948626 |
| *LRFN5* | | 8 | 6 | 0.948626 |
| *MBTPS1* | | 8 | 6 | 0.948626 |
| *PLCL2* | | 8 | 6 | 0.948626 |
| *PTPRS* | | 8 | 6 | 0.948626 |
| *RBM15* | | 8 | 6 | 0.948626 |
| *RERE* | | 8 | 6 | 0.948626 |
| *SHANK2* | | 8 | 6 | 0.948626 |
| *SPATA31D1* | | 8 | 6 | 0.948626 |
| *TEX2* | | 8 | 6 | 0.948626 |
| *TMEM132E* | | 8 | 6 | 0.948626 |
| *TRPM1* | | 8 | 6 | 0.948626 |
| *YME1L1* | | 8 | 6 | 0.948626 |
| *ZNF816* | | 8 | 6 | 0.948626 |
| *HMCN1* | | 36 | 38 | 0.951604 |
| *ATG2A* | | 7 | 8 | 0.951604 |
| *ATP13A3* | | 7 | 8 | 0.951604 |
| *BCL9* | | 7 | 8 | 0.951604 |
| *BLM* | | 7 | 8 | 0.951604 |
| *C12orf55* | | 9 | 7 | 0.951604 |
| *C3* | | 7 | 8 | 0.951604 |
| *CAMTA2* | | 7 | 8 | 0.951604 |
| *CCDC180* | | 9 | 7 | 0.951604 |
| *CDH10* | | 9 | 7 | 0.951604 |
| *CRB1* | | 9 | 7 | 0.951604 |
| *DDX3X* | | 7 | 8 | 0.951604 |
| *DYSF* | | 9 | 7 | 0.951604 |
| *FILIP1* | | 7 | 8 | 0.951604 |
| *HTT* | | 9 | 7 | 0.951604 |
| *KIAA2022* | | 7 | 8 | 0.951604 |
| *MAGI2* | | 9 | 7 | 0.951604 |
| *MET* | | 9 | 7 | 0.951604 |
| *MORC1* | | 9 | 7 | 0.951604 |
| *MPHOSPH9* | | 9 | 7 | 0.951604 |
| *MUC6* | | 9 | 7 | 0.951604 |
| *MYO18B* | | 9 | 7 | 0.951604 |
| *NBEAL1* | | 9 | 7 | 0.951604 |
| *NLRC5* | | 7 | 8 | 0.951604 |
| *NLRP7* | | 7 | 8 | 0.951604 |
| *NOTCH3* | | 7 | 8 | 0.951604 |
| *PCDHA12* | | 7 | 8 | 0.951604 |
| *PCDHB16* | | 9 | 7 | 0.951604 |
| *PCDHB5* | | 9 | 7 | 0.951604 |
| *PDGFRA* | | 7 | 8 | 0.951604 |
| *PTCHD2* | | 7 | 8 | 0.951604 |
| *PTPRF* | | 7 | 8 | 0.951604 |
| *RAI14* | | 9 | 7 | 0.951604 |
| *RARG* | | 7 | 8 | 0.951604 |
| *ROCK2* | | 9 | 7 | 0.951604 |
| *RPRD2* | | 7 | 8 | 0.951604 |
| *SMARCC2* | | 9 | 7 | 0.951604 |
| *SP140* | | 7 | 8 | 0.951604 |
| *THRAP3* | | 9 | 7 | 0.951604 |
| *TRHDE* | | 7 | 8 | 0.951604 |
| *TRIOBP* | | 7 | 8 | 0.951604 |
| *USP24* | | 7 | 8 | 0.951604 |
| *ZNF608* | | 7 | 8 | 0.951604 |
| *PIK3CA* | | 45 | 42 | 0.953374 |
| *ABCA8* | | 8 | 9 | 0.953374 |
| *ADAMTSL3* | | 8 | 9 | 0.953374 |
| *BRD2* | | 10 | 8 | 0.953374 |
| *C2CD3* | | 10 | 8 | 0.953374 |
| *CENPE* | | 10 | 8 | 0.953374 |
| *CEP250* | | 8 | 9 | 0.953374 |
| *CTNNA2* | | 10 | 8 | 0.953374 |
| *DOCK7* | | 8 | 9 | 0.953374 |
| *GCC2* | | 10 | 8 | 0.953374 |
| *GRIA4* | | 10 | 8 | 0.953374 |
| *GTF3C1* | | 8 | 9 | 0.953374 |
| *HEATR1* | | 10 | 8 | 0.953374 |
| *KIF15* | | 8 | 9 | 0.953374 |
| *LAMA5* | | 10 | 8 | 0.953374 |
| *MCM3AP* | | 8 | 9 | 0.953374 |
| *NIN* | | 8 | 9 | 0.953374 |
| *NINL* | | 8 | 9 | 0.953374 |
| *NUP155* | | 8 | 9 | 0.953374 |
| *PDE11A* | | 8 | 9 | 0.953374 |
| *POLR1A* | | 10 | 8 | 0.953374 |
| *PPFIA3* | | 8 | 9 | 0.953374 |
| *PRRC2A* | | 8 | 9 | 0.953374 |
| *PTPN4* | | 8 | 9 | 0.953374 |
| *RICTOR* | | 8 | 9 | 0.953374 |
| *RPGRIP1L* | | 10 | 8 | 0.953374 |
| *RSF1* | | 8 | 9 | 0.953374 |
| *SEMA3D* | | 10 | 8 | 0.953374 |
| *STIL* | | 8 | 9 | 0.953374 |
| *SUPT16H* | | 8 | 9 | 0.953374 |
| *TBC1D1* | | 8 | 9 | 0.953374 |
| *TEX14* | | 10 | 8 | 0.953374 |
| *TMEM132D* | | 8 | 9 | 0.953374 |
| *UNC5C* | | 8 | 9 | 0.953374 |
| *ZMYM4* | | 10 | 8 | 0.953374 |
| *ZNF318* | | 10 | 8 | 0.953374 |
| *ZNF521* | | 10 | 8 | 0.953374 |
| *ZNF626* | | 8 | 9 | 0.953374 |
| *DIAPH2* | | 11 | 9 | 0.959138 |
| *LRBA* | | 11 | 9 | 0.959138 |
| *MAGEC1* | | 11 | 9 | 0.959138 |
| *MXRA5* | | 11 | 9 | 0.959138 |
| *NALCN* | | 11 | 9 | 0.959138 |
| *SORCS1* | | 11 | 9 | 0.959138 |
| *STAG1* | | 11 | 9 | 0.959138 |
| *AKAP13* | | 9 | 10 | 0.959138 |
| *ASXL3* | | 9 | 10 | 0.959138 |
| *CACNA2D1* | | 9 | 10 | 0.959138 |
| *KMT2E* | | 9 | 10 | 0.959138 |
| *MED12L* | | 9 | 10 | 0.959138 |
| *PHLDB2* | | 9 | 10 | 0.959138 |
| *RHOA* | | 9 | 10 | 0.959138 |
| *RP1* | | 9 | 10 | 0.959138 |
| *TENM4* | | 9 | 10 | 0.959138 |
| *TRPM3* | | 9 | 10 | 0.959138 |
| *KMT2D* | | 56 | 58 | 0.963666 |
| *CDH23* | | 12 | 10 | 0.963666 |
| *EPHA5* | | 10 | 11 | 0.963666 |
| *FAM135B* | | 10 | 11 | 0.963666 |
| *FN1* | | 12 | 10 | 0.963666 |
| *HELZ* | | 12 | 10 | 0.963666 |
| *HSP90AA1* | | 10 | 11 | 0.963666 |
| *LTBP1* | | 10 | 11 | 0.963666 |
| *MYH10* | | 12 | 10 | 0.963666 |
| *PAPPA2* | | 12 | 10 | 0.963666 |
| *PTPRZ1* | | 10 | 11 | 0.963666 |
| *RIMS2* | | 12 | 10 | 0.963666 |
| *SF3B3* | | 10 | 11 | 0.963666 |
| *THSD7B* | | 10 | 11 | 0.963666 |
| *ZFHX3* | | 10 | 11 | 0.963666 |
| *ZNF536* | | 10 | 11 | 0.963666 |
| *HECTD1* | | 13 | 11 | 0.968703 |
| *KIF21A* | | 13 | 11 | 0.968703 |
| *SMCHD1* | | 13 | 11 | 0.968703 |
| *ATAD2* | | 11 | 12 | 0.968703 |
| *DMXL1* | | 11 | 12 | 0.968703 |
| *F8* | | 11 | 12 | 0.968703 |
| *LAMA4* | | 11 | 12 | 0.968703 |
| *MYO16* | | 11 | 12 | 0.968703 |
| *NSD1* | | 11 | 12 | 0.968703 |
| *ZFYVE9* | | 11 | 12 | 0.968703 |
| *CCDC88A* | | 14 | 12 | 0.974495 |
| *DOPEY2* | | 14 | 12 | 0.974495 |
| *ATRX* | | 12 | 13 | 0.974495 |
| *BAZ2B* | | 12 | 13 | 0.974495 |
| *AKAP6* | | 15 | 13 | 0.97951 |
| *HECTD4* | | 15 | 13 | 0.97951 |
| *PTPRD* | | 13 | 14 | 0.97951 |
| *TNXB* | | 15 | 13 | 0.97951 |
| *FLG2* | | 14 | 15 | 0.984743 |
| *DYNC1H1* | | 15 | 16 | 0.989149 |
| *TRIO* | | 17 | 15 | 0.989149 |
| *DMXL2* | | 18 | 16 | 0.993093 |
| *PCDH15* | | 16 | 17 | 0.993093 |
| *CDKN1A* | | 19 | 17 | 0.996349 |
| *NCOR1* | | 19 | 17 | 0.996349 |
| *TRRAP* | | 19 | 17 | 0.996349 |
| *ABCA13* | | 18 | 19 | 0.99955 |
| *DST* | | 18 | 19 | 0.99955 |
| *PDE4DIP* | | 22 | 23 | 1 |
| *TTN* | | 95 | 96 | 1 |
| *AGBL1* | | 7 | 6 | 1 |
| *AGO3* | | 5 | 5 | 1 |
| *AIM1* | | 5 | 5 | 1 |
| *AKNA* | | 7 | 6 | 1 |
| *ANKIB1* | | 7 | 6 | 1 |
| *ANKRD50* | | 5 | 5 | 1 |
| *APLP1* | | 5 | 5 | 1 |
| *ARAP2* | | 7 | 6 | 1 |
| *ATP1A2* | | 5 | 5 | 1 |
| *ATP2A1* | | 5 | 5 | 1 |
| *ATP8A2* | | 7 | 6 | 1 |
| *BBS9* | | 7 | 6 | 1 |
| *BCORL1* | | 5 | 5 | 1 |
| *BOC* | | 7 | 6 | 1 |
| *BRD8* | | 5 | 5 | 1 |
| *BUB1B* | | 5 | 5 | 1 |
| *C3orf20* | | 5 | 5 | 1 |
| *C6orf222* | | 5 | 5 | 1 |
| *C8orf76* | | 5 | 5 | 1 |
| *CACHD1* | | 5 | 5 | 1 |
| *CAT* | | 5 | 5 | 1 |
| *CC2D2A* | | 5 | 5 | 1 |
| *CCDC18* | | 7 | 6 | 1 |
| *CDC27* | | 5 | 5 | 1 |
| *CKMT2* | | 5 | 5 | 1 |
| *CLK2* | | 5 | 5 | 1 |
| *COBLL1* | | 7 | 6 | 1 |
| *COL9A1* | | 5 | 5 | 1 |
| *COLGALT2* | | 5 | 5 | 1 |
| *CPED1* | | 7 | 6 | 1 |
| *CPNE1* | | 5 | 5 | 1 |
| *CRIM1* | | 5 | 5 | 1 |
| *CROT* | | 5 | 5 | 1 |
| *CSMD1* | | 16 | 15 | 1 |
| *CUL4B* | | 5 | 5 | 1 |
| *CYP2A7* | | 5 | 5 | 1 |
| *DDX23* | | 5 | 5 | 1 |
| *DDX54* | | 5 | 5 | 1 |
| *DIS3L* | | 5 | 5 | 1 |
| *DLGAP1* | | 5 | 5 | 1 |
| *DSC1* | | 5 | 5 | 1 |
| *DSG1* | | 5 | 5 | 1 |
| *ELMO1* | | 5 | 5 | 1 |
| *ELN* | | 5 | 5 | 1 |
| *EML5* | | 7 | 6 | 1 |
| *EPC1* | | 5 | 5 | 1 |
| *EVPL* | | 5 | 5 | 1 |
| *EXOC8* | | 5 | 5 | 1 |
| *EYS* | | 5 | 5 | 1 |
| *FPGT* | | 5 | 5 | 1 |
| *GALNTL6* | | 5 | 5 | 1 |
| *GOLGB1* | | 16 | 15 | 1 |
| *GRIN2B* | | 5 | 5 | 1 |
| *GYS1* | | 5 | 5 | 1 |
| *HCN1* | | 11 | 11 | 1 |
| *HNRNPA2B1* | | 5 | 5 | 1 |
| *HSPA8* | | 5 | 5 | 1 |
| *IKBKB* | | 5 | 5 | 1 |
| *IMPG1* | | 5 | 5 | 1 |
| *IRS4* | | 7 | 6 | 1 |
| *ITGAE* | | 5 | 5 | 1 |
| *ITPR3* | | 5 | 5 | 1 |
| *IVNS1ABP* | | 5 | 5 | 1 |
| *JARID2* | | 5 | 5 | 1 |
| *KARS* | | 5 | 5 | 1 |
| *KDM2B* | | 7 | 6 | 1 |
| *KDM5B* | | 7 | 6 | 1 |
| *KIAA0232* | | 5 | 5 | 1 |
| *KIAA1211* | | 5 | 5 | 1 |
| *KIAA1841* | | 5 | 5 | 1 |
| *KIRREL* | | 5 | 5 | 1 |
| *LAMB3* | | 5 | 5 | 1 |
| *LILRA6* | | 5 | 5 | 1 |
| *LMO7* | | 5 | 5 | 1 |
| *LONP2* | | 5 | 5 | 1 |
| *LRP4* | | 5 | 5 | 1 |
| *LRRC37A3* | | 5 | 5 | 1 |
| *MADD* | | 5 | 5 | 1 |
| *MAP3K13* | | 7 | 6 | 1 |
| *MAST4* | | 5 | 5 | 1 |
| *MCCC1* | | 5 | 5 | 1 |
| *MERTK* | | 5 | 5 | 1 |
| *MPP6* | | 5 | 5 | 1 |
| *MRC2* | | 5 | 5 | 1 |
| *MTTP* | | 5 | 5 | 1 |
| *NFATC1* | | 7 | 6 | 1 |
| *NOTCH1* | | 7 | 6 | 1 |
| *NPEPPS* | | 5 | 5 | 1 |
| *NRAP* | | 7 | 6 | 1 |
| *OGDHL* | | 5 | 5 | 1 |
| *OLFM3* | | 5 | 5 | 1 |
| *OR8K3* | | 5 | 5 | 1 |
| *OXR1* | | 5 | 5 | 1 |
| *PAK3* | | 5 | 5 | 1 |
| *PBX2* | | 5 | 5 | 1 |
| *PCBP1* | | 5 | 5 | 1 |
| *PCDH12* | | 5 | 5 | 1 |
| *PCDHA4* | | 5 | 5 | 1 |
| *PCSK5* | | 7 | 6 | 1 |
| *PDS5B* | | 7 | 6 | 1 |
| *PKD2L1* | | 5 | 5 | 1 |
| *PLA2G4C* | | 5 | 5 | 1 |
| *PLCB3* | | 5 | 5 | 1 |
| *PLCE1* | | 5 | 5 | 1 |
| *PLXNA2* | | 7 | 6 | 1 |
| *POLA2* | | 7 | 6 | 1 |
| *PPIP5K2* | | 5 | 5 | 1 |
| *PPP1R10* | | 7 | 6 | 1 |
| *PPP4R4* | | 5 | 5 | 1 |
| *PRG4* | | 5 | 5 | 1 |
| *PSD2* | | 5 | 5 | 1 |
| *PUM1* | | 7 | 6 | 1 |
| *RAD50* | | 5 | 5 | 1 |
| *RAPGEF6* | | 5 | 5 | 1 |
| *RBL1* | | 5 | 5 | 1 |
| *RNF220* | | 5 | 5 | 1 |
| *RPTN* | | 5 | 5 | 1 |
| *SASH1* | | 5 | 5 | 1 |
| *SCAND3* | | 5 | 5 | 1 |
| *SESN1* | | 5 | 5 | 1 |
| *SIGLEC1* | | 7 | 6 | 1 |
| *SLC20A1* | | 5 | 5 | 1 |
| *SLC4A4* | | 5 | 5 | 1 |
| *SLC4A8* | | 5 | 5 | 1 |
| *SLC6A15* | | 5 | 5 | 1 |
| *SLCO1B3* | | 7 | 6 | 1 |
| *SLU7* | | 5 | 5 | 1 |
| *SPAG1* | | 5 | 5 | 1 |
| *SUPT5H* | | 5 | 5 | 1 |
| *SYNRG* | | 5 | 5 | 1 |
| *TGFBR3* | | 5 | 5 | 1 |
| *TLL1* | | 5 | 5 | 1 |
| *TLR5* | | 5 | 5 | 1 |
| *TOR1AIP1* | | 5 | 5 | 1 |
| *TSHZ3* | | 7 | 6 | 1 |
| *TTC17* | | 5 | 5 | 1 |
| *TUBGCP6* | | 5 | 5 | 1 |
| *TXNDC16* | | 5 | 5 | 1 |
| *UPF1* | | 7 | 6 | 1 |
| *USP33* | | 7 | 6 | 1 |
| *USP53* | | 5 | 5 | 1 |
| *VPS33A* | | 5 | 5 | 1 |
| *WNK2* | | 5 | 5 | 1 |
| *XPO6* | | 5 | 5 | 1 |
| *ZBTB38* | | 5 | 5 | 1 |
| *ZBTB49* | | 5 | 5 | 1 |
| *ZFYVE28* | | 5 | 5 | 1 |
| *ZMYM6* | | 5 | 5 | 1 |
| *ZNF329* | | 5 | 5 | 1 |
| *ZNF335* | | 5 | 5 | 1 |
| *ZNF429* | | 5 | 5 | 1 |
| *ZNF529* | | 5 | 5 | 1 |
| *ZNF548* | | 5 | 5 | 1 |
| *ZNF611* | | 7 | 6 | 1 |
| *ZNF616* | | 5 | 5 | 1 |
| *A2ML1* | | 6 | 6 | 1 |
| *ABCA2* | | 5 | 4 | 1 |
| *ABCA9* | | 8 | 7 | 1 |
| *ADAM17* | | 5 | 4 | 1 |
| *ADAMTS18* | | 10 | 9 | 1 |
| *ADAMTS2* | | 6 | 5 | 1 |
| *ADAMTS20* | | 9 | 8 | 1 |
| *ADAR* | | 6 | 6 | 1 |
| *ADCY8* | | 10 | 9 | 1 |
| *AFF1* | | 8 | 7 | 1 |
| *AFF3* | | 10 | 9 | 1 |
| *AGRN* | | 6 | 5 | 1 |
| *AHNAK* | | 21 | 20 | 1 |
| *AMER1* | | 6 | 5 | 1 |
| *ANAPC1* | | 8 | 8 | 1 |
| *ANAPC5* | | 6 | 5 | 1 |
| *ANKFY1* | | 6 | 5 | 1 |
| *ANKHD1* | | 15 | 15 | 1 |
| *ANKRD12* | | 11 | 10 | 1 |
| *ANKRD36* | | 6 | 5 | 1 |
| *AOAH* | | 6 | 5 | 1 |
| *AREL1* | | 5 | 4 | 1 |
| *ARFGEF1* | | 9 | 8 | 1 |
| *ARHGAP20* | | 7 | 7 | 1 |
| *ARHGAP5* | | 6 | 6 | 1 |
| *ARHGAP9* | | 6 | 6 | 1 |
| *ARID4A* | | 8 | 7 | 1 |
| *ARNT2* | | 5 | 4 | 1 |
| *ARPP21* | | 5 | 4 | 1 |
| *ASPM* | | 11 | 10 | 1 |
| *ATP12A* | | 9 | 9 | 1 |
| *ATP2B2* | | 6 | 6 | 1 |
| *ATP2B4* | | 7 | 7 | 1 |
| *ATRNL1* | | 5 | 4 | 1 |
| *ATXN2L* | | 5 | 4 | 1 |
| *AXIN2* | | 9 | 8 | 1 |
| *BAZ1A* | | 6 | 5 | 1 |
| *BCHE* | | 5 | 4 | 1 |
| *BICC1* | | 5 | 4 | 1 |
| *BRAF* | | 6 | 5 | 1 |
| *BRCA1* | | 10 | 10 | 1 |
| *BRDT* | | 6 | 6 | 1 |
| *BTAF1* | | 8 | 7 | 1 |
| *C10orf12* | | 6 | 6 | 1 |
| *C11orf30* | | 6 | 5 | 1 |
| *C9orf156* | | 5 | 4 | 1 |
| *CACNA1E* | | 9 | 8 | 1 |
| *CACNA1F* | | 6 | 5 | 1 |
| *CACNA1G* | | 8 | 8 | 1 |
| *CADPS* | | 9 | 9 | 1 |
| *CAMSAP1* | | 5 | 4 | 1 |
| *CAND1* | | 6 | 5 | 1 |
| *CASP8* | | 8 | 7 | 1 |
| *CBFA2T2* | | 6 | 6 | 1 |
| *CCDC129* | | 5 | 4 | 1 |
| *CD163L1* | | 6 | 6 | 1 |
| *CDC6* | | 5 | 4 | 1 |
| *CDH1* | | 7 | 7 | 1 |
| *CDH11* | | 7 | 7 | 1 |
| *CDH6* | | 7 | 7 | 1 |
| *CELSR3* | | 14 | 13 | 1 |
| *CENPT* | | 5 | 4 | 1 |
| *CHD1* | | 9 | 8 | 1 |
| *CHL1* | | 7 | 7 | 1 |
| *CLASP2* | | 7 | 7 | 1 |
| *CLMN* | | 5 | 4 | 1 |
| *CLSTN2* | | 6 | 5 | 1 |
| *CLTC* | | 9 | 9 | 1 |
| *CMYA5* | | 12 | 11 | 1 |
| *CNGB1* | | 6 | 6 | 1 |
| *CNNM4* | | 6 | 5 | 1 |
| *CNTNAP4* | | 10 | 10 | 1 |
| *CNTRL* | | 8 | 7 | 1 |
| *CNTROB* | | 5 | 4 | 1 |
| *COL25A1* | | 5 | 4 | 1 |
| *COL4A1* | | 6 | 6 | 1 |
| *COL7A1* | | 15 | 14 | 1 |
| *COPA* | | 6 | 6 | 1 |
| *CPT1C* | | 6 | 5 | 1 |
| *CROCC* | | 6 | 5 | 1 |
| *CTNND2* | | 9 | 9 | 1 |
| *CUX2* | | 7 | 7 | 1 |
| *DAPK1* | | 7 | 7 | 1 |
| *DCAF8L1* | | 5 | 4 | 1 |
| *DCHS1* | | 9 | 9 | 1 |
| *DDB1* | | 5 | 4 | 1 |
| *DDX17* | | 5 | 4 | 1 |
| *DDX4* | | 6 | 5 | 1 |
| *DDX58* | | 5 | 4 | 1 |
| *DDX60L* | | 5 | 4 | 1 |
| *DEPDC1* | | 5 | 4 | 1 |
| *DGKI* | | 5 | 4 | 1 |
| *DHX29* | | 5 | 4 | 1 |
| *DHX30* | | 5 | 4 | 1 |
| *DHX36* | | 5 | 4 | 1 |
| *DLEC1* | | 8 | 8 | 1 |
| *DNAH7* | | 20 | 20 | 1 |
| *DNAJC14* | | 5 | 4 | 1 |
| *DNM2* | | 7 | 7 | 1 |
| *DOCK10* | | 8 | 7 | 1 |
| *DSCAML1* | | 11 | 10 | 1 |
| *DSG2* | | 5 | 4 | 1 |
| *DXO* | | 5 | 4 | 1 |
| *DZIP1* | | 6 | 6 | 1 |
| *E2F8* | | 8 | 7 | 1 |
| *ECM2* | | 5 | 4 | 1 |
| *EEA1* | | 7 | 7 | 1 |
| *EGF* | | 9 | 9 | 1 |
| *EIF4A3* | | 5 | 4 | 1 |
| *EIF4G3* | | 13 | 12 | 1 |
| *EML1* | | 5 | 4 | 1 |
| *EP400* | | 12 | 11 | 1 |
| *EPHA7* | | 6 | 5 | 1 |
| *EPPK1* | | 6 | 6 | 1 |
| *ERBB3* | | 21 | 21 | 1 |
| *ERN2* | | 6 | 5 | 1 |
| *ESF1* | | 6 | 6 | 1 |
| *ETV6* | | 6 | 5 | 1 |
| *EXPH5* | | 12 | 11 | 1 |
| *FAM47B* | | 5 | 4 | 1 |
| *FANCA* | | 9 | 9 | 1 |
| *FAT1* | | 24 | 24 | 1 |
| *FBLN1* | | 5 | 4 | 1 |
| *FBN3* | | 13 | 13 | 1 |
| *FBXO34* | | 6 | 6 | 1 |
| *FER1L6* | | 10 | 10 | 1 |
| *FERMT3* | | 7 | 7 | 1 |
| *FGA* | | 9 | 8 | 1 |
| *FMO3* | | 6 | 5 | 1 |
| *FOLH1* | | 5 | 4 | 1 |
| *FREM1* | | 9 | 9 | 1 |
| *FRYL* | | 10 | 9 | 1 |
| *FTSJ3* | | 5 | 4 | 1 |
| *FUS* | | 5 | 4 | 1 |
| *GABRG1* | | 5 | 4 | 1 |
| *GAK* | | 6 | 6 | 1 |
| *GGNBP2* | | 5 | 4 | 1 |
| *GNA13* | | 6 | 6 | 1 |
| *GOLIM4* | | 5 | 4 | 1 |
| *GPR112* | | 12 | 12 | 1 |
| *GPR133* | | 6 | 6 | 1 |
| *GPR179* | | 9 | 9 | 1 |
| *GREB1* | | 7 | 7 | 1 |
| *GRID1* | | 6 | 5 | 1 |
| *GRIK3* | | 7 | 7 | 1 |
| *GRM4* | | 5 | 4 | 1 |
| *HAUS6* | | 6 | 6 | 1 |
| *HEPH* | | 6 | 6 | 1 |
| *HES1* | | 6 | 5 | 1 |
| *HGF* | | 6 | 6 | 1 |
| *HGS* | | 6 | 5 | 1 |
| *HIVEP1* | | 9 | 8 | 1 |
| *HK1* | | 6 | 5 | 1 |
| *HK2* | | 6 | 5 | 1 |
| *HNRNPU* | | 5 | 4 | 1 |
| *HRAS* | | 9 | 8 | 1 |
| *HRC* | | 5 | 4 | 1 |
| *HTATSF1* | | 5 | 4 | 1 |
| *IDH1* | | 5 | 4 | 1 |
| *IL16* | | 6 | 5 | 1 |
| *IMMT* | | 5 | 4 | 1 |
| *IQCB1* | | 6 | 5 | 1 |
| *IQCH* | | 5 | 4 | 1 |
| *ITGA8* | | 9 | 8 | 1 |
| *ITGAV* | | 7 | 7 | 1 |
| *ITPR2* | | 15 | 15 | 1 |
| *ITSN2* | | 9 | 9 | 1 |
| *JAG1* | | 6 | 6 | 1 |
| *JAK3* | | 6 | 5 | 1 |
| *JHDM1D* | | 5 | 4 | 1 |
| *KCNH3* | | 5 | 4 | 1 |
| *KCNH8* | | 7 | 7 | 1 |
| *KIAA0753* | | 5 | 4 | 1 |
| *KIAA1432* | | 6 | 5 | 1 |
| *KIAA2018* | | 15 | 14 | 1 |
| *KIF17* | | 6 | 5 | 1 |
| *KIFAP3* | | 7 | 7 | 1 |
| *KLF6* | | 5 | 4 | 1 |
| *KLHL11* | | 5 | 4 | 1 |
| *KNTC1* | | 11 | 10 | 1 |
| *KRT84* | | 5 | 4 | 1 |
| *L3MBTL1* | | 5 | 4 | 1 |
| *LPHN3* | | 10 | 10 | 1 |
| *LRP6* | | 9 | 8 | 1 |
| *LRRC66* | | 7 | 7 | 1 |
| *LRRC7* | | 13 | 13 | 1 |
| *LRRN1* | | 6 | 6 | 1 |
| *MAGI1* | | 8 | 7 | 1 |
| *MAP1B* | | 9 | 9 | 1 |
| *MAP2* | | 7 | 7 | 1 |
| *MAP3K4* | | 10 | 9 | 1 |
| *MAP3K5* | | 8 | 7 | 1 |
| *MAPK10* | | 5 | 4 | 1 |
| *10-Mar* | | 5 | 4 | 1 |
| *MATR3* | | 5 | 4 | 1 |
| *MDC1* | | 8 | 7 | 1 |
| *MGAM* | | 6 | 6 | 1 |
| *MIB1* | | 5 | 4 | 1 |
| *MIS18BP1* | | 6 | 6 | 1 |
| *MOCOS* | | 5 | 4 | 1 |
| *MUC3A* | | 8 | 7 | 1 |
| *MUC4* | | 12 | 11 | 1 |
| *MUC5B* | | 16 | 16 | 1 |
| *MYF5* | | 5 | 4 | 1 |
| *MYH9* | | 14 | 14 | 1 |
| *MYO15A* | | 10 | 9 | 1 |
| *MYO1A* | | 8 | 7 | 1 |
| *MYO1E* | | 5 | 4 | 1 |
| *MYO5A* | | 6 | 6 | 1 |
| *N4BP2L2* | | 6 | 6 | 1 |
| *NARG2* | | 6 | 6 | 1 |
| *NAV1* | | 8 | 7 | 1 |
| *NBAS* | | 7 | 7 | 1 |
| *NBPF10* | | 5 | 4 | 1 |
| *NCOA1* | | 11 | 10 | 1 |
| *NCOR2* | | 8 | 8 | 1 |
| *NELL2* | | 5 | 4 | 1 |
| *NES* | | 10 | 9 | 1 |
| *NHLRC1* | | 5 | 4 | 1 |
| *NLRP14* | | 5 | 4 | 1 |
| *NLRP5* | | 6 | 6 | 1 |
| *NOA1* | | 6 | 5 | 1 |
| *NOS3* | | 6 | 5 | 1 |
| *NPAP1* | | 6 | 6 | 1 |
| *NPAT* | | 6 | 6 | 1 |
| *NSUN7* | | 5 | 4 | 1 |
| *NUP160* | | 6 | 5 | 1 |
| *NUP210* | | 8 | 7 | 1 |
| *NUP210L* | | 8 | 7 | 1 |
| *NUP93* | | 5 | 4 | 1 |
| *OR10Z1* | | 5 | 4 | 1 |
| *OTOGL* | | 10 | 10 | 1 |
| *OTUD4* | | 5 | 4 | 1 |
| *PAIP1* | | 6 | 5 | 1 |
| *PARP1* | | 6 | 5 | 1 |
| *PCDH10* | | 8 | 7 | 1 |
| *PCDH18* | | 5 | 4 | 1 |
| *PCDH20* | | 7 | 7 | 1 |
| *PCDHB14* | | 6 | 5 | 1 |
| *PCDHGA5* | | 8 | 8 | 1 |
| *PCDHGB3* | | 6 | 6 | 1 |
| *PCF11* | | 9 | 9 | 1 |
| *PCNXL2* | | 10 | 10 | 1 |
| *PDE5A* | | 6 | 5 | 1 |
| *PDZD2* | | 12 | 12 | 1 |
| *PEG3* | | 13 | 12 | 1 |
| *PGBD1* | | 5 | 4 | 1 |
| *PIKFYVE* | | 8 | 8 | 1 |
| *PIP5K1C* | | 5 | 4 | 1 |
| *PLA2G4B* | | 5 | 4 | 1 |
| *PLA2R1* | | 9 | 8 | 1 |
| *PLEKHH2* | | 11 | 10 | 1 |
| *PLXNA4* | | 12 | 12 | 1 |
| *PLXNB2* | | 12 | 11 | 1 |
| *PNPLA7* | | 5 | 4 | 1 |
| *PPARG* | | 7 | 7 | 1 |
| *PPARGC1A* | | 8 | 8 | 1 |
| *PPL* | | 10 | 10 | 1 |
| *PPP1R12B* | | 5 | 4 | 1 |
| *PREX2* | | 11 | 10 | 1 |
| *PRKCI* | | 5 | 4 | 1 |
| *PROX1* | | 5 | 4 | 1 |
| *PSD4* | | 5 | 4 | 1 |
| *PSG2* | | 5 | 4 | 1 |
| *PTPRJ* | | 5 | 4 | 1 |
| *PZP* | | 8 | 8 | 1 |
| *RAI1* | | 10 | 10 | 1 |
| *RALGAPA2* | | 8 | 7 | 1 |
| *RB1CC1* | | 11 | 10 | 1 |
| *RBM26* | | 10 | 10 | 1 |
| *RBM39* | | 5 | 4 | 1 |
| *RET* | | 5 | 4 | 1 |
| *RIMS1* | | 6 | 6 | 1 |
| *RNF111* | | 8 | 8 | 1 |
| *RNF19A* | | 7 | 7 | 1 |
| *RPS6KA5* | | 6 | 5 | 1 |
| *RPTOR* | | 6 | 6 | 1 |
| *RTN1* | | 5 | 4 | 1 |
| *RYR1* | | 23 | 23 | 1 |
| *SAMD15* | | 5 | 4 | 1 |
| *SCAF8* | | 6 | 5 | 1 |
| *SCN4A* | | 6 | 5 | 1 |
| *SCN7A* | | 5 | 4 | 1 |
| *SCN9A* | | 7 | 7 | 1 |
| *SEMA4A* | | 5 | 4 | 1 |
| *SETBP1* | | 6 | 5 | 1 |
| *SGK223* | | 5 | 4 | 1 |
| *SH3BP4* | | 6 | 5 | 1 |
| *SIM1* | | 6 | 5 | 1 |
| *SLC22A12* | | 6 | 5 | 1 |
| *SLC27A2* | | 6 | 5 | 1 |
| *SLC28A3* | | 6 | 5 | 1 |
| *SLC38A10* | | 6 | 6 | 1 |
| *SLC38A2* | | 7 | 7 | 1 |
| *SLC5A9* | | 5 | 4 | 1 |
| *SLCO1C1* | | 6 | 5 | 1 |
| *SMC1A* | | 8 | 7 | 1 |
| *SMC2* | | 8 | 7 | 1 |
| *SMPD4* | | 5 | 4 | 1 |
| *SMURF2* | | 5 | 4 | 1 |
| *SPATA5* | | 8 | 7 | 1 |
| *SPTA1* | | 21 | 21 | 1 |
| *SPTBN5* | | 7 | 7 | 1 |
| *SRGAP2* | | 5 | 4 | 1 |
| *SRP68* | | 6 | 6 | 1 |
| *STAB1* | | 9 | 8 | 1 |
| *STK36* | | 6 | 5 | 1 |
| *SUCO* | | 6 | 5 | 1 |
| *SUZ12* | | 5 | 4 | 1 |
| *SWT1* | | 5 | 4 | 1 |
| *TACC2* | | 10 | 9 | 1 |
| *TAF1* | | 6 | 5 | 1 |
| *TANC2* | | 9 | 9 | 1 |
| *TAOK1* | | 6 | 6 | 1 |
| *TBX5* | | 6 | 5 | 1 |
| *TCEB3B* | | 6 | 6 | 1 |
| *TGM3* | | 5 | 4 | 1 |
| *THSD4* | | 5 | 4 | 1 |
| *THSD7A* | | 6 | 6 | 1 |
| *TJP2* | | 6 | 5 | 1 |
| *TLN1* | | 10 | 9 | 1 |
| *TMEM200A* | | 6 | 5 | 1 |
| *TMPRSS15* | | 6 | 6 | 1 |
| *TNC* | | 9 | 8 | 1 |
| *TNN* | | 8 | 8 | 1 |
| *TNRC6B* | | 6 | 5 | 1 |
| *TPP2* | | 8 | 7 | 1 |
| *TPR* | | 13 | 12 | 1 |
| *TRANK1* | | 13 | 12 | 1 |
| *TRAPPC12* | | 5 | 4 | 1 |
| *TRIM24* | | 6 | 6 | 1 |
| *TROAP* | | 5 | 4 | 1 |
| *TRPM6* | | 10 | 10 | 1 |
| *TTC13* | | 5 | 4 | 1 |
| *TULP4* | | 8 | 7 | 1 |
| *TXNDC11* | | 5 | 4 | 1 |
| *UBR2* | | 8 | 7 | 1 |
| *ULK2* | | 5 | 4 | 1 |
| *ULK4* | | 6 | 5 | 1 |
| *USP28* | | 6 | 5 | 1 |
| *USP4* | | 6 | 6 | 1 |
| *USP7* | | 8 | 7 | 1 |
| *VCPIP1* | | 6 | 6 | 1 |
| *VWA3B* | | 8 | 7 | 1 |
| *WAPAL* | | 6 | 5 | 1 |
| *WDR6* | | 6 | 6 | 1 |
| *WDR62* | | 7 | 7 | 1 |
| *WDR66* | | 6 | 6 | 1 |
| *WDR7* | | 5 | 4 | 1 |
| *WHSC1* | | 7 | 7 | 1 |
| *XPO5* | | 5 | 4 | 1 |
| *ZBTB22* | | 6 | 5 | 1 |
| *ZC3HC1* | | 5 | 4 | 1 |
| *ZCCHC11* | | 8 | 7 | 1 |
| *ZCCHC6* | | 10 | 9 | 1 |
| *ZFP57* | | 5 | 4 | 1 |
| *ZIM3* | | 6 | 5 | 1 |
| *ZMYM1* | | 5 | 4 | 1 |
| *ZNF107* | | 6 | 5 | 1 |
| *ZNF180* | | 8 | 7 | 1 |
| *ZNF207* | | 5 | 4 | 1 |
| *ZNF225* | | 5 | 4 | 1 |
| *ZNF337* | | 5 | 4 | 1 |
| *ZNF423* | | 10 | 9 | 1 |
| *ZNF486* | | 5 | 4 | 1 |
| *ZNF507* | | 9 | 8 | 1 |
| *ZNF511* | | 5 | 4 | 1 |
| *ZNF513* | | 6 | 5 | 1 |
| *ZNF607* | | 6 | 5 | 1 |
| *ZNF665* | | 6 | 6 | 1 |
| *ZNF714* | | 5 | 4 | 1 |
| *ZNF777* | | 6 | 5 | 1 |
| *ZNF829* | | 5 | 4 | 1 |
| *ZRANB3* | | 6 | 5 | 1 |
| *ZSCAN22* | | 5 | 4 | 1 |
| *ZSWIM8* | | 8 | 8 | 1 |

*: Adjust p<0.05 was considered as statistically significant.
